# Synthesis of Benzoic Acids from Electrochemically Reduced CO_2_ Using Heterogeneous Catalysts

**DOI:** 10.1002/cssc.202401084

**Published:** 2024-11-05

**Authors:** Ha Phan, Robin Gueret, Pablo Martínez‐Pardo, Alejandro Valiente, Aleksander Jaworski, Adam Slabon, Belén Martín‐Matute

**Affiliations:** ^1^ Department of Organic Chemistry Arrhenius Laboratory Stockholm University SE-106 91 Stockholm Sweden; ^2^ Department of Materials and Environmental Chemistry Arrhenius Laboratory Stockholm University SE-106 91 Stockholm Sweden; ^3^ Faculty of Mathematics and Natural Sciences Chair of Inorganic Chemistry University of Wuppertal Gaußstraße 20 42219 Wuppertal Germany

**Keywords:** Metal-Organic Frameworks, Palladium, Electrochemistry, Carbon dioxide fixation, Carbonylation.

## Abstract

A method for the synthesis of benzoic acids from aryl iodides using two of the most abundant and sustainable feedstocks, carbon dioxide (CO_2_) and water, is disclosed. Central to this method is an effective and selective electrochemical reduction of CO_2_ (eCO_2_RR) to CO, which mitigates unwanted dehalogenation reactions occurring when H_2_ is produced *via* the hydrogen evolution reaction (HER). In a 3‐compartment set‐up, CO_2_ was reduced to CO electrochemically by using a surface‐modified silver electrode in aqueous electrolyte. The *ex‐situ* generated CO further underwent hydroxycarbonylation of aryl iodides by MOF‐supported palladium catalyst in excellent yields at room temperature. The method avoids the direct handling of hazardous CO gas and gives a wide range of benzoic acid derivatives. Both components of the tandem system can be recycled for several consecutive runs while keeping a high catalytic activity.

## Graphical abstract

The synthesis of benzoic acids from aryl iodides using two of the most abundant and sustainable feedstocks, carbon dioxide (CO_2_) and water. In a 3‐compartment set‐up, CO_2_ was reduced to CO electrochemically by using a surface‐modified silver electrode and used in the hydroxycarbonylation of aryl iodides by a MOF‐supported palladium catalyst in excellent yields at room temperature.

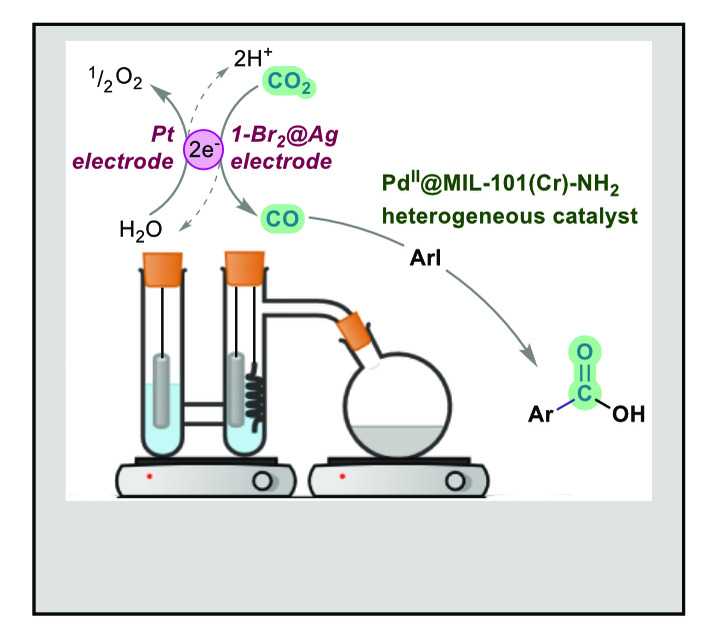

## Introduction

CO_2_ constitutes an abundant source of carbon for the chemical synthesis of organic compounds, and can contribute to reduce the consumption of other scarce resources.^[1]^ The integration of air CO_2_ capture technology powered by renewable energy, enables the production of chemicals and fuels in a sustainable manner, embracing the concept of “air economy”.^[3]^ Hence, in recent years the use of CO_2_ as a C1 synthon has been the subject of many studies.^[4]^ Among them, the electrocatalytic conversion of CO_2_ to different reduced forms is considered as a viable approach to close the carbon cycle.^[8]^ High Faradaic efficiencies can be reached towards C_1_ products, such as CO,^[10]^ formate,^[15]^ or even CH_4_.^[20]^ Recent studies have also shown high Faradaic efficiencies for C_2+_ products, further demonstrating the promising potential of electrochemical reduction of CO_2_.^[23]^

Carboxylic acids are structural motifs found in various natural and pharmaceutically active compounds. The carboxylic function is present in roughly 25 % of all commercialized drugs and at least 40 % of marketed plant protection agents.^[26]^ The carboxylic acid moiety can serve as a handle for further functionalization via transition‐metal catalyzed late‐stage functionalizations (LSFs) of known drugs, as we recently demonstrated.^[29]^

**Scheme 1:**
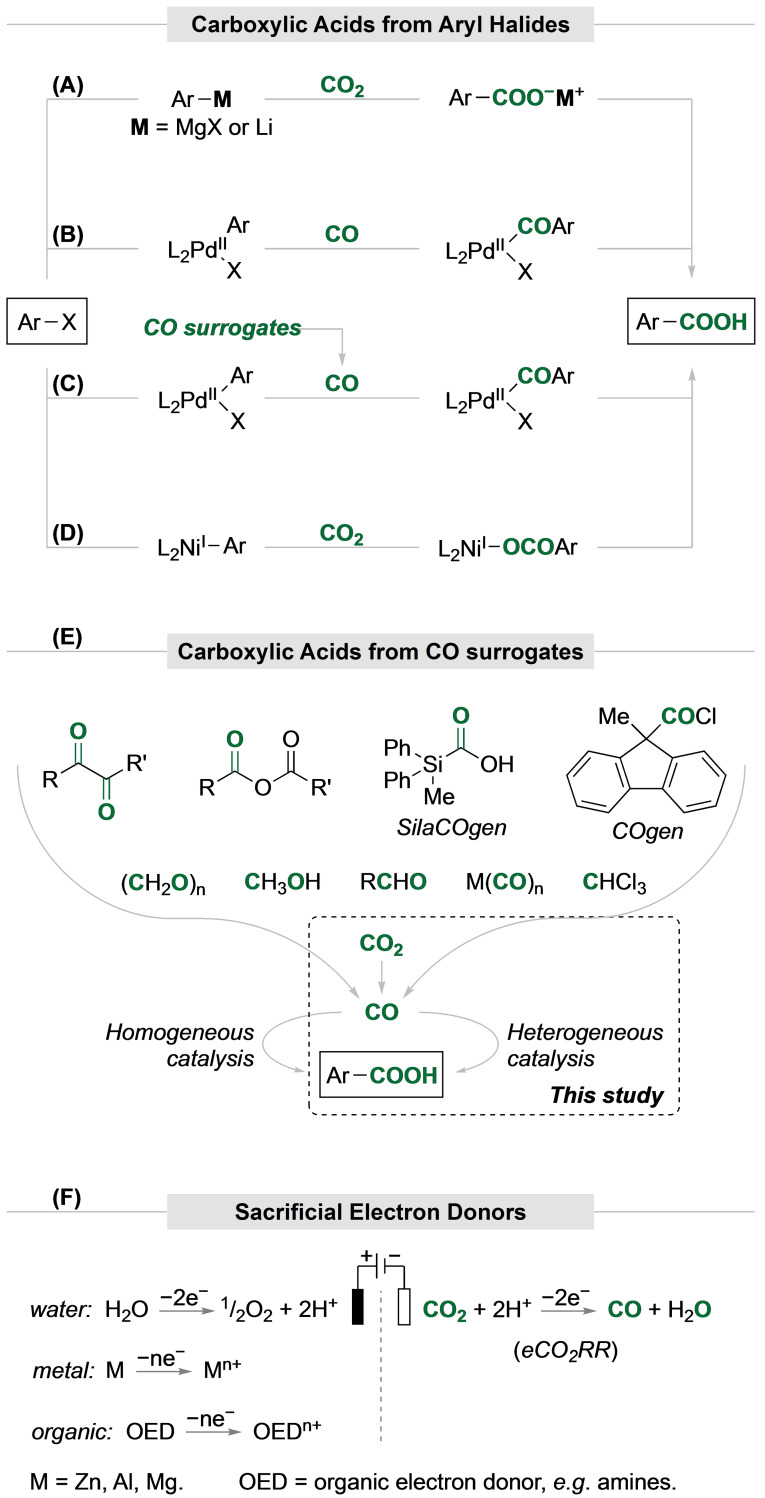
Synthetic pathways to afford carboxylic acids from aryl halides and CO or CO surrogates.

Benzoic acids can be prepared by reacting aromatic Grignard or organolithium reagents with CO_2_, yielding benzoates (Scheme 1A).^[33]^ This method requires anhydrous, cryogenic conditions as well as careful handling, and has low functional group tolerance. It also produces stoichiometric amounts of salts.

Alternatively, transition‐metal‐catalyzed carbonylation of haloarenes in the presence of water affords benzoic acids (Scheme 1B). Here, homogeneous palladium catalysts, with phosphines or nitrogenated ligands, commonly under high pressure of CO gas, can be used.^[34]^ The Martín‐Matute group reported the use of palladium nanoparticles immobilized on a metal‐organic framework (MOF), namely Pd^0^@MIL‐88B(Fe/Cr)‐NH_2_, as heterogeneous catalysts for carbonylation reactions.^[36]^ This catalyst provided a good scope in alkoxycarbonylation (synthesis of esters) and aminocarbonylation (synthesis of amides) reactions under CO atmosphere. However, when water was introduced as the nucleophile to promote the hydroxycarbonylation reaction (synthesis of benzoic acids), low yields were obtained due to formation of symmetrical anhydrides, and other byproducts. Further, the need to use Cs_2_CO_3_ as a base hindered catalyst recyclability. The system was found to work best in aqueous HFIP solutions and at 95 °C.

To avoid direct handling of the toxic and flammable CO gas, CO surrogates can be used as a source of carbon monoxide in transition‐metal‐catalyzed hydroxycarbonylations of aryl halides (Scheme 1C)^[37]^; CO surrogates include methanol,^[40]^ formates,^[41]^ formamides,^[42]^ formic acid,^[44]^ anhydrides,^[46]^ aldehydes,^[47]^ chloroform,^[49]^ metal carbonyls,^[50]^ 9‐methylfluorene‐9‐carbonyl chloride (COgen),^[52]^ methyldiphenylsilanecarboxylic acid (SilaCOgen),^[53]^ and CO_2_ ^[54]^ (Scheme 1E). The surrogates provide CO under diverse conditions, including reductive treatment, exposure to heat, or other stimuli. The direct use of CO_2_ as a CO source is undoubtedly the most atom‐economical option. For example, reductive carboxylation of aryl halides using transition metal catalysis (e.g. Ni or Pd) in combination with (super)stoichiometric amounts of reductants, such as Mn or Zn, enables direct hydroxycarboxylation of aryl halides (Scheme 1D).^[55]^

Electrochemistry has become an emerging area of research enabling a large array of organic transformations.^[57]^ Recent advances in electrochemical carboxylation have been recently reviewed.^[61]^ Electrochemistry can be used to abstract the electrons from sacrificial electron donors (EDs) in the anodic chamber for reduction of CO_2_–CO in the cathodic chamber (Scheme 1F). Compared to metallic and organic EDs, water stands out as the most attractive sacrificial ED but challenging due to its very high oxidation potential ($E_{O_{2}/H_{2}O}^{0}$ =+1.23 V *vs* NHE). Furthermore, the hydrogen evolution reaction is competing with eCO_2_RR under electrochemical conditions, resulting in mixtures of CO, H_2_, and CO_2_ gases. The hydrogen gas (H_2_) affects the efficiency and the effectiveness of the Pd‐catalyzed carbonylation reactions, promoting dehalogenation of the aryl halide substrates.^[63]^ Despite these challenges, it is noteworthy that the water oxidation reaction provides the electrons and protons needed for eCO_2_RR, while generating O_2_ as the sole byproduct.

Herein, we describe the synthesis of benzoic acids mediated by a heterogeneous catalyst, namely Pd^II^@MIL‐101(Cr)‐NH_2_, *via* hydroxycarbonylation of aryl iodides with electrochemically reduced CO_2_. CO is generated *ex‐situ* from eCO_2_RR using water as an ED. The challenges combining eCO_2_RR with Pd‐catalyzed reactions are discussed, as well as the recyclability of both carbonylation catalyst and the functionalized electrode.

## Results and Discussion

**Scheme 2:**
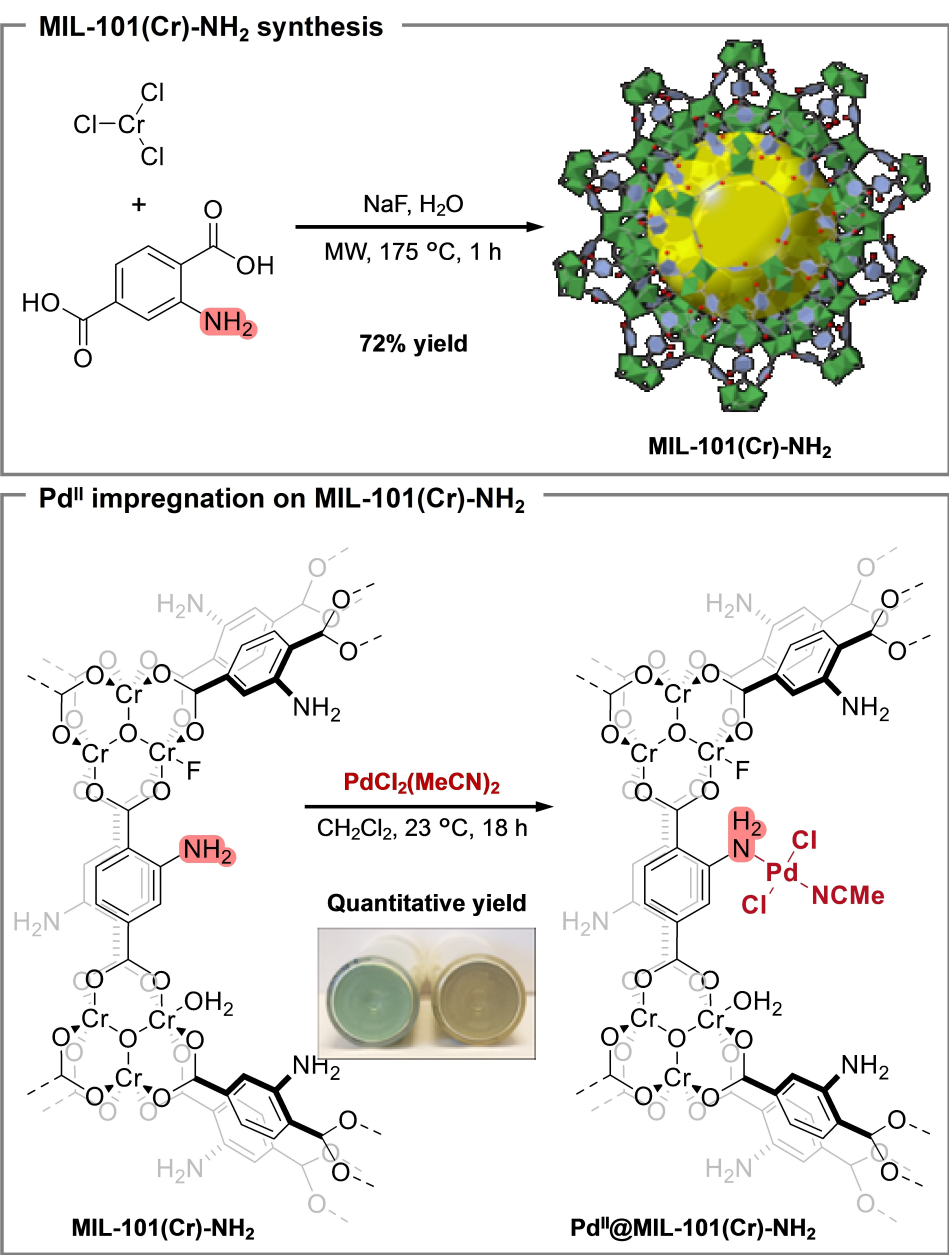
Microwave (MW)‐assisted synthesis of MIL‐101(Cr)‐NH_2_ and palladium(II) impregnation.

Considering the limitations in the hydroxycarbonylation reactions catalyzed by Pd^0^@MIL‐88B‐NH_2_(Fe) under a CO atmosphere (*vide supra*),^[36]^ our attention now shifted towards evaluating the viability of utilizing the robust MIL‐101(Cr)‐NH_2_ as the catalyst. Further, we have recently developed a rapid and high‐yielding method for the synthesis of MIL‐101(Cr)‐NH_2_ in a single synthetic step (Scheme 2), which enables very rapid access to this catalyst, facilitating catalyst screening.^[64]^ The amino functional groups, apart from being the binding sites for Pd^II^, can also stabilize Pd^0^ intermediate species within the MOF material during catalysis.[^65^, ^66^]

The microwave method used for the synthesis of MIL‐101(Cr)‐NH_2_ from CrCl_3_ and 2‐aminoterephthalic acid (2‐ATA) (Scheme 2) afforded the target MOF MIL‐101(Cr)‐NH_2_ in 72 % yield within 1 h. The as‐synthesized MIL‐101(Cr)‐NH_2_ was then washed with water several times before being activated at 60 °C under vacuum overnight. Pd^II^@MIL‐101(Cr)‐NH_2_ (8 wt % of Pd content) was then obtained by exposing the activated MIL‐101(Cr)‐NH_2_ to a solution of Pd(MeCN)_2_Cl_2_ in CH_2_Cl_2_ (see S.I. 2.3), and was used for further investigations in this study.

The carbonylation reaction of *p‐*iodotoluene (**1 a**, Table 1) catalyzed by Pd^II^@MIL‐101(Cr)‐NH_2_ was first optimized under a CO atmosphere. A combination of NaHCO_3_ (5 equiv.) and triethyl amine (TEA, 10 equiv.) using 4.6 mol % of Pd loading gave full conversion of **1 a** into **2 a** at 30 °C (Table 1, entry 1). Lowering the catalyst loading to 2.3 mol % and 1.2 mol % resulted in similar conversions but lower yields (Table 1, entries 2–3). The difference between conversions and yields is a result of unwanted Pd‐mediated dehalogenation reactions under the reaction conditions, resulting in formation of toluene from **1 a**.^[68]^ To tackle this issue, the combined used of NaHCO_3_ and TEA turned out to be crucial, as in the absence of either, a considerable drop in reaction yields occurred, despite the excellent conversions (Table 1, entries 4–6). It has been shown by Lei and co‐workers that the nature of the base plays a key role in the reaction mechanism of Pd‐catalyzed carbonylation reaction of aryl iodides, as well as in the rate and selectivities observed.^[70]^ The reaction gives a very good yield of **2 a** after only 2 h (entry 7, 93 % yield). Other common Pd sources were evaluated, under otherwise identical reaction conditions. However, in these instances, the hydrodeiodination, producing toluene, was not prevented (Table 1, entries 8–9). Further, after the carbonylation of **1 a** using Pd(OAc)_2_ or Pd(MeCN)_2_Cl_2_ as catalyst, formation of black palladium clusters occurred. Using Pd/C as the catalyst resulted in a very low yield of 19 % (Table 1, entry 10).

**Table 1:** Reaction outcome at different conditions.

Entry	Deviation from standard conditions^[a]^	Conv. [%]	Yield **2 a** [%]
1	None	>99	>99
2	2.3 mol % Pd loading	>99	88
3	1.2 mol % Pd loading	>99	63
4	Absence of TEA	>99	63
5	Absence of NaHCO_3_	>99	86
6	TEA (15 equiv.), no NaHCO_3_	>99	91
7^[b]^	2 h instead of 16 h	97	93
8^[b]^	Pd(OAc)_2_ (4.6 mol %)	>99	66
9^[b]^	PdCl_2_(MeCN)_2_ (4.6 mol %)	90	87
10^[b]^	Pd/C (4.6 mol % Pd)	>99	19
11^[c]^	**1 a** (5.0 mmol)	>99	67

[a] Unless otherwise stated: **1 a** (0.25 mmol), Pd^II^@MIL‐101(Cr)‐NH_2_ (4.6 mol % based on Pd), NaHCO_3_ (5 equiv.), TEA (10 equiv.), 1,4‐dioxane: H_2_O (*v/v*, 1 : 3, 12.5 mL), CO (balloon, 1 atm), 30 °C, 16 h. Yields and conversions were determined by ^1^H NMR spectroscopy using 1,3,5‐trimethoxybenzene as an internal standard. [b] 2 h. [c] scale‐up: **1 a** (5.0 mmol), Pd^II^@MIL‐101(Cr)‐NH_2_ (2.3 mol % based on Pd), isolated yield is reported. See the Supporting Information, Scheme S4.

**Figure 1:**
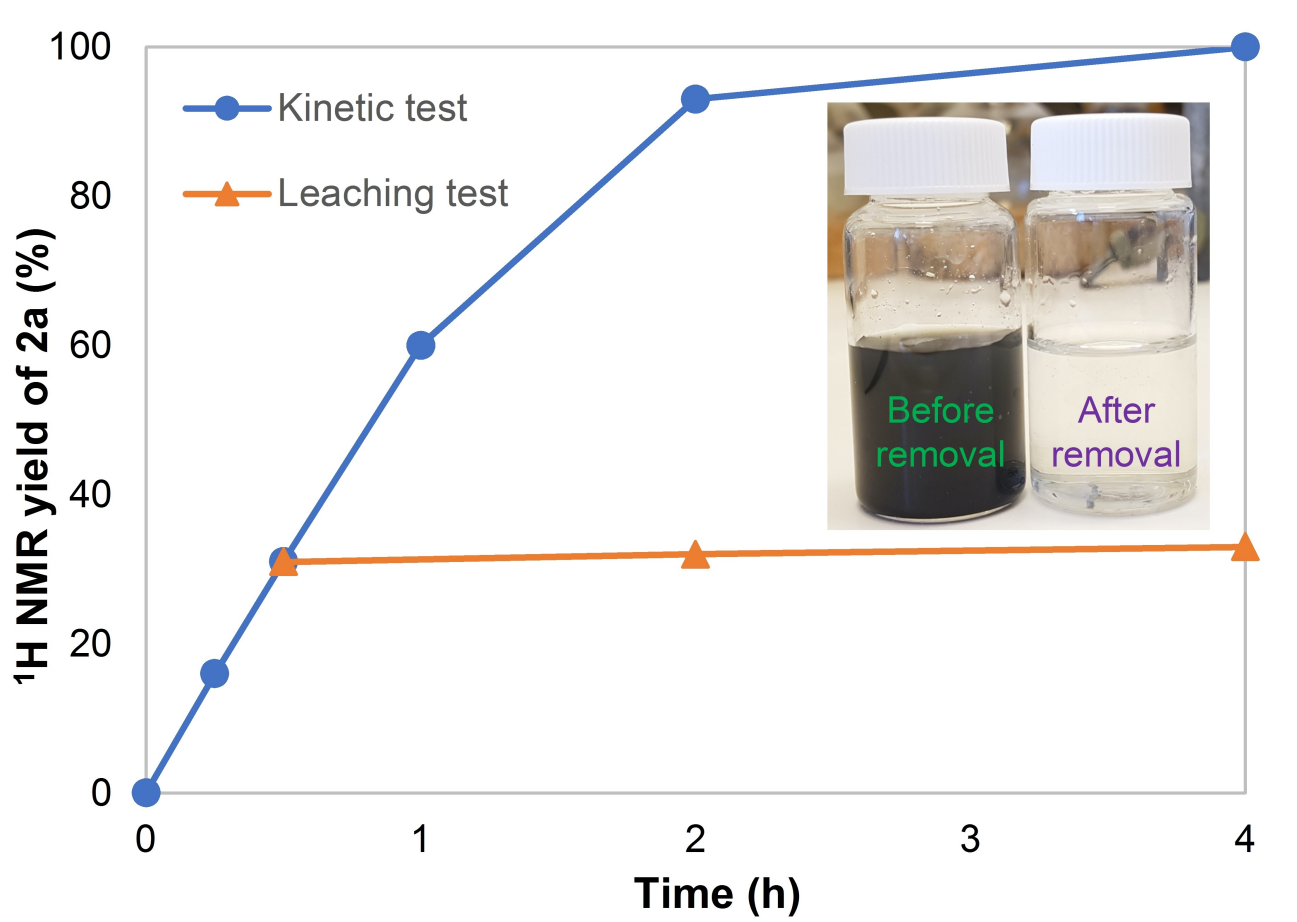
Hydroxycarbonylation of **1 a** under CO atmosphere by Pd^II^@MIL‐101(Cr)‐NH_2_ (blue circles), and after removing the catalyst after 0.5 h reaction time (orange triangles).[a] Reaction conditions as in Table 1, entry 1, time varied. Insert shows the appearance of reaction mixture before and after removing the solid catalyst.

Figure 1 shows the yield of **2 a** as the reaction proceeds. After only 1 h, **2 a** is formed in 60 % yield, and the reaction is completed within 4 h. In a parallel experiment, after 0.5 h of reaction time, the catalyst was removed. First, the mixture was centrifuged, and then filtered off through a 0.45 μm syringe filter (see the Supporting Information 4.2 for details). The filtrate was kept under the reaction conditions for an additional 3.5 h, which resulted in a negligible increase of yield of **2 a** (from 31 % after 0.5 h to 33 % after 4 h). This indicates that the leaching of active Pd species into the solution is minimum. Further, a clear solution is obtained after removal of the catalyst (Figure 1), indicating the outstanding properties of the MOF as a palladium support. The used catalysts were collected and analyzed by powder X‐ray diffraction (PXRD), which indicated formation of Pd clusters with increasing sizes, up to 23 nm, as the reaction proceeds (Figure S1).

We went on by performing the reaction using CO produced through eCO_2_RR in an aqueous environment, implying the possibility of simultaneous HER. To maximize CO concentration and minimize H_2_ production, which leads to unwanted Pd–H species responsible for hydrodeiodination,^[68]^ the choice of the eCO_2_RR electrocatalyst is crucial. Ag and Au electrodes exhibit the best Faradaic efficiencies towards CO.^[71]^ Subsequently, modified electrocatalysts based on Au[^10^, ^11^, ^73^] and Ag[^9^, ^77^] were developed. We opted for a metallic Ag electrode modified by an organic film, *N,N*‐ethylene‐phenanthrolinium dibromide (**1‐Br_2_**), based on the previously reported method by Peters.^[78]^ Upon application of a cathodic potential, **1–Br_2_** is reduced *in‐situ* and forms a film on the Ag electrode, preventing the competitive HER. This catalyst offers numerous benefits, including high selectivity for CO, even under low CO_2_ pressures, ease of assembly, and the ability to easily remove the organic film from the electrode, enabling its reuse (*vide infra*). The reactions were run in a custom‐made three‐chamber reactor (Figure S7 and Graphical Abstract) through which the electrodes were passed. The electrochemical part of the reactor is akin to an H‐cell, with anodic (A) and cathodic (B) chambers separated by a sintered glass. The third compartment (C) constitutes the carbonylation chamber and is connected to the cathodic chamber by a glass bridge to allow CO diffusion. This three‐chamber architecture separates the oxygen evolution reaction (OER) taking place at the anode from the eCO_2_RR and carbonylation reaction. The performance of Ag wire in the presence and absence of the **1‐Br_2_** additive for eCO_2_RR was evaluated in aqueous KHCO_3_ (0.1 M) by chronoamperometry (CA) coupled to gas chromatography (Figure S6). In the presence of 10 mM of **1‐Br_2_** additive in the media CO was selectively produced in aqueous KHCO_3_ solution with CO/H_2_ ratio of 14.4, as determined by gas chromatography, leading to quantitative conversion into **2 a** and equal quantitative yield (Table 2, entry 1). In the absence of **1‐Br_2_**, the silver working electrode mostly generated H_2_ (CO/H_2_=0.05) during CA at −1.0 V *vs* RHE. Consequently, the use of Ag as eCO_2_RR catalyst for tandem carbonylation resulted in full conversion of **1 a**, but only in a low yield of 35 % for **2 a** (Table 2, entry 2). Importantly, it was here identified that the presence of H_2_, even if only small quantities, increased substantially the amount of product derived from hydrodeiodination of **1 a**, namely, production of toluene. This result affirms the outstanding selectivity of the **1‐Br_2_**‐modifiled Ag electrode for eCO_2_RR over HER in aqueous electrolyte. Additional selected optimizations of the eCO_2_RR and the carbonylation reaction are shown in Table 2. For the optimal results (Table 2, entry 1), the carbonylation reaction was started after 15 h of eCO_2_RR, and ran for 5 h using the previously determined optimal conditions (Table 1, entry 1). In the absence of either **1‐Br_2_** additive, TEA, or both, the yields dropped to 35 %, 87 %, and 48 % (Table 2, entries 2–4), respectively. We obtained similar outcome when changing from polycrystalline to nano‐structured Ag electrode (Table 2, entries 4–5). The duration of eCO_2_RR must be sufficient to ensure adequate CO concentration before initiating the carbonylation process. Starting the carbonylation after only 10 h of eCO_2_RR, compared to 15 h, reduces the yield to 80 %, even if eCO_2_RR is continued during the carbonylation reaction to match the same total eCO_2_RR time (15 h) (Table 2, entry 6).

**Table 2:** Selected screening for the eCO_2_RR – carbonylation tandem reaction.

Entry	Deviation from standard conditions^[a]^		Faradaic efficiency [%]		Conv. [%]	Yield **2 a** [%]
	eCO_2_RR	Carbonylation	CO	H_2_		
1	none	none	69.1	4.8	>99	>99
2^[b]^	no **1‐Br_2_**	none	3.7	70.3	>99	35
3^[b]^	none	no TEA	‐	‐	>99	87
4^[b]^	no **1‐Br_2_**	no TEA	‐	‐	>99	48
5^[b]^	nanoAg, no **1‐Br_2_**	no TEA	‐	‐	90	55
6^[c]^	10 h + 5 h	none	‐	‐	90	80
7^[d]^	none	**1 a** (1.0 mmol)	‐	‐	>99	71

[a] Reaction conditions: *eCO_2_RR* – CO_2_‐saturated KHCO_3_ solution (0.1 M, pH 6.8, 55 mL), Ag wire working electrode, **1‐Br_2_** (10 mM), 1.0 V *vs* RHE, 23 °C, 15 h; *Carbonylation* – **1 a** (0.25 mmol), Pd^II^@MIL‐101(Cr)‐NH_2_ (4.6 mol % based on Pd), NaHCO_3_ (5 equiv.), TEA (10 equiv.), 1,4‐dioxane:H_2_O (*v/v*, 1 : 3, 12.5 mL), 23 °C, 5 h. Yields and conversions were determined by ^1^H NMR using 1,3,5‐trimethoxybenzene as an internal standard. [b] **1 a** (0.1 mmol), with eCO_2_RR reaction run for 5 h. [c] eCO_2_RR was carried out for 10 h following by 5 h of carbonylation and eCO_2_RR simultaneously. [d] scale‐up: **1 a** (1.0 mmol), isolated yield is reported. See the Supporting Information, Scheme S5.

**Scheme 3:**
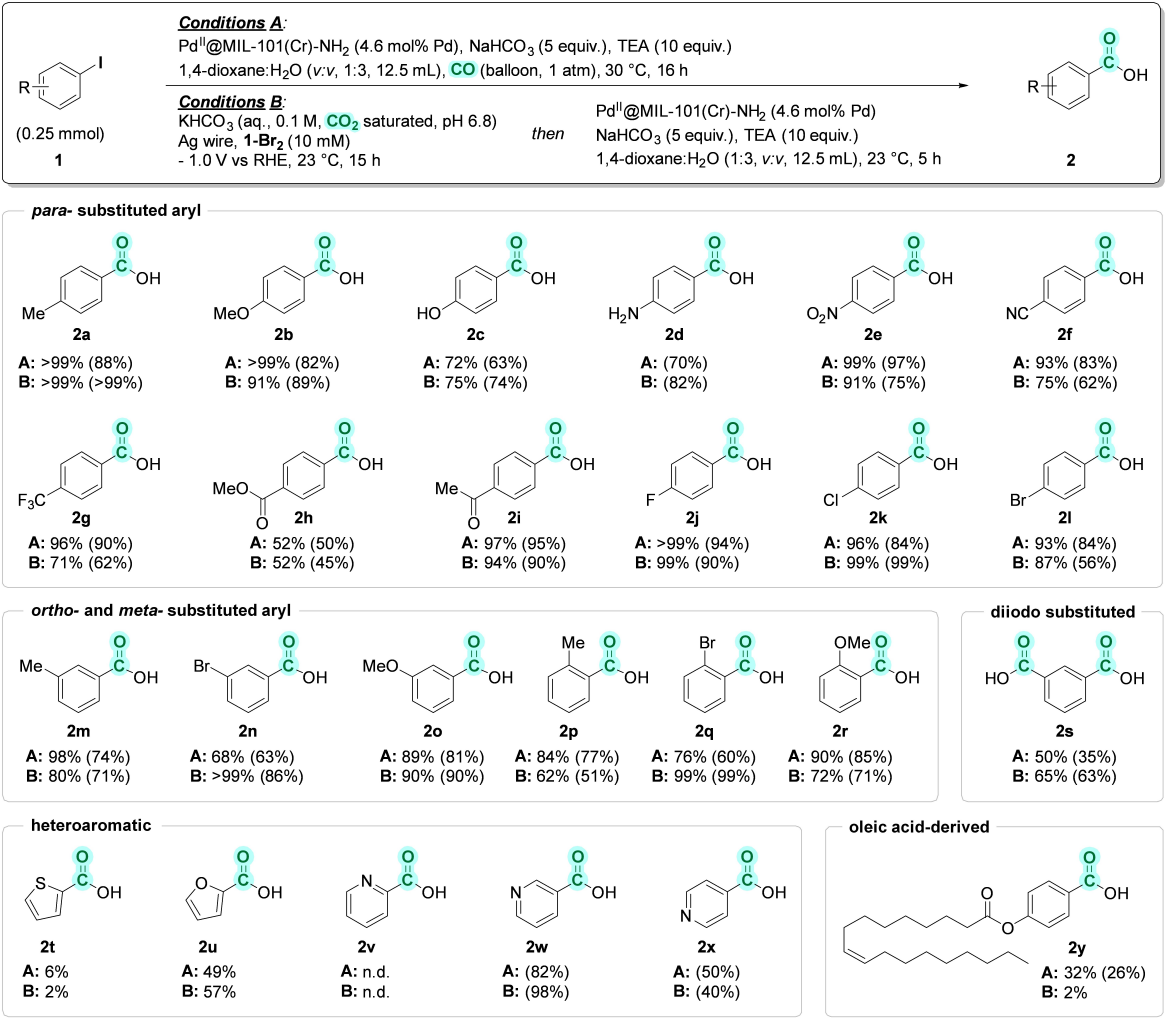
Scope and limitations of the carbonylation reaction under CO atmosphere.[a] Conditions A: as Table 1, entry 1. Conditions B: as Table 2, entry 1. ^1^H NMR yields are given. Isolated yields are in parentheses.

The scope and limitations of the tandem eCO_2_RR – hydroxycarbonylation (Conditions B) were investigated under the optimized conditions (Table 2, entry 1 and Scheme 3) and compared with hydroxycarbonylation using a CO balloon (Conditions A, as Table 1, entry 1). Under Conditions A, substituted benzyl iodide derivatives bearing electron donating groups (EDGs) at the *para*‐position yield the corresponding carboxylic acids **2 a** (*p*‐Me), **2 b** (*p*‐OMe), **2 c** (*p*‐OH), **2 d** (*p*‐NH_2_) in quantitative yields for **2 a** and **2 b**, and in 72 % and 70 % yield for **2 c** and **2 d**, respectively. These results are comparable with those obtained under Conditions B, namely >99 %, 91 %, 75 %, and 82 % for **2 a**, **2 b**, **2 c**, **2 d**, respectively. *para*‐Substituted iodobenzene compounds with different electron withdrawing groups (EWGs), **1 e** (*p*‐NO_2_), **1 f** (*p*‐CN), **1 g** (*p*‐CF_3_), **1 h** (*p*‐COOMe), **1 i** (*p*‐COMe), were also examined. 4‐Nitrobenzoic acid **2 e** was achieved in excellent yields with 99 % and 91 % under Conditions A and B, respectively. A yield of 93 % was obtained for 4‐cyanobenzoic acid **2 f** using CO balloon, and a yield of 75 % under electrochemical conditions. Likewise, 96 % and 71 % yield of 4‐(trifluoromethyl)benzoic acid **2 g** was obtained under Conditions A and B, respectively. Monomethyl terephthalate **2 h** was formed in 52 % yield in both conditions. 4‐Acetylbenzoic acid **2 i** was obtained in near quantitative yields at 97 % and 94 % under CO balloon and electrochemical conditions, respectively. *p*‐F (**1 j**), *p*‐Cl (**1 k**), *p*‐Br (**1 l**) substituted iodobenzenes were well tolerated under both conditions yielding corresponding *p*‐F (**2 j**), *p*‐Cl (**2 k**), *p*‐Br (**2 l**) substituted benzoic acids in excellent yields without interfering with F, Cl, Br substituents. *m*‐Me (**2 m**), *m*‐Br (**2 n**), *m*‐OMe (**2 o**) substituted benzoic acids were obtained in 98 %, 68 %, and 89 % yield under Conditions A, respectively; in comparison with 80 %, >99 %, and 90 % yield under Conditions B, respectively. Under Conditions A, *o*‐Me (**2 p**), *o*‐Br (**2 q**), *o*‐OMe (**2 r**) substituted benzoic acids were formed in 84 %, 76 %, and 90 % yield, respectively; while 62 %, 99 %, and 72 % yield, respectively, was obtained under Conditions B. 1,3‐Diiodobenzene **1 s** was examined under Conditions A yielding isophthalic acid **2 s**, and the incomplete carbonylated product 3‐iodobenzoic acid **2 s’** in 50 % and 30 % yield, respectively. Under Conditions B, 65 % yield of isophthalic acid was obtained. 2‐ Thiophenecarboxylic acid **2 t** was formed in trace amounts what shows the inhibiting effects of sulfur on catalytically active Pd species, while a synthetically useful yield of 2‐furoic acid **2 u** was obtained. This result indicates the inhibiting effects of sulfur on catalytically active Pd species. Synthesis of picolinic acid **2 v** was unsuccessful, while nicotinic acid **2 w** and isonicotinic acid **2 x** were formed in good to excellent yields. Oleyl 4‐iodobenzoate **1 y** was also converted to the corresponding benzoic acid **2 y** in 32 % yield under Conditions A, however, only trace amount of the desired product was obtained under Conditions B.

Differences in yields within a 20 % range between Conditions A and Conditions B underscore the outstanding performance of the Pd^II^@MIL‐101(Cr)‐NH_2_ catalyst, whether utilizing pure CO or CO electrochemically derived from CO_2_.

To gain insights of the oxidation states of Cr and Pd before and after catalysis, X‐ray photoelectron spectroscopy (XPS) analysis was carried out on Pd^II^@MIL‐101(Cr)‐NH_2_. The XPS survey spectrum of the as‐prepared Pd^II^@MIL‐101(Cr)‐NH_2_, namely, before catalysis, reveals the presence of Cr, O, C, Pd, F, N, and Cl, corresponding well with the expected signals for the MOF (Figure S5A). Very similar spectra were obtained after reaction under both conditions A and B, except for the disappearance of the binding energy peak corresponding to Cl when using either condition. This is due to replacement of the chloride ligand on Pd by iodide, which is provided by the substrate, *p*‐iodotoluene (**1 a**), during the oxidative addition step in the catalytic cycle. Indeed, an increase of the binding energy peak corresponding to I is clearly observed in the XPS survey spectra (Figure S5).

**Figure 2:**
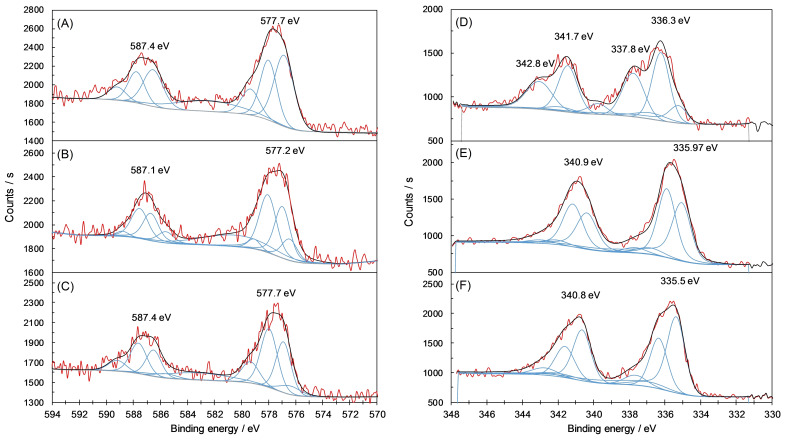
Cr2p (A–C) and Pd3d (D−F) XPS spectra of Pd^II^@MIL‐101(Cr)‐NH_2_ before catalysis (A and D) and after carbonylation using Conditions A (B and E) or Conditions B (C and F).

Figure 2 and Table 3 show the binding energies of Cr and Pd on Pd@MOF before and after catalysis. Figure 2 A shows the XPS Cr2p region for Pd^II^@MIL‐101(Cr)‐NH_2_ before catalysis, with the Cr2p_1/2_ and Cr2p_3/2_ peaks centered at 587.4 eV and 577.7 eV. These signals correspond to a mixture of oxides and hydroxides of Cr^III^, matching well to the reported XPS data for MIL‐101.^[79]^ Very little difference is noticeable in the Cr2p region after carbonylation under both conditions (Figures 2B and C), which indicates a limited impact on the Cr metal oxidation state after catalysis.

**Table 3:** Composition of Pd^II^@MIL‐101(Cr)‐NH_2_ before and after catalysis under Conditions A and Conditions B based on XPS data.

Region	Pd 3d_5/2_			Cr 2p_3/2_		
Resonance	Pd^0^	Pd^II^	Cr^III^ hydroxides		Cr^III^ oxides	
Binding energy (eV)	335.4	336.3/337.8	579.38	578.24	577.11	576.7
Atomic % before catalysis	19.8	55.2/25	16.0	34.7	49.1	0.3
Atomic % after catalysis, Conditions A	49.7	46.1/4.2	5.4	44.1	35.5	15.1
Atomic % after catalysis, Conditions B	61.7	30.8/7.5	16.3	42.1	33.0	8.3

Figures 2D and F show the Pd3d region of Pd^II^@MIL‐101(Cr)‐NH_2_ before and after catalysis. The XPS spectrum of the species before catalysis (Figure 2D) displays peaks corresponding to the Pd3d_3/2_ and Pd3d_5/2_ at respectively 341.7 eV and 336.3 eV, with shoulder peaks at 342.8 eV and 337.8 eV. These peaks were assigned to a mixture of Pd^II^ species with different coordination environments. The as‐prepared catalyst also shows a contribution due to Pd^0^ at 335.4 eV. After carbonylation reaction under either condition (Figures 2E and F), the shoulder peaks decrease sharply, but there still remains an intense contribution due to Pd^II^ species. In addition, the contribution due to Pd^0^ species increases substantially, reflecting the reduction of Pd^II^ under catalysis.

**Figure 3:**
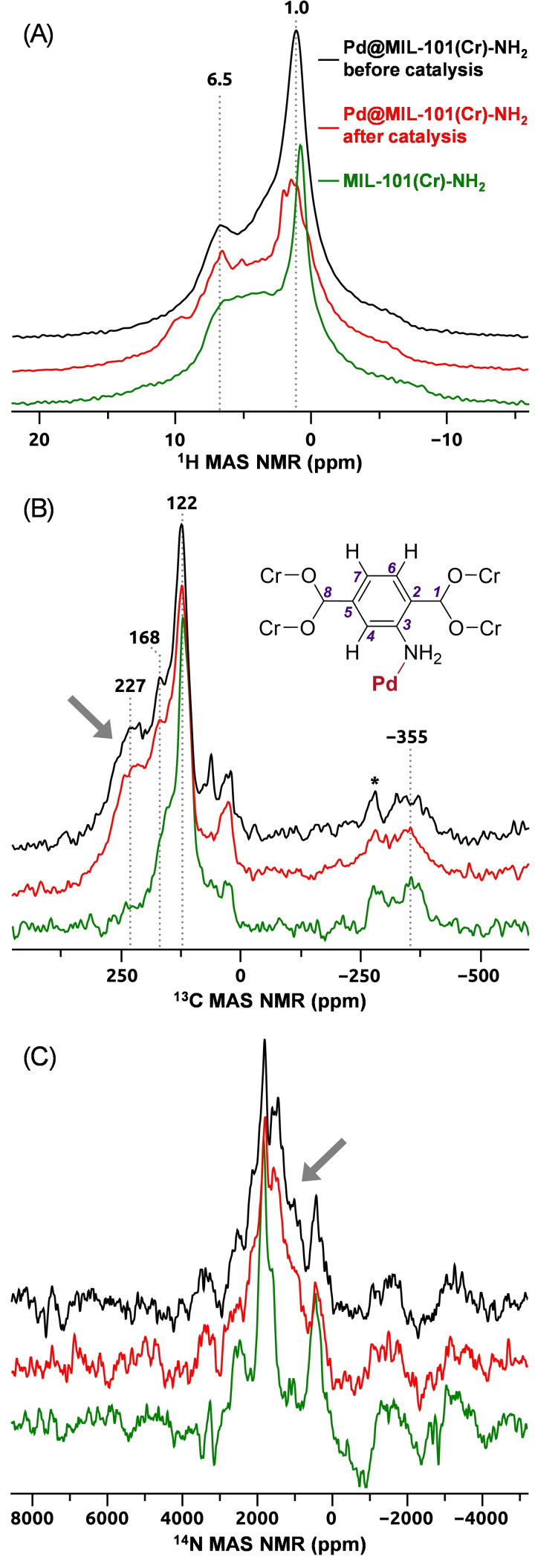
Overlay of the ^1^H (A), ^13^C (B), and ^14^ N (C) MAS NMR spectra of the MIL‐101(Cr)‐NH_2_ (green) and the Pd^II^@MIL‐101(Cr)‐NH_2_ before (black) and after (red) carbonylation under CO atmosphere.

Solid‐state magic‐angle spinning (MAS) NMR spectra of NMR‐active nuclei present in the material (^1^H, ^13^C, and ^14^N) were collected to probe the local structure and examine potential structural changes upon doping of the material with Pd^II^ ions, and to check the sample integrity after catalysis. Note that reports of solid‐state NMR data from paramagnetic catalysts are very uncommon, since acquisitions of such data impose serious experimental challenges due to interactions of NMR‐active nuclei with unpaired electrons, and require very fast MAS rates and high‐power radiofrequency (rf) irradiation to obtain meaningful spectra. Typical cross‐polarization (CPMAS) techniques targeting ^13^C and ^15^N are not feasible anymore due to short coherence lifetimes and rf power (broadbandness) limitations. Therefore, we focused on direct NMR detection of ^1^H, ^13^C and low‐gamma ^14^N isotope (99.6 % nat. abund.) under very fast MAS conditions. MAS NMR spectra acquired from the Pd^II^@MIL‐101(Cr)‐NH_2_ samples before and after catalysis (Conditions A) were compared with those recorded from the MIL‐101(Cr)‐NH_2_ sample, and data are shown in Figure 3.

The ^1^H MAS NMR spectra (Figure 3A) of both MIL‐101(Cr)‐NH_2_ (green) and Pd@MIL‐101(Cr)‐NH_2_ before catalysis (black) are similar, showing signals at 1.0 ppm, corresponding to the bound H_2_O or OH^−^ groups on the Cr cluster, and at 6.5 ppm corresponding to the aromatic hydrogen on the 2‐ATA linker. Signal from the ‐NH_2_ group is expected between 3 and 4 ppm, and it is visible as a shoulder between those at 1 and 6.5 ppm. The ^1^H MAS NMR spectrum of Pd@MIL‐101(Cr)‐NH_2_ after catalysis (red) shows a splitting of the signal at 1 ppm, which can be due to ligand substitution reactions occurring at the Cr clusters during the basic catalytic conditions. In addition, a peak around 10 ppm is also seen, which is most likely from traces of toluic acid (**2 a**) in the material after catalysis.

The ^13^C MAS NMR spectrum of MIL‐101 (without the NH_2_ group) was reported by Wittman and shows a single signal at 130 ppm for the four identical aromatic C− H carbons.^[86]^ For the MOFs used here, due to the presence of the NH_2_ group, two peaks are found in this region at 122 and 168 ppm (Figure 3B). The peak at 122 ppm is attributed to the three C−H carbons at C4, C6 and C7, and the one at 168 ppm to C3 of the 2‐ATA linker. In addition, there is a peak at around −355 ppm that is affected by paramagnetic NMR shift interactions with Cr ions. This signal was also observed by Wittman and co‐workers for the ^13^C MAS NMR spectrum of MIL‐101(Cr),^[86]^ and was attributed to ipso‐carbons (C2 and C5) or to the carboxylic carbons (C1 or C8) of the 2‐ATA linkers. In the MOFs samples containing Pd (black and red, Figure 3B), a broad, distinct signal at 227 ppm is also observed (gray arrow). This signal can be related to the presence of the palladium in the material, since it is absent in the MIL‐101(Cr)‐NH_2_ sample. Importantly, this signal is observed in the ^13^C MAS NMR spectra of the samples before and after catalysis, which suggests no significant changes to the material after catalysis.

The same conclusions can be drawn from the ^14^ N MAS NMR spectra shown in Figure 3C. The ^14^ N spectra of the Pd^II^@MIL‐101(Cr)‐NH_2_ samples before and after catalysis are essentially identical, indicating the structural robustness after one catalytic run, in agreement with the XPS analyses. A distinct spectral feature (marked with gray arrow in Figure 3C) is visible in the ^14^ N MAS NMR spectra of Pd^II^@MIL‐101(Cr)‐NH_2_ samples before and after catalysis, which is absent for the MIL‐101(Cr)‐NH_2_ sample. This feature can be related to the presence of palladium in the material, in agreement with the ^13^C MAS NMR spectra of Figure 3B.

**Figure 4:**
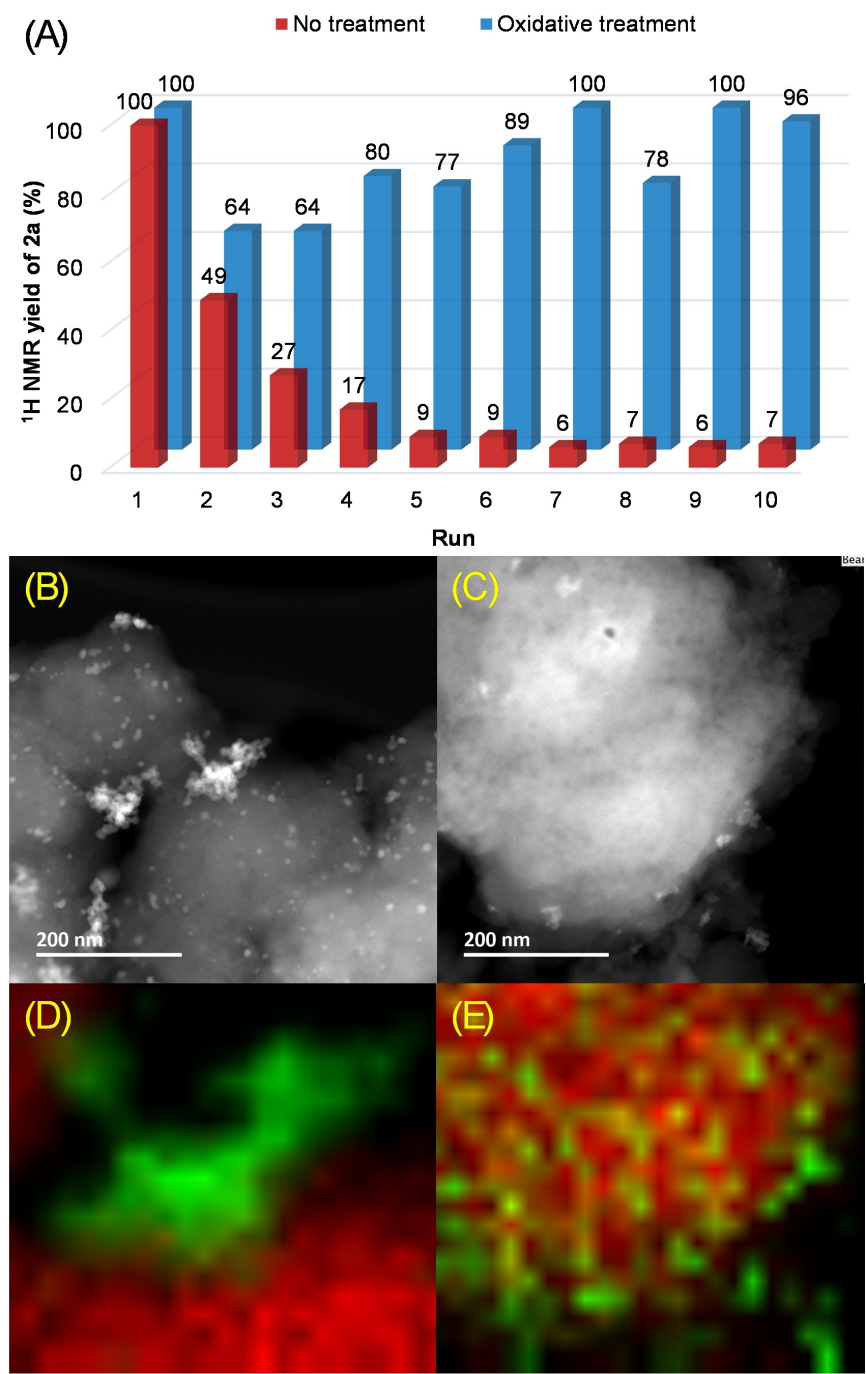
Recyclability test of Pd^II^@MIL‐101(Cr)‐NH_2_ under Conditions A. Carbonylation of 1a using recycled Pd^II^@MIL‐101(Cr)‐NH_2_ for 10 consecutive runs (a). HAADF‐STEM images of recycled Pd^II^@MIL‐101(Cr)‐NH_2_ after 10 consecutive runs following No treatment method (b) and Oxidative treatment method (c). TEM‐EDS mapping images of recycled Pd^II^@MIL‐101(Cr)‐NH_2_ after 10 consecutive runs following method A (d) and method B (e), the green and red colors depict Pd and Cr, respectively.

The recyclability studies of both surface‐modified Ag electrode and Pd^II^@MIL‐101(Cr)‐NH_2_ heterogeneous catalyst were carried out. For the carbonylation under CO balloon conditions, the first attempts to reuse Pd^II^@MIL‐101(Cr)‐NH_2_ were unsuccessful (Figure 4A, No treatment). The heterogeneous catalyst recycled by simply rinsing with aqueous 1,4‐dioxane solution showed a gradual decrease in reaction yields. After 4 recycling runs, a neglectable amount of the desired product was obtained. The significant loss in catalytic performance of Pd^II^@MIL‐101(Cr)‐NH_2_ was due to formation of Pd^0^ clusters (Figures 4B and D). Hence, we introduced an oxidative treatment step of the catalyst in the recyclability experiment (Figure 4A, Oxidative treatment).^[87]^ This recycling method consists of treatment with K_2_S_2_O_4_ aqueous solution at 25 °C for 2 h to regenerate Pd^II^ species dispersed into MIL‐101(Cr)‐NH_2_ (Figures 4C and E), re‐establishing the catalytic activity to a good extent even after the 10^th^ run. PXRD patterns of the recycled catalysts after 1 run and after 10 runs did not show the same reflection peaks as the pristine sample (Figure S12), indicating lack of long‐range periodic order. This amorphization of MOFs, *i. e*. maintaining structural building blocks and its connectivity while lacking long‐range order, is likely caused by disorder in the MOF crystal lattice. This amorphization has been observed for MOFs catalysts,^[88]^ and was reviewed by Cheetham and co‐workers, where the broad reflection peaks originate from diffuse scattering.^[89]^ However, the performance of the catalyst following oxidative treatment was preserved.

**Figure 5:**
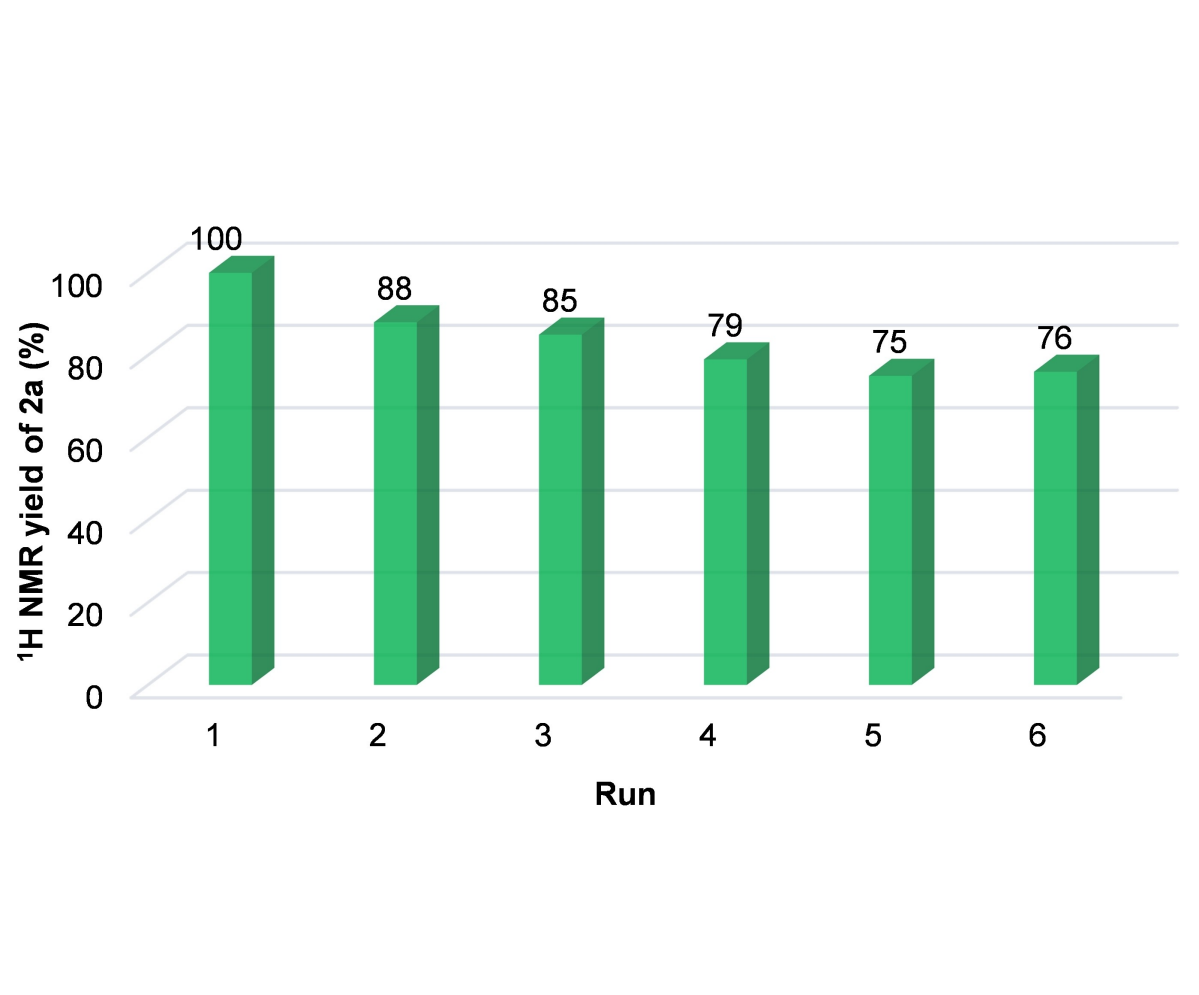
Recyclability test of the **1‐Br_2_** @Ag electrode.

We also performed recycling experiments of the **1‐Br_2_** @Ag catalyst.[^78^, ^90^] An initial run was performed under standard conditions as defined in Table 2 (entry 1), with a fresh silver electrode in the presence of 10 mM of **1‐Br_2_** additive. For the subsequent runs, the electrode was rinsed with deionized water and re‐used in a fresh electrolyte solution (KHCO_3_ aq., 0.1 M) without additional **1‐Br_2_**. After the initial run, the silver wire took a coppery orange hue, indicating the functionalization of the Ag surface by the reduced **1‐Br_2_**. SEM micrographs further confirm the presence of rough coating on the surface of the silver wire, and energy dispersive X‐ray spectroscopy (EDS) analysis of the coating reveals a high content of carbon, coherent with the structure of **1‐Br_2_** (Figure S13). Figure 5 displays the yields obtained for the six carbonylation reactions (Conditions A) performed with the same **1‐Br_2_** @Ag catalyst. After the first run, the yield drops to 88 % on run 2, and then stabilizes around 75 % after run 4.

## Conclusions

In summary, we have developed a method for the synthesis of benzoic acid derivatives at room temperature from aryl iodides and CO_2_ as CO source. This two‐step approach consisted in an electrocatalytic reduction of CO_2_‐to‐CO, carried out by a polycrystalline silver catalyst and a HER suppressing additive (**1–Br_2_** @Ag), whereas the carbonylation was undertaken by Pd^II^@MIL‐101(Cr)‐NH_2_ heterogeneous catalyst. Using a custom‐made three‐chamber cell, we obtained a library of benzoic acids with moderate to excellent yields, with a broad scope and, importantly, with an excellent correlation between conversions and yields. The stability of Pd^II^@MIL‐101(Cr)‐NH_2_ was investigated using SEM, TEM, EDS, PXRD, XPS and multinuclear (^1^H, ^13^C, ^14^N) MAS NMR, pointing to an excellent stability after the carbonylation reactions under both, CO atmosphere and tandem electrocatalysis conditions. Furthermore, we reported here for the first time the ^14^N MAS NMR spectra of a paramagnetic MOF (Figure 3C). From a *Green Chemistry* perspective, our system allows the use of recyclable catalysts. The active heterogeneous catalyst can be regenerated and reused up to 10 times while keeping its efficiency. Analysis of the recovered catalyst after one hydroxycarbonylation reaction, along with the leaching test results, suggests that there is minimal leach of Pd active species. This outcome highlights the exceptional properties of MIL‐101(Cr)‐NH_2_ as a Pd support and scavenger. The electrode, **1–Br_2_** @Ag catalyst retained 75 % of its efficiency after 6 runs with no required treatment.

## Experimental Section

### Hydroxycarbonylation of Aryl Halides Using CO Balloon

To a 20‐mL vial, aryl iodides **1** (0.25 mmol), Pd^II^@MIL‐101(Cr)‐NH_2_ (15.2 mg, 4.6 mol % Pd loading) and NaHCO_3_ (105.0 mg, 1.25 mmol, 5 equiv.) were added. The reaction vial was then sealed with a rubber septum and purged with CO 5 times before being connected to a CO balloon. A solution of 1,4‐dioxane (3.1 mL), water (9.4 mL) and triethylamine (0.35 mL, 2.5 mmol, 10 equiv.) was then subjected to the reaction vial using a syringe. The reaction was carried out for 16 h at 30 °C. After that, the reaction was stopped by removal of CO balloon and addition of HCl 4 M (4 mL). The mixture was then extracted with CH_2_Cl_2_ (10 mL×5 times) followed by dehydration with anhyd. MgSO_4_ and solvent removal under reduced pressure. Reaction outcome was checked by ^1^H NMR using 1,3,5‐trimethoxybenzene (16.8 mg, 0.1 mmol) was an internal standard. The products were isolated by flash chromatography using n‐pentane: ethyl acetate 90 : 10–30 : 70 solutions as eluents.

### eCO_2_RR – Hydroxycarbonylation Tandem Catalysis

eCO_2_RR ‐ hydroxycarbonylation reaction was run in a custom‐made 3‐chambers cell (Figure S7). The CO_2_RR was carried out using a 3‐electrode setup connected to a Biologic SP150 or GAMRY potentiostat. The working and reference electrodes were polycrystalline silver wire (7 cm^2^) and Ag/AgCl (3.0 M KCl) respectively, counter electrode was a Pt wire (8 cm^2^). Chambers A and B of the reactor were filled with CO_2_‐saturated 0.1 M KHCO_3_ electrolyte (55 mL, pH=6.8), and 10 mM of **1–Br_2_** additive were added in the catholyte. In chamber C, 4‐iodotoluene (54.5 mg, 0.25 mmol, 1 equiv.), Pd^II^@MIL‐101‐NH_2_ (15.2 mg, 4.6 mol % Pd loading) and NaHCO_3_ (105.0 mg, 1.25 mmol, 5 equiv.) were added. Electrodes were mounted and the system was sealed under CO_2_ atmosphere. Chronoamperometry was carried out at −1.0 V *vs* RHE under stirring for 15 h, unless stated otherwise. After 15 h, triethylamine (350 μL, 2.5 mmol, 10 equiv.) and 12.5 mL of 1,4‐dioxane:water (1 : 3) were added to chamber C, the resulting suspension turned from green to black quickly and was stirred for 5 h. The eCO_2_RR and hydroxycarbonylation were carried out at 23 °C. The hydroxycarbonylation reaction was stopped by addition of HCl 4 M (4 mL). The mixture was then extracted with CH_2_Cl_2_ (10 mL×5 times) followed by dehydration with anhyd. MgSO_4_ and solvent removal under reduced pressure. Reaction outcome was checked by ^1^H NMR using 1,3,5‐trimethoxybenzene (16.8 mg, 0.1 mmol) was an internal standard. The products were isolated by flash chromatography using n‐pentane: ethyl acetate 90 : 10–30 : 70 solutions as eluents.

## Conflict of Interests

The authors declare no conflict of interest.

1

## Supporting information

As a service to our authors and readers, this journal provides supporting information supplied by the authors. Such materials are peer reviewed and may be re‐organized for online delivery, but are not copy‐edited or typeset. Technical support issues arising from supporting information (other than missing files) should be addressed to the authors.

Supporting Information

## Data Availability

The data that support the findings of this study are openly available in Zenodo at https://doi.org/10.5281/zenodo.11233022, reference number 11233021.
