# Supplementary material for: Synthesis of Benzoic Acids from Electrochemically Reduced CO2 Using Heterogeneous Catalysts
Source: ChemSusChem. 2024 Nov 5;18(3):e202401084. doi: 10.1002/cssc.202401084 (PMC11790006; doi:10.1002/cssc.202401084)
Supplement: Supplementary file 1 — Supporting Information [file CSSC-18-e202401084-s001.pdf]

ChemSusChem

Supporting Information

Synthesis of Benzoic Acids from Electrochemically Reduced CO_2 Using Heterogeneous Catalysts

Ha Phan, Robin Gueret, Pablo Martínez-Pardo, Alejandro Valiente, Aleksander Jaworski, Adam Slabon,* and Belén Martín-Matute*

SUPPORTING INFORMATION

Synthesis of Benzoic Acids from Electrochemically Reduced CO_2 Using Heterogeneous Catalysts

Ha Phan†,[a] Robin Gueret†,[b] Pablo Martínez-Pardo,[a] Alejandro Valiente,[a] Aleksander Jaworski,[b] Adam Slabon*,[b, c] Belén Martín-Matute*[a]

[a] Department of Organic Chemistry, Stockholm University, 106 91 Stockholm, Sweden.

[b] Department of Materials and Environmental Chemistry, Stockholm University, 106 91 Stockholm, Sweden.

[c] Faculty of Mathematics and Natural Sciences, Chair of Inorganic Chemistry, University of Wuppertal, 422 19 Wuppertal, Germany.

† Equal contributions.

e-mail: belen.martin.matute@su.se

e-mail: slabon@uni-wuppertal.de

Number of pages: 64

Number of figures: 66

Number of tables: 2

Number of schemes: 5

Contents

1. **General considerations** **2**
2. **Synthesis of catalysts** **3**
   2.1. Synthesis of 1-Br_2 3
   2.2. Synthesis of MIL-101(Cr)-NH_2 3
   2.3. Synthesis of Pd^{II}@MIL-101(Cr)-NH_2 and Pd^0@MIL-101(Cr)-NH_2 4
3. **Catalyst characterizations** **5**
   3.1. Powder X-ray diffraction 5
   3.2. Nitrogen adsorption-desorption analysis 5
   3.3. Thermogravimetric analysis (TGA) 7
   3.4. Scanning electron microscopy (SEM) 7
   3.5. X-ray Photoelectron Spectroscopy (XPS) 8
   3.6. Solid state MAS NMR experiments 10
   3.7. Faradaic efficiency calculation of the 1–Br_2@Ag catalyst 10
   3.8. Faradaic efficiency calculation of the tandem catalysis 11
4. **Carbonylation reactions** **12**
   4.1. Hydroxycarbonylation of aryl iodides using CO balloon 12
   4.2. Kinetic test and leaching test 13
   4.3. Hydroxycarbonylation of aryl iodides using electrochemically generated CO from CO_2 (tandem catalysis) 13
5. **Recyclability studies** **15**
   5.1. Pd^{II}@MIL-101(Cr)-NH_2 recyclability 15
   5.2. Recycling of the 1–Br_2@Ag catalyst. 19
6. **Scale-up experiment** **21**
7. **Scope of the reactions** **22**
8. **NMR spectra** **37**
9. **References** **63**

1. General considerations

General. Electrochemical experiments were performed on a Biologic SP150 and GAMRY potentiostats, Ag/AgCl electrode was used as a reference electrode, Pt wire was used as anode. The three-chamber electrochemical cells are home made. NMR spectra (^{1}H, ^{13}C, ^{19}F) were acquired on a Bruker Avance 400 MHz spectrometer. Chemical shifts (δ) are reported in ppm relative to residual solvent signals ($CDCl_3$, δ_H = 7.26 ppm, δ_C = 77.16 ppm; or CD_3OD, δ_H = 3.31 ppm, δ_C = 49.00 ppm). ^{13}C NMR and ^{19}F spectra were acquired on a broad band decoupled mode. The following abbreviations are used to describe peak patterns when appropriate: s (singlet), d (doublet), t (triplet), q (quartet), quint (quintet), m (multiplet), br (broad). Reactions were monitored by ^{1}H NMR, and/or TLC on silica gel plates (60 Å porosity, 250 μm thickness). Analytical thin layer chromatography (TLC) was performed using pre-coated aluminum-backed plates (Merck Kieselgel 60 F_{254}) and visualized using potassium permanganate stain, and/or UV light with wavelength of 254 nm. Flash column chromatography was performed using silica gel Merck-60 from Aldrich. Elemental analysis was performed by duplicate on a Carlo Erba Flash 1112 elemental analyzer and the metal content was determined by inductively coupled plasma-optical emission spectrometry (ICP-OES) on a Varian Vista MPX ICP-OES at Medac Ltd, Chobham, UK. Solid-state MAS NMR experiments were performed on a Bruker Avance-III spectrometer. SEM images were acquired using a JEOL-7401F and JEOL-7000F field-emission scanning electron microscope at 5.0 kV. SEM/EDS experiments were done with acceleration voltage of 15 kV. The samples were loaded on carbon ink or on a TEM grid by drop-casting using EtOH solvent prior to SEM analysis. TEM experiments were done on Schottky-type field emission gun-equipped JEOL JEM-2100F at the acceleration voltage of 200 kV. Thermalgravimetric analysis (TGA) was performed under a nitrogen flow between the temperature range of 25 °C and 600 °C and heating rate of 4 $°C.min^{-1}$ using TA Instruments Discovery thermogravimetric analyzer in an aluminum cup loaded with ca. 7 mg of samples. Nitrogen absorption analysis data were obtained at 77 K on a Micromeritics ASAP2020 analyzer with the pressure range p/p^0 = 0.001 - 0.98. The gas absorption analysis samples were degassed at 150 °C for 10 h under 10 μmHg vacuum prior to analysis. Powder X-ray Diffraction (PXRD) data of the MOF samples were acquired using a Panalytical X'pert Pro diffractometer (Cu $K\alpha_{1,2}$, λ_1 = 1.5406 Å, λ_2 = 1.5444 Å). To estimate the nanoparticle size with PXRD using Scherrer's equation Cu $K\alpha_1$ was used. Centrifugation was performed in a Centurion Scientific K3 series centrifuge. Microwave reactions were performed in an Initiator Classic microwave reactor from Biotage®.

Chemicals and gases. Silver wire (99.9%) purchased from ABCR was polished with SiC sandpaper and cleaned by ultrasonication 5 min in ethanol, then D.I. water. 1,2 dibromoethane (98%) was purchased form Alfa Aesar and 1,10-phenanthroline (>98%) from TCI. Chromium (III) chloride ($CrCl_3$, 99%), 2-aminoterephthalic acid (2-ATPA, 99%), sodium fluoride (NaF, 99%) were acquired from Sigma-Aldrich. CO_2 gas was purchased from Strandmøllen with technical grade purity. The technical grade CO gas was purchased from Air Products. Aryl iodide starting materials were obtained from Sigma-Aldrich, TCI and Acros. Reagents were used as received unless otherwise noted.

All original code has been deposited at Zenodo (https://doi.org/10.5281/zenodo.11233022) and is publicly available as of the date of publication.

2. Synthesis of catalysts

2.1. Synthesis of 1-Br₂

Synthesis of the additive *N,N'*-ethylene-phenanthrolinium dibromide (**1-Br_2**) is adapted from Peters.[1]

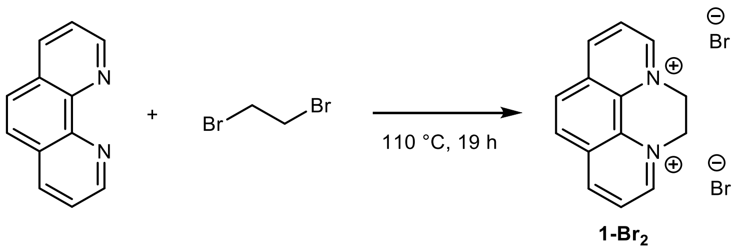

Scheme S1. Synthesis of **1-Br_2**.

Briefly, in a 250 mL round bottomed flask, 1,10-phenanthroline (5 g, 27.7 mmol) was dissolved in 1,2 dibromoethane (50 mL, 577.6 mmol). The reaction mixture is then stirred at 110 °C for 19 h (Scheme S1), the formed precipitate was filtered and washed with Et_2O and acetone to yield **1-Br_2** (9.93 g; 97% yield).

^{1}H NMR (400 MHz, Deuterium Oxide) δ 9.67 (dd, *J* = 5.8, 1.3 Hz, 1H), 9.56 (dd, *J* = 8.5, 1.3 Hz, 1H), 8.73 (s, 1H), 8.64 (dd, *J* = 8.5, 5.8 Hz, 1H), 5.73 (s, 2H).

^{13}C NMR (101 MHz, Deuterium Oxide) δ 148.63, 148.21, 131.89, 129.91, 128.98, 127.58, 52.30.

2.2. Synthesis of MIL-101(Cr)-NH₂

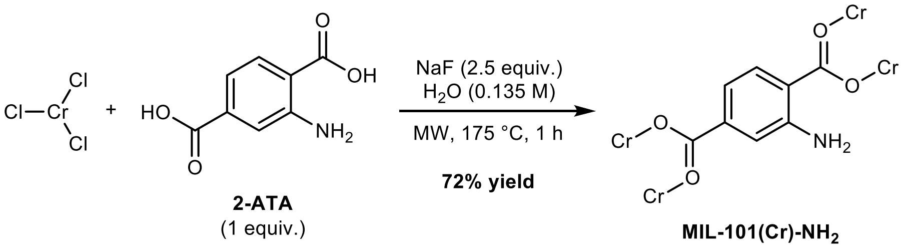

Scheme S2. Schematic representation of the microwave-assisted synthesis of MIL-101(Cr)-NH_2.

The synthesis of MIL-101(Cr)-NH_2 was adapted from Martín-Matute.[2] To a 20-mL Biotage® microwave vial, 2-aminoterephthalic acid (244 mg, 1.35 mmol, 1 equiv.), anhyd. $CrCl_3$ (360 mg, 1.35 mmol, 1 equiv.), NaF (141 mg, 3.35 mmol, 2.5 equiv.) and D.I. water (10 mL) was added. The vial was sealed by a Teflon-lined cap and the mixture was dissolved by sonication for 5 min. Then, the solution was irradiated in a microwave reactor at 175 °C for 1 h (Scheme S2). After reaction, the green crystals were collected by centrifugation and washed with water (30 mL×3 times) then with EtOH (30 mL×3 times) to remove the unreacted substrates. The solid was then activated at 60 °C under vacuum overnight. 248 mg MIL-101(Cr)-NH_2 was obtained, corresponding to 72% yield.

2.3. *Synthesis of Pd^{II}@MIL-101(Cr)-NH_2 and Pd^{0}@MIL-101(Cr)-NH_2*

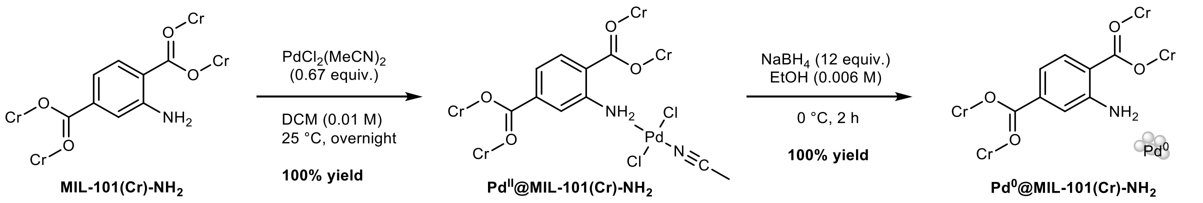

Scheme S3. Synthesis of Pd^{II}@MIL-101(Cr)-NH_2 and Pd^{0}@MIL-101(Cr)-NH_2.

The synthesis of 8 wt% Pd Pd^{II}@MIL-101(Cr)-NH_2, Pd^{0}@MIL-101(Cr)-NH_2 samples were referred to a reported procedure by Martín-Matute (Scheme S3).[2]

Activated MIL-101(Cr)-NH_2 (240 mg, 0.315 mmol) and $PdCl_2(MeCN)_2$ (55 mg, 0.211 mmol) were added to a 50-mL round-bottom flask containing anhyd. DCM (30 mL). The mixture was then stirred overnight at room temperature. Afterwards, the supernatant was removed by centrifugation and the solid was washed with anhyd. DCM (10 mL×2 times) and dried at 40 °C under vacuum overnight. A dark yellowish-green solid Pd^{II}@MIL-101(Cr)-NH_2 was obtained. Elemental analysis (ICP-OES): Experimental (%) = C, 32.07; H, 3.08; N, 4.45; Cl, 6.02, F, 1.92, Cr, 14.67, Pd, 8.75. Calculated (%): C, 32.44; H, 2.16; N, 4.73; Cl, 5.59, F, 2.14, Cr, 17.55, Pd, 8.38.

Pd^{0}@MIL-101(Cr)-NH_2 was synthesized by reduction of Pd^{II}@MIL-101(Cr)-NH_2 (140 mg) with $NaBH_4$ (76 mg, 2.0 mmol) in EtOH (30 mL) at 0 °C for 2 h. A dark-green solid Pd^{0}@MIL-101(Cr)-NH_2 was obtained.

3. Catalyst characterizations

3.1. Powder X-ray diffraction

PXRD patterns of MIL-101(Cr)-NH_2 characteristic peaks were collected using Cu-Kα radiation on a Panalytical X'Pert PRO diffractometer in a 2θ range of 4°–20° with 0.017° step size. A zero-background silicon wafer holder was used to mount the powder samples prior to analysis.

Estimated Pd nanoparticle mean sizes (Figure S1) are calculated using Scherrer's equation.

$$\tau = \frac{K\lambda}{\beta \cos\theta}$$

Equation S1. Scherrer's equation.

Where:

τ is the mean size of Pd NPs

K is a dimensionless shape factor = 0.9

λ is the X-ray wavelength = 1.5406 Å (Cu-Kα irradiation)

β is the line broadening at the half maximum intensity

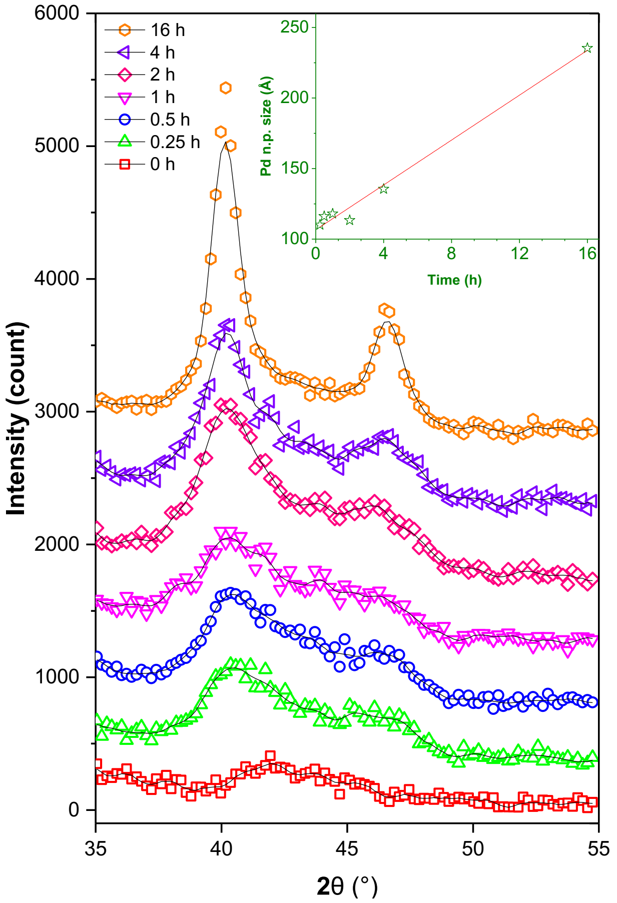

Figure S1. PXRD patterns of Pd@MIL-101(Cr)-NH_2 after catalytic reactions at different reaction time. The insert shows the estimated size of the Pd NPs calculated by Scherrer's equation

3.2. Nitrogen adsorption-desorption analysis

N_2 adsorption analyses were performed with a Micromeritics ASAP 2020 instrument at -196 °C. N_2 adsorption data at relative pressure range of 0.05 – 0.25 were used for determining specific surface areas using Brunauer, Emmet and Teller (BET) model. Porosity distribution was determined by Original Density Functional Theory using N_2 @ 77 K on Carbon, slit pores model. Approx. 60 mg of solid sample was degassed at 10 µmHg and 150 °C prior to analysis.

Nitrogen adsorption-desorption analysis revealed a type-IV isotherm for both MIL-101(Cr)-NH_2 and Pd^{II}@MIL-101(Cr)-NH_2. Relative pressure p/p^0 = 0.03 – 0.15 was used to determine the BET surface area. While MIL-101(Cr)-NH_2 shows surface area of 1777 m^2/g, Pd^{II}@MIL-101(Cr)-NH_2 has a slightly lower surface area of 1620 m^2/g, possibly due to the metalation with the Pd^{II} moiety (Figure S2, A-B).

Pore size distribution shows the similar porosity for both MIL-101(Cr)-NH_2 and Pd^{II}@MIL-101(Cr)-NH_2 ranging from 12 – 30 Å (Figure S2, C-D).

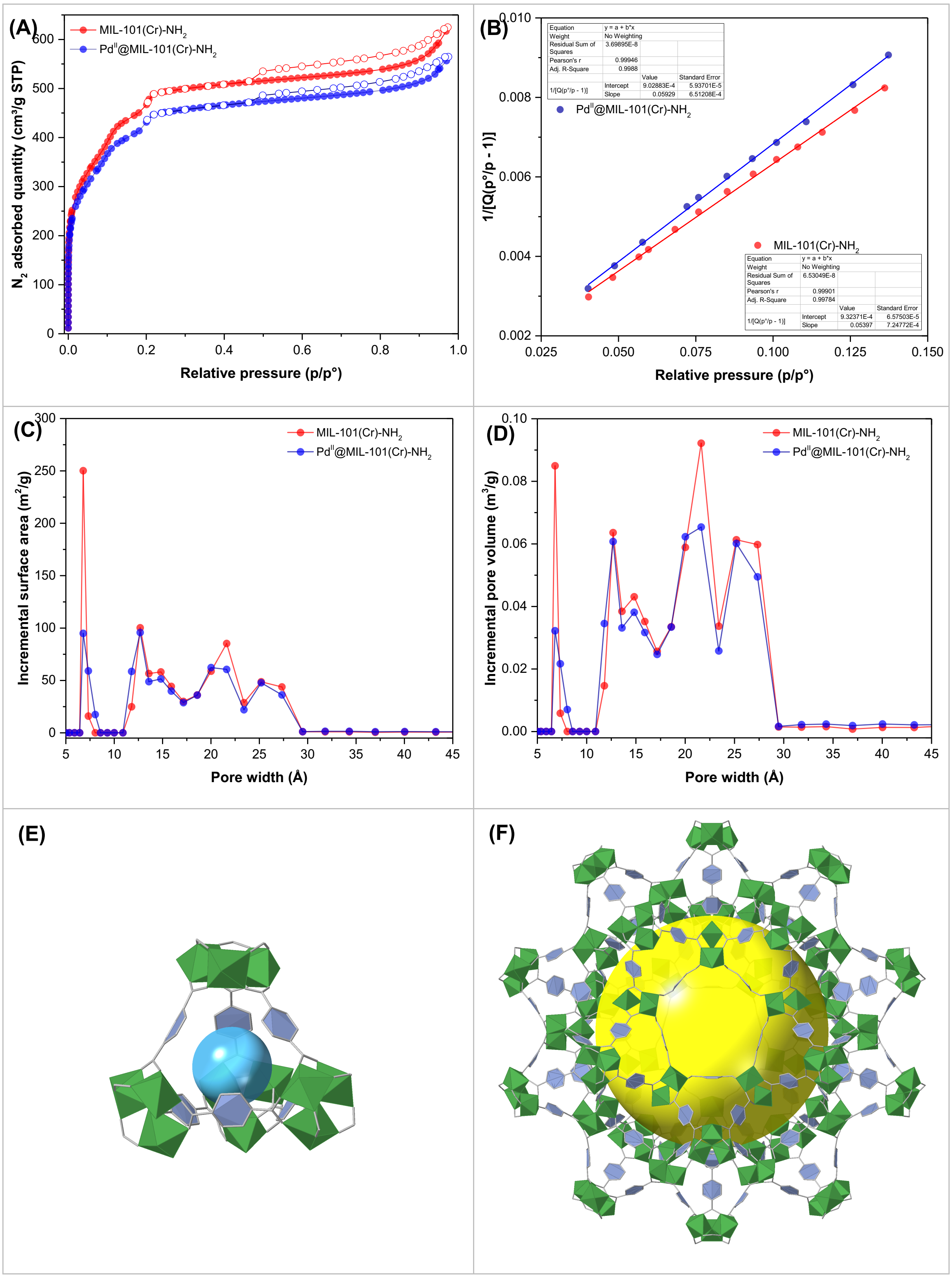

Figure S2. MIL-101(Cr)-NH_2 and Pd^{II}@MIL-101(Cr)-NH_2 characterization. (A) N_2 adsorption – desorption isotherms. (B) Plot of the linear region of the adsorption N_2 isotherm used for BET equation. (C) and (D) porosity distributions calculated by Original Density Functional Theory. Illustration of the tetrahedron pore (E) and main pore (F) of MIL-101(Cr).

3.3. *Thermogravimetric analysis (TGA)*

TGA data were collected on TA Instruments Discovery TG with N_2 flow of 20 mL/min, heat ramping at 5 °C/min from room temperature to 600 °C. Mass spectroscopy analysis of the exhausted fume was done on Pfeiffer Omnistar GSD 320.

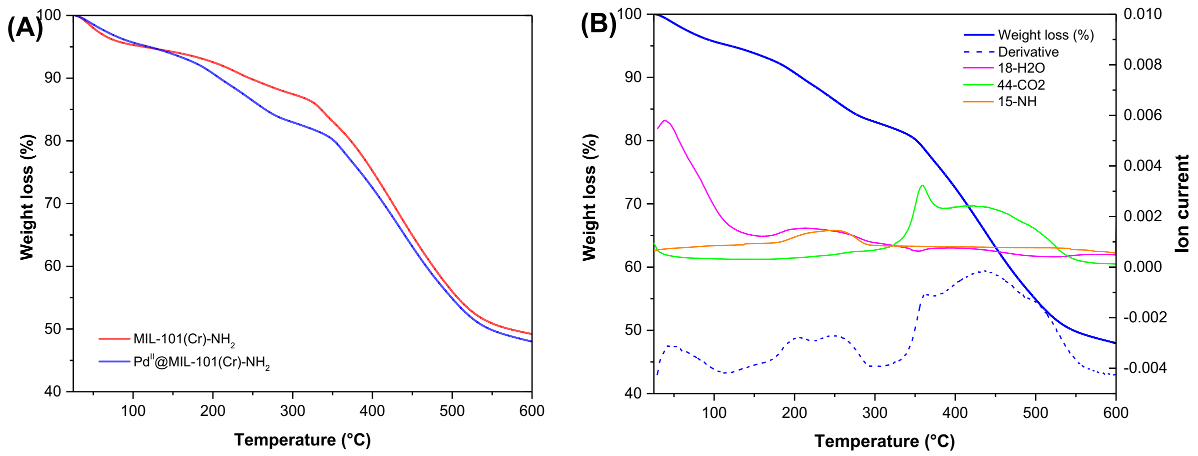

Figure S3. TGA analysis.
(A) TGA curves of MIL-101(Cr)-NH_2 and Pd^{II}@MIL-101(Cr)-NH_2.
(B) MS integrated TGA of Pd^{II}@MIL-101(Cr)-NH_2.

Before 120 °C, moisture uptake by MOF on the surface, from 200 °C to 300 °C, moisture inside the pores and decomposition of the amine group. The structure starts to collapse at 370 °C due to the decomposition of carboxylate bonds (Figure S3).

3.4. *Scanning electron microscopy (SEM)*

SEM images were collected on a JEOL JSM-7000F instrument using a Schottky-type field emission gun at an accelerating voltage of 5.0 kV. Samples were loaded on a TEM grid prior to analysis. Energy-dispersion X-ray (EDS) were collected at an accelerating voltage of 15.0 kV (Figure S4). EDS mapping showed the even distribution of Pd on the MIL-101(Cr)-NH_2 structure.

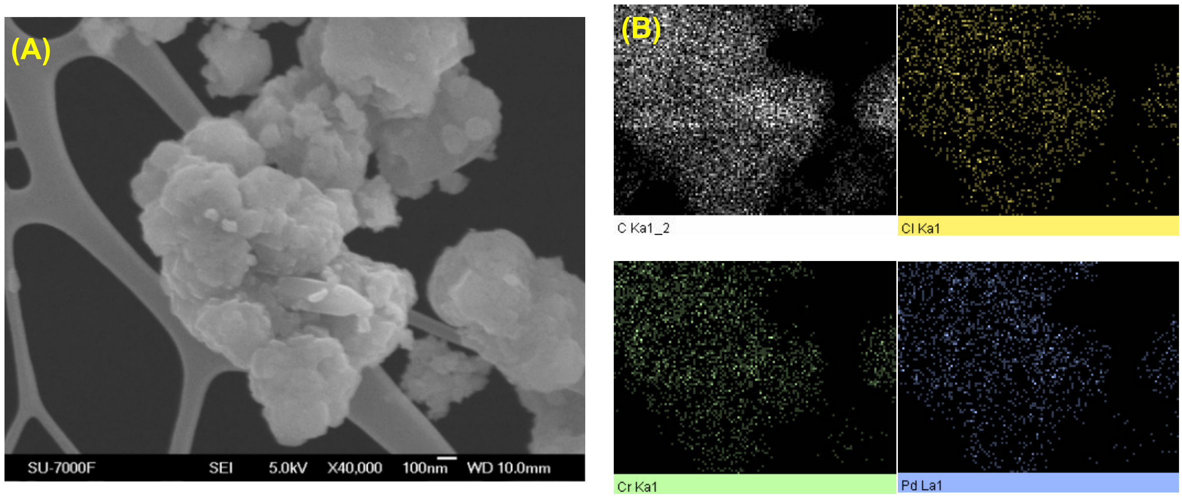

Figure S4. SEM (A) and EDS (B) images of Pd^{II}@MIL-101(Cr)-NH_2.

3.5. *X-ray Photoelectron Spectroscopy (XPS)*

XPS measurements were performed on a Theta Probe (Thermo Fisher Scientific). The X-ray source is a monochromatic Al-Kα source at 1386.6 eV. The spectrometer is calibrated to 368.21 eV binding energy (BE) of the Ag 3d5/2 line for metallic silver and the linearity is corrected to BE of metallic 932.62 eV for the Cu 2p3/2 line and 83.96 eV and Au 4f71/2. Sputtering is performed with an EX05 ion gun with Ar^+ at 3 keV, 1 µA, 4 mm². The lateral resolution of the X-ray spot was set to 400 µm for all measurements. The survey spectra were recorded with a pass energy of 200 eV BE and the detail spectra with a pass energy of 50 eV. The base pressure in the analytical chamber was < $5*10^{-8}$ Pa. Binding states of elements detected were analyzed in reference to the NIST XPS database (NIST, 2012).

Table S1. Composition of Pd^{II}@MIL-101(Cr)-NH_2 before and after catalysis under CO atmosphere or electrochemical conditions based on XPS data.

Region	C1s					O1s					N1s
Resonance	C–C, C–H	C–O	C=O	O–C=O	Aromatic shake-up	Oxides	C–O, O–H	C–O, O–H Pd3p	O–H	Pd3p	–NH_2
Binding energy (eV)	284.5	285.4	288.6	289.5	291.8	530.68	532.02	533.08	534.21	537	399.69
Atomic % unreacted	45.9	37.3	8.7	6.6	1.5	7.9	65.1	17.5	6.8	2.6	100
Atomic % CO	45.6	36.8	11.5	4.1	1.9	10.7	52.01	20.1	8.6	8.3	100
Atomic % EC	45.6	36.8	11.5	4.1	1.9	14.6	60.44	19.4	4.0	1.54	100

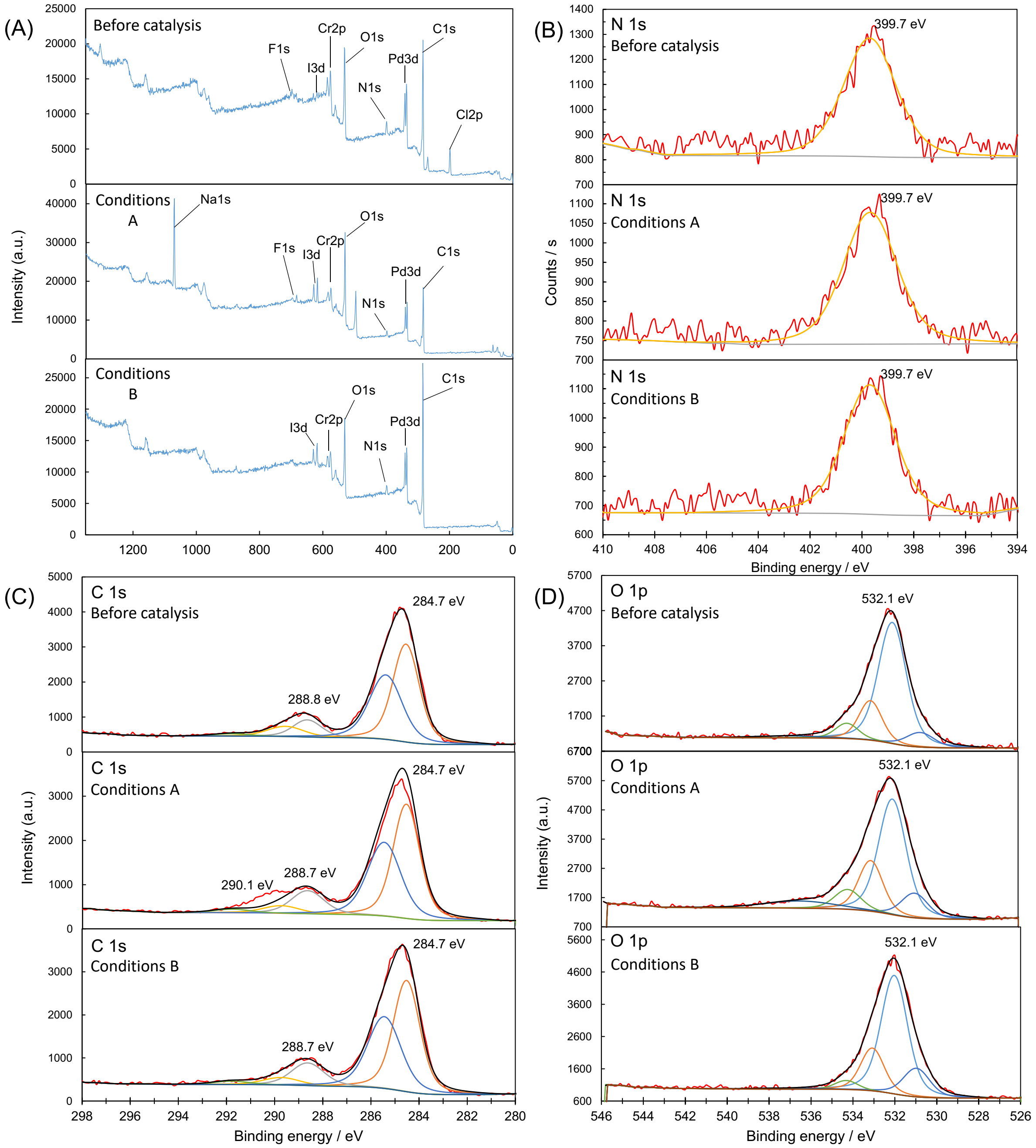

Figure S5. (A) XPS survey spectra and high-resolution XPS spectra of the (B) N1s, (C) C1s and (D) O1p region of the Pd^{II}@MIL-101(Cr)-NH_2 before and after carbonylation reaction under CO atmosphere and tandem catalysis conditions.

3.6. *Solid state MAS NMR experiments*

Magic-angle-spinning (MAS) NMR experiments were performed at a magnetic field of 14.1 T (Larmor frequencies of 600.1, 150.9, 43.4 MHz for ^1H, ^{13}C, and ^{14}N, respectively) on a Bruker Avance-III spectrometer using a 1.3 mm probe head and a 60 kHz MAS rate. The ^1H spectra were involved a use of a rotor-synchronized, double-adiabatic spin-echo sequence with a 90° excitation pulse of 1.25 μs followed by a pair of 50.0 μs tanh/tan short high-power adiabatic pulses (SHAPs) with a 5 MHz frequency sweep. All pulses operated at the nutation frequency of 200 kHz. 128 signal transients were acquired using a relaxation delay of 5 s. The ^{13}C spectra were collected using a spin-echo pulse sequence with a 90 and 180 degree pulse durations of 2.00 and 4.00 μs, respectively. Around 400000 signal scans were collected for each sample with a relaxation time of 1 s. The ^{14}N spectra were collected using a spin-echo pulse sequence with a 90 and 180 degree pulse durations of 3.00 and 6.00 μs, respectively. 262144 signal scans were collected with a relaxation time of 1 s. ^1H and ^{13}C NMR shifts are reported with respect to tetramethylsilane (TMS), and ^{14}N shifts with respect to the solid ammonium chloride (NH_4Cl).

3.7. *Faradaic efficiency calculation of the 1–Br₂@Ag catalyst*

Chronoamperommetry (CA) measurements for Faradaic efficiency calculations were carried out in a H-Cell, Anode and cathode compartments were separated using a PFSA membrane (Fumatech). The electrodes used were a Ag wire (2 cm^2) as working electrode and a Pt wire as counter electrode, potentials were referred versus an Ag/AgCl (3.0 M KCl) reference electrode. CA measurement was carried out for 60 min in 0.1 M $KHCO_3$ (aq.) (pH = 6.8) with a constant CO_2 flow of 45 sccm. Every 15 min, 1 mL of headspace was injected in the sample loop of the GC. The result was averaged over 3 measurements. For each measurement, 500 µL liquid sample were taken and diluted in 500 µL D_2O containing 20 mM DMSO as internal standard, samples were analyzed by ^1H NMR (Bruker) using water suppression technique. Faradaic efficiency was calculated according to the equation below:

$$FE = \frac{zF\mathrm{n}}{i\mathrm{t}}$$

$$FE = \frac{2 \times 96485\ (C/mol) \times nCO(mol)}{V_M(L/mol) \times i\left(\frac{C}{s}\right) \times t(s)} \times 100$$

$$FE = \frac{2 \times 96485 \times vol\% \times 0.001 \times Vf}{24.055 \times i \times 60} \times 100$$

Equation S2. Faradaic efficiency calculation of the 1–Br2@Ag catalyst.

<u>*Where:*</u>

vol% is the fraction of CO in the sample gas (dimensionless).

i is the steady state current of the cell.

Vf corresponds to the volume flow measured by a flow meter (in mL.min^{-1}).

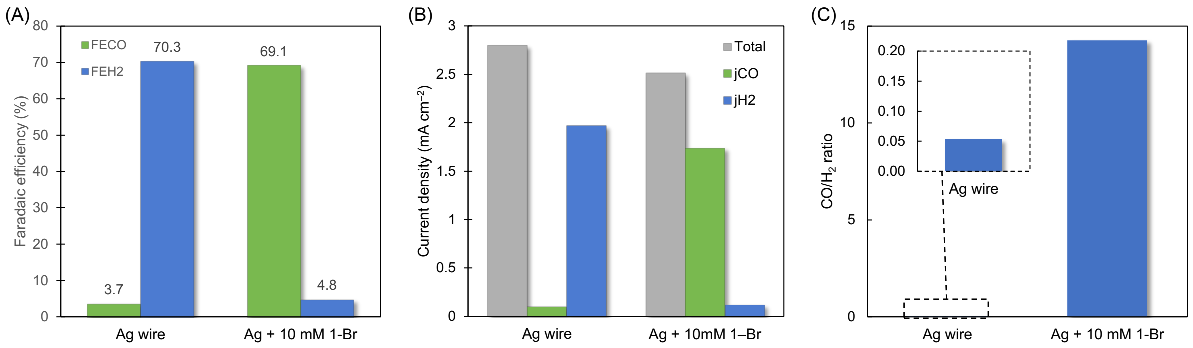

Figure S6. Faradaic efficiency of the Ag and **1-Br$_2$**/Ag system and specific current associated recorded in CO_2 saturated 0.1 M $KHCO_3$ (aq.) at a CO_2 flow of 45 sccm. Addition of **1-Br$_2$** additive totally reverts the selectivity of the system from HER to CO evolution.

At –1.0 V RHE, selectivity of the Ag wire is clearly biased towards H_2 (FE_{H2} = 70 %), and only very little CO is produced (FE_{CO} = 3%), and no formate was detected. However, the selectivity was totally reverted in favor to CO in presence of 10 mM of **1–Br$_2$**, with FE_{CO} = 69% and FE_{H2} = 5%, if the values seem far from those reported by Peters et al. for this system, the trend is very similar.[3] The differences can easily be explained by the differences in setup for the measurements. This strong HER inhibition confirms that this system could work ideally for our tandem catalysis.

3.8. Faradaic efficiency calculation of the tandem catalysis

The Faradaic efficiencies (FEs) for the formation of benzoic acids were calculated based on the amount of isolated product and the charges passed during chronoamperometry experiments (refer to the equation below). For the model reaction using 4-iodotoluene, the isolated yield was 100% under condition B (*i.e.*, tandem reaction), corresponding to a Faradaic efficiency of 21%. Most FE for the various benzoic acid derivatives ranged from 11% to 21%, with only derivatives **2e** and **2u** yielding below 10%. The reported FEs are understandably low because we are using an excess of CO to maximize the benzoic acid yield. Interestingly, when the model reaction was scaled up from 0.25 mmol to 1 mmol, with a longer CA time of 25 h, the yield dropped to 71%, but the Faradaic efficiency increased to 38%. Improved cell engineering and further optimization might allow for high yields to be achieved alongside good FEs

The Fes were calculated using the following formula:

$$FE = \frac{n \times z \times Q}{C} \times 100$$

Equation S3. Faradaic efficiency calculation of the tandem catalysis.

Where n is the amount of isolated product (mol), z the number of electrons required (2 for CO_2 to CO), Q the Faraday constant (C/mol), and C the number of charges (C).

Table S2. Faradaic efficiency with respect to the isolated benzoic acids.

Benzoic acid derivatives	Isolated yield (%)	Charge (C)	FE (%)
**2a**	99	227	21
**2a** (1 mmol scale)	71	364	37.7
**2b**	89	227	18.4
**2c**	74	263	13.6
**2d**	82	230	17.2
**2e**	75	325	11.1
**2f**	62	257	11.6
**2g**	62	252	11.9
**2h**	45	203	10.7
**2i**	90	372	11.7
**2j**	90	236	18.4
**2k**	99	230	20.8
**2l**	56	222	12.2
**2m**	71	260	13.2
**2n**	86	224	18.5
**2o**	90	221	19.6
**2p**	51	243	10.1
**2q**	99	228	21
**2r**	71	201	17
**2s**	63	233	13
**2t**	2	274	0.4
**2u**	57	287	9.6
**2v**	0	275	0
**2w**	98	257	18.4
**2x**	40	220	8.8
**2y**	2	270	0.3

4. Carbonylation reactions

4.1. Hydroxycarbonylation of aryl iodides using CO balloon

To a 20-mL Biotage® vial, aryl iodides (0.25 mmol), Pd^{II}@MIL-101(Cr)-NH_2 (15.2 mg, 4.6 mol% Pd loading) and $NaHCO_3$ (105.0 mg, 1.25 mmol. 5 equiv.) were added. The reaction vial was then sealed with a rubber septum and purged with CO 5 times before being connected to a CO balloon. A solution of 1,4-dioxane (3.1 mL), water (9.4 mL) and triethylamine (0.35 mL, 2.5 mmol, 10 equiv.) was then subjected to the reaction vial using a syringe. The reaction was carried out for 16 h at 30 °C. After that, the reaction was stopped by removal of CO balloon and addition of HCl 4 M (4 mL). The mixture was

then extracted with CH_2Cl_2 (10 mL × 5 times) followed by dehydration with anhyd. $MgSO_4$ and solvent removal under reduced pressure. Reaction outcome was checked by ^{1}H NMR using 1,3,5-trimethoxybenzene (16.8 mg, 1.0 mmol) was an internal standard. The products were isolated by flash chromatography using n-pentane: ethyl acetate 90:10 to 30:70 solutions as eluents.

4.2. Kinetic test and leaching test

Kinetic test: to a 20-mL Biotage® vial, 4-iodotoluene (54.5 mg, 0.25 mmol, 1 equiv.), Pd^{II}@MIL-101(Cr)-NH_2 (15.2 mg, 4.6 mol% Pd loading) and $NaHCO_3$ (105.0 mg, 1.25 mmol, 5 equiv.) were added. The reaction vial was then sealed with a rubber septum and purged with CO 5 times before being connected to a CO balloon. A solution of 1,4-dioxane (3.1 mL), water (9.4 mL) and triethylamine (0.35 mL, 2.5 mmol. 10 equiv.) was then subjected to the reaction vial using a syringe. The reaction was carried out for 0.25 h, 0.5 h, 1.0 h, 2.0 h or 4.0 h at 30 °C. After that, the reaction was stopped by removal of CO balloon and addition of HCl 4 M (4 mL). The mixture was then extracted with DCM (10 mL × 5 times) followed by dehydration with anhyd. $MgSO_4$ and solvent removal under reduced pressure. Reaction outcome was checked by ^{1}H NMR using 1,3,5-trimethoxybenzene (16.8 mg, 1.0 mmol) was an internal standard.

Leaching test: to a 20-mL Biotage® vial, 4-iodotoluene (54.5 mg, 0.25 mmol, 1 equiv.), Pd^{II}@MIL-101(Cr)-NH_2 (15.2 mg, 4.6 mol% Pd loading) and $NaHCO_3$ (105.0 mg, 1.25 mmol, 5 equiv.) were added. The reaction vial was then sealed with a rubber septum and purged with CO 5 times before being connected to a CO balloon. A solution of 1,4-dioxane (3.1 mL), water (9.4 mL) and triethylamine (0.35 mL, 2.5 mmol, 10 equiv.) was then subjected to the reaction vial using a syringe. The reaction was carried out for 0.5 h at 30 °C. Afterwards, the reaction mixture was transferred to an Eppendorf® centrifuge tube and the catalyst was removed by centrifugation at 14000 rpm for 2 min. The supernatant was further passed through a 0.45 µm syringe filter. The filtrate was collected and transferred to a new 20-mL Biotage® vial. The vial was sealed and purged with CO for 5 times before being connected to a CO balloon. The reaction was then carried out for an extra 1.5 h or 3.5 h at 30 °C. After that, the reaction was stopped by removal of CO balloon and addition of HCl 4 M (4 mL). The mixture was then extracted with CH_2Cl_2 (10 mL × 5 times) followed by dehydration with anhyd. $MgSO_4$ and solvent removal under reduced pressure. Reaction outcome was checked by ^{1}H NMR using 1,3,5-trimethoxybenzene (16.8 mg, 1.0 mmol) was an internal standard.

4.3. Hydroxycarbonylation of aryl iodides using electrochemically generated CO from CO_2 (tandem catalysis)

All eCO_2RR - hydroxycarbonylation tandem reactions were run in a custom-made 3-chambers cell (**Figure S7**). The CO_2RR was carried out using a 3-electrode setup connected to a Biologic SP150 or GAMRY potentiostat. The working and reference electrodes were polycrystalline silver wire (7 cm^2) and Ag/AgCl (3.0 M KCl) respectively, counter electrode was a Pt wire (8 cm^2). Chambers A and B of the reactor a) were filled with CO_2-saturated 0.1 M $KHCO_3$ electrolyte (pH = 6.8), and 10 mM of **1–Br$_2$** additive were added in the catholyte. In chamber C, 4-iodotoluene (54.5 mg, 0.25 mmol, 1 equiv.), Pd^{II}@MIL-101-NH_2 (15.2 mg, 4.6 mol% Pd loading) and $NaHCO_3$ (105.0 mg, 1.25 mmol, 5 equiv.) were added. Electrodes were mounted and the system was sealed under CO_2 atmosphere. Chronoamperometry was carried out at –1.0 V vs. RHE under stirring for 15 h, unless stated otherwise. After 15 h, triethylamine (350 µL, 2.5 mmol, 10 equiv.) and 12.5 mL of 1,4-dioxane:water (1:3) were

added to chamber C, the resulting suspension turned from green to black quickly and was stirred for 5 h.

For scale-up reactions, reactor b) was used and chronoamperommetry was carried for 15 h at –1.0 V RHE before starting the carbonylation reaction for 5 h at 23 °C.

After the reaction was completed, the crude of chamber B was taken out and centrifuged at 6000 rpm for 5 min, the supernatant was taken out and the solid was washed with 1,4-dioxane:water (1:3) and centrifuged again. The 2 supernatants were combined, and the solution was brought to pH 1-2 with 1 M HCl. The aqueous solution was extracted three times with EtOAc, dried over $MgSO_4$ and evaporated under vacuum. ^1H NMR yields were calculated by adding 1,3,5-trimethoxybenzene (16.8 mg, 0.1 mmol) as the internal standard. After CA, especially in the longer runs, the silver wire took an orange shade, attributed to the formation of the protective film of reduced **1-Br$_2$** species, as previously reported. The original aspect of the silver wire could be restored after sonication of the wire in DMSO, while the solution took an orange color. The silver wire was then polished with SiC paper (P4000) and rinsed using Milli-Q water before being reused for several runs.

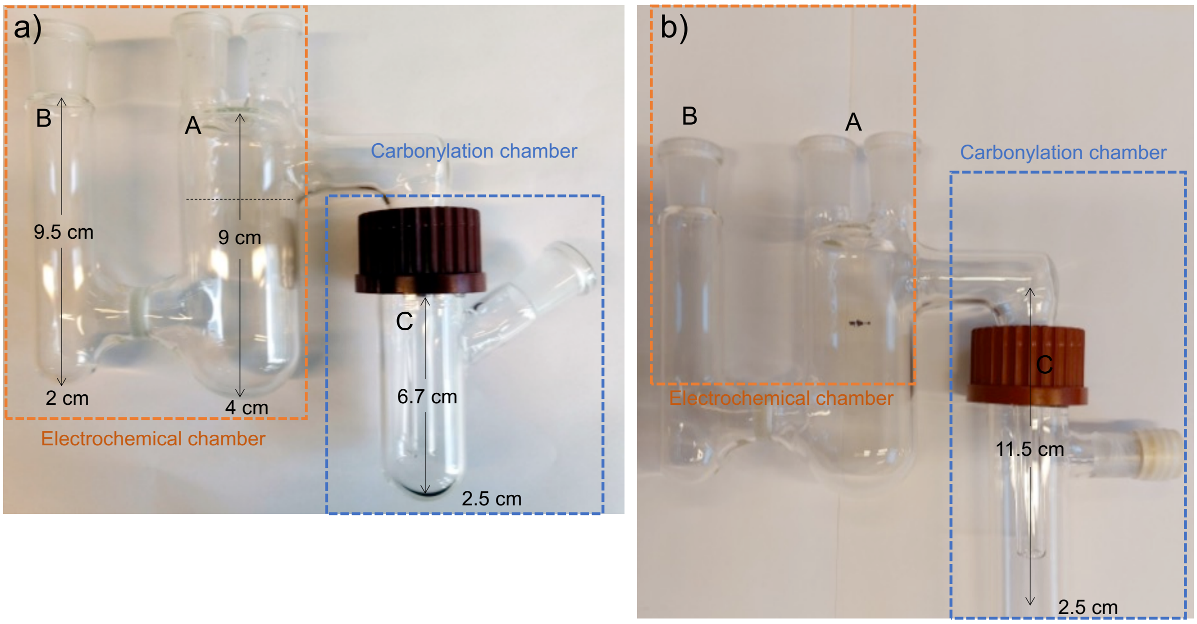

Figure S7. Pictures of the homemade 3-chamber reactors used for the tandem catalysis. Volumes of the different reactors are noted as followed. Reactor a): V_A+V_C = 150 mL, V_B = 31 mL, Reactor b): V_A+V_C = 244 mL, V_B = 59 mL.

5. Recyclability studies

5.1. Pd^{II}@MIL-101(Cr)-NH$_2$ recyclability

No Treatment: After the carbonylation reaction, the catalyst was collected by centrifugation (14000 rpm, 2 min), washed with 1,4-dioxane: water (1:3 *v/v*, 4 mL × 5 times) and subjected to a new reaction batch containing 4-iodotoluene (54.5 mg, 0.25 mmol, 1 equiv.), $NaHCO_3$ (105.0 mg, 5 equiv., 1.25 mmol), triethylamine (350 µL, 2.5 mmol, 10 equiv.) and 1,4-dioxane: water (1:3 v/v, 12.5 mL). The mixture was purged with CO 5 times then the reaction was carried out at 30 °C for 4 h under CO atmosphere. The supernatant and washed solution were collected, following by addition of HCl 4 M (4 mL). The mixture was then extracted with CH_2Cl_2 (10 mL × 5 times) followed by dehydration with anhyd. $MgSO_4$ and solvent removal under reduced pressure. Reaction outcome was checked by ^{1}H NMR using 1,3,5-trimethoxybenzene (16.8 mg, 1.0 mmol) was an internal standard.

Oxidative Treatment: After the carbonylation reaction, HCl 12 M (1.5 mL) was added to the reaction mixture, followed by addition of $K_2S_2O_8$ (101.3 mg, 1.5 equiv., 0.375 mmol). The Pd nanoparticle re-oxidation was carried out at 25 °C for 2 h. Afterwards, the catalyst was collected by centrifugation (14000 rpm, 2 min), washed with 1,4-dioxane: water (1:3 *v/v*, 4 mL × 5 times) and subjected to a new reaction batch containing 4-iodotoluene (54.5 mg, 0.25 mmol, 1 equiv.), $NaHCO_3$ (105.0 mg, 1.25 mmol, 5 equiv.), TEA (350 µL, 2.5 mmol, 10 equiv.) and 1,4-dioxane: water (1:3 *v/v*, 12.5 mL). The mixture was purged with CO 5 times then the reaction was carried out at 30 °C for 4 h under CO atmosphere. The supernatant and washed solution were collected. The mixture was then extracted with CH_2Cl_2 (10 mL × 5 times) followed by dehydration with anhyd. $MgSO_4$ and solvent removal under reduced pressure. Reaction outcome was checked by ^{1}H NMR using 1,3,5-trimethoxybenzene (16.8 mg, 1.0 mmol) was an internal standard.

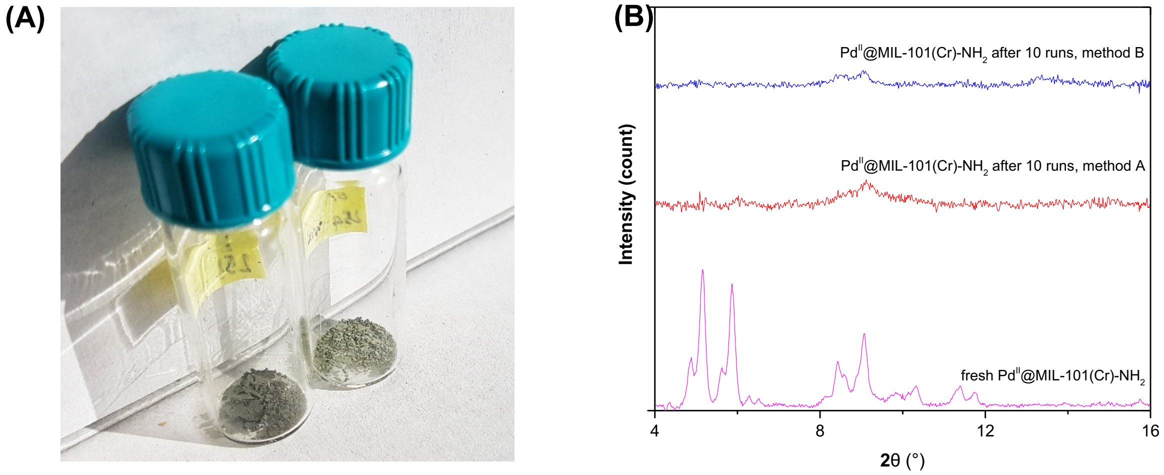

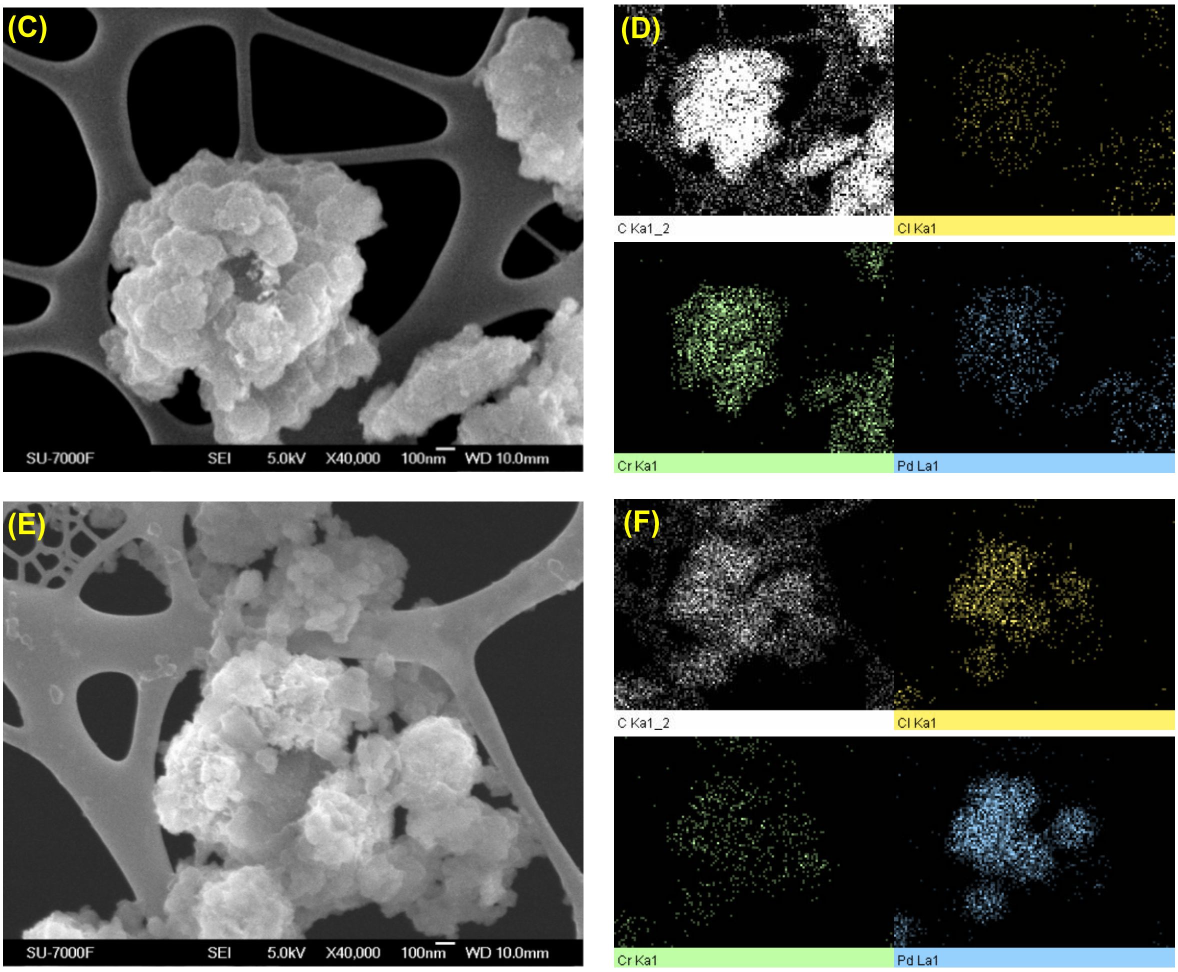

Figure S8. Analysis of recycled PdII@MIL-101(Cr)-NH2 catalyst. (A) Recycled catalysts after 10 consecutive runs. (B) PXRD patterns of recycled catalysts. (C) and (D) SEM and EDS images of recycled catalyst following method A. (E) and (F) SEM and EDS images of recycled catalyst following method B.

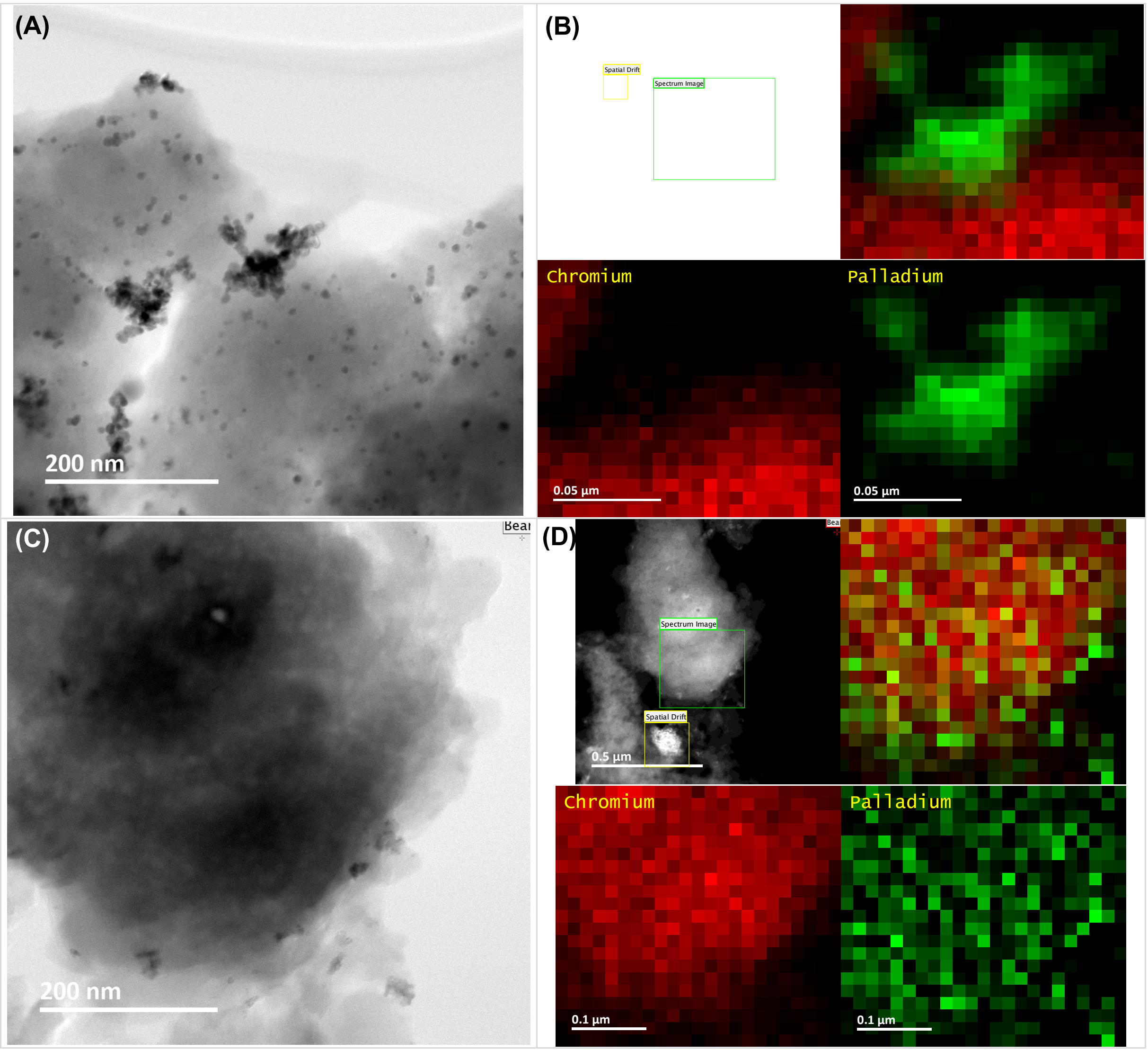

Figure S9. BF-STEM and EDS images of recycled Pd^{II}@MIL-101(Cr)-NH_2 after 10 runs following method A (A and B) and method B (C and D)

The recycled catalyst after 1 reaction run following both method A (No treatment) and method B (Oxidative treatment) was examined with SEM-EDS, N_2 gas adsorption – desorption analysis, and PXRD analysis. The experimental results were compared with those of the pristine Pd^{II}@MIL-101(Cr)-NH_2 (before catalysis)

SEM-EDS analysis (Figure S10) showed the ratio of Pd : Cr on the samples:

- The Pd : Cr ratio of pristine Pd^{II}@MIL-101(Cr)-NH_2 (before catalysis) was 17 atom% Pd per 100 atom% Cr with an even distribution of the two elements on the MOF structure (Figure S10, top).

- After 1 reaction run, without additional oxidative treatment the Pd : Cr ratio essentially remains the same, with 18 atom% Pd per 100 atom% Cr. Formation of Pd NPs and an uneven distribution of the two elements were observed (Figure S10, middle).

- After 1 reaction run and after performing one oxidative treatment to re-oxidize Pd NPs into PdII species, an even distribution of Pd on MIL-101(Cr)-NH2 support was again observed (Figure S10, bottom). The Pd : Cr ratio changed to 23 atom% : 100 atom%. This balance indicates that the amount of Cr has slightly decreased in comparison to Pd.

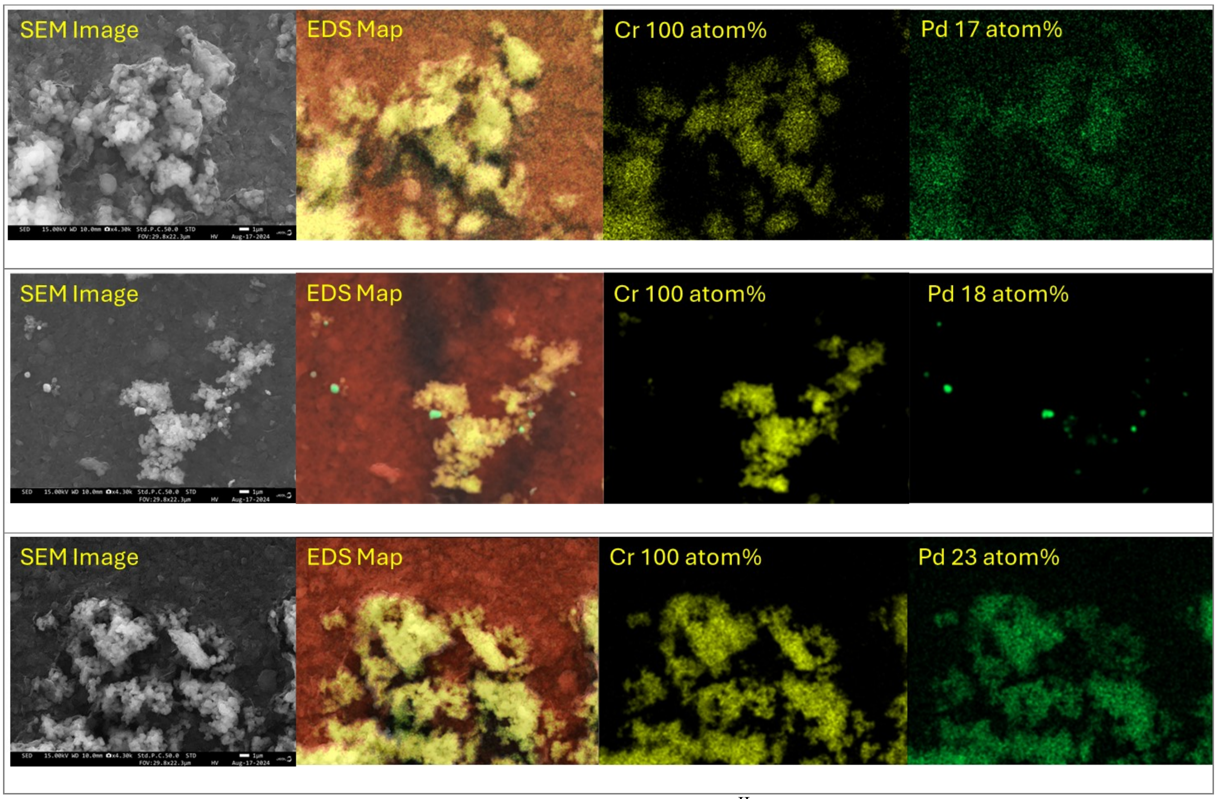

Figure S10. SEM imaging and EDS analysis of pristine Pd^{II}@MIL-101(Cr)-NH_2 (top), recycled catalyst under No treatment conditions (middle), and recycled catalyst under Oxidative treatment conditions (bottom).

The recycled catalyst under before and after oxidative treatment (after 1 run) were examined with N_2 gas adsorption – desorption analysis of (Figure S11). The recycled catalysts have lower surface areas of 1156 m^2/g (No treatment conditions) and 983 m^2/g (Oxidative treatment conditions) compared to that of 1621 m^2/g of Pd^{II}@MIL-101(Cr)-NH_2 (Figure S11A). The surface areas of the recycled catalyst remained nevertheless very high, and the isotherm type of the recycled catalysts are similar to that of the pristine catalyst. Pore size distributions of the recycled catalysts are similar to those of the pristine catalyst, although lower pore volumes as observed (Figure S11B). Hence, the catalyst structure did not significantly change after carbonylation as confirmed by porosity analysis. The lower surface area and pore volume can be attributed to the trapping of the reagents or products within the catalyst's pores.

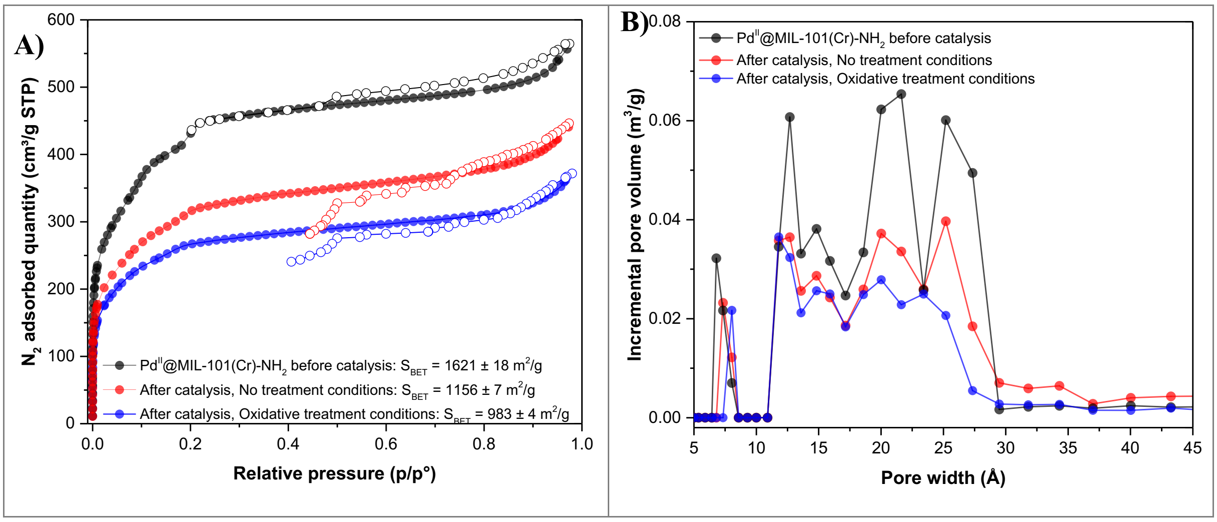

Figure S11. N_2 adsorption – desorption isotherms (A) and porosity distribution (B) of pristine Pd^{II}@MIL-101(Cr)-NH_2 and the recycled catalysts after 1 catalytic run. The adsorption – desorption isotherms in blue and red are the measurements after catalytic runs, and the deviation of the lines are most likely due to loss of adsorbed molecules (such as CO) that were present during catalytic tests. The changes indicate that the heterogeneity of the pore shape increases after the reaction.

The recycled catalysts were also examined with PXRD analysis (Figure S12). The PXRD patterns of the recycled catalysts did not show the same diffractions as those of the pristine Pd^{II}@MIL-101(Cr)-NH_2. We conclude therefore that an amorphous layer is formed on the surface of the MIL-101(Cr)-NH_2 crystals.

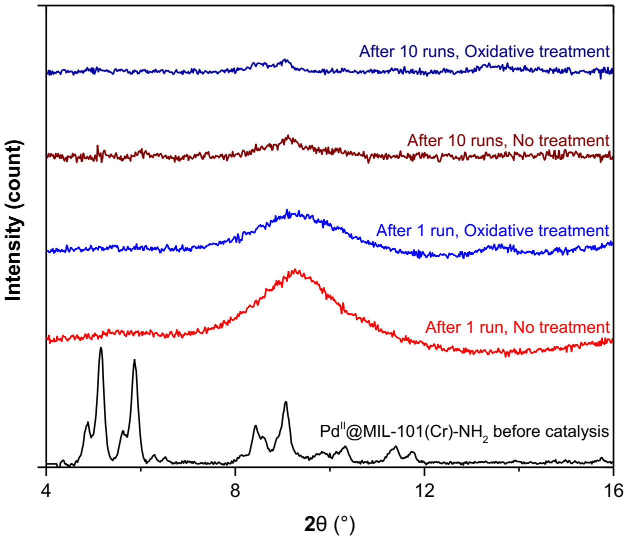

Figure S12. PXRD patterns of Pd^{II}@MIL-101(Cr)-NH_2 before catalysis, after 1 run and after 10 runs, with and without oxidative treatment.

5.2. Recycling of the 1–Br₂@Ag catalyst.

Recycling experiments were run on a 0.25 mmol scale. For the first run, CA was performed at –1.0 V RHE for 15 h in aqueous 0.1 M $KHCO_3$ with 10 mM of **1–Br$_2$** additive and a Ag working electrode,

before starting the carbonylation reaction under the typical conditions. Afterwards, the crude of the carbonylation reaction was taken out and treated under the usual conditions, the reactor and all electrodes were cleaned with DI water and dried. During this first run, the Ag WE took an orange shade, due to the electrodeposition of **1–Br₂** additive, and this functionalized electrode was named **1-Br₂**@Ag electrode to discriminate with a fresh Ag electrode.

For the following runs, the CA were carried out 15 h in 0.1 M $KHCO_3$ without **1–Br_2** additive, using **1-Br_2**@Ag electrode as working electrode, and the carbonylation conditions were kept strictly identical.

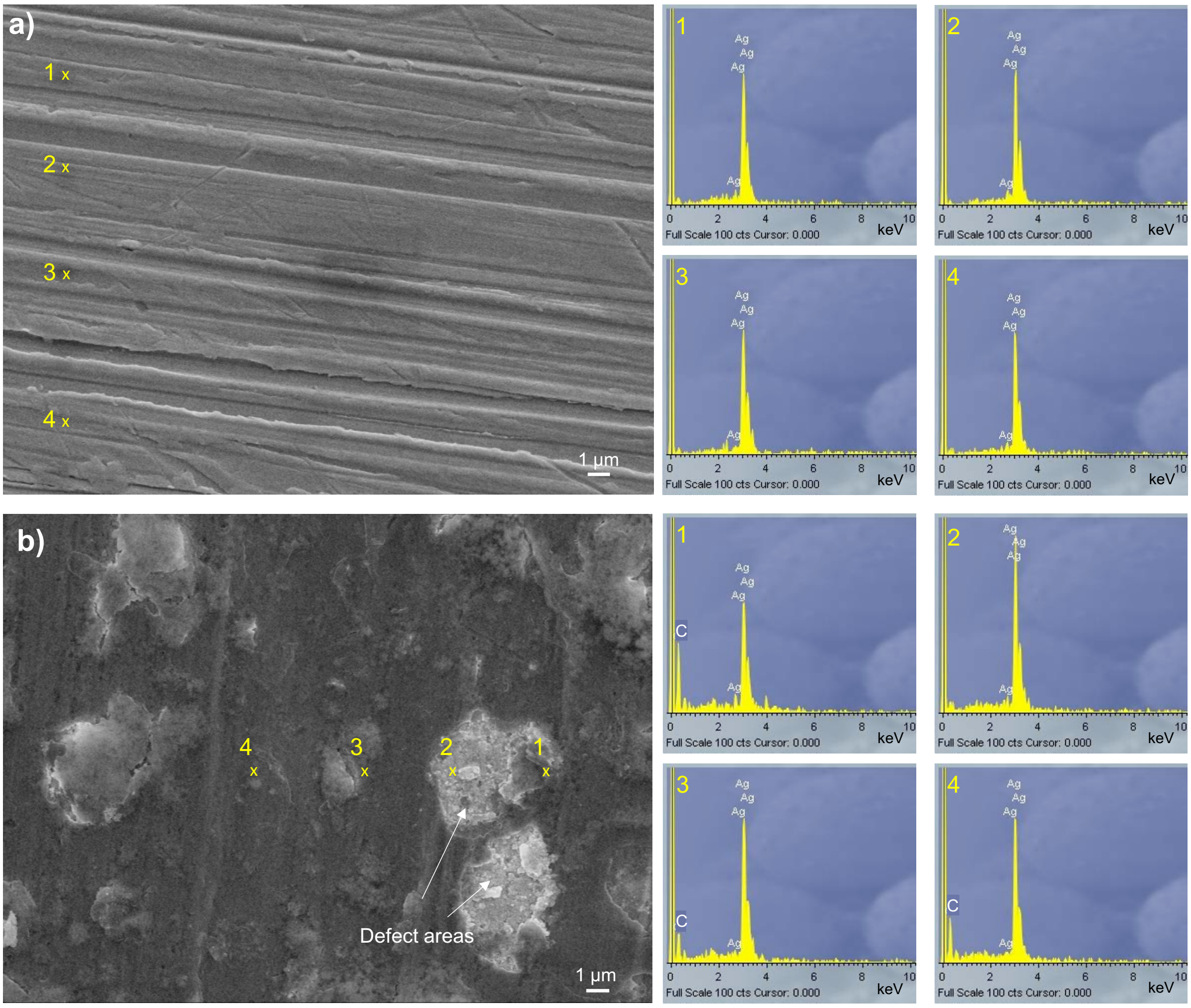

Figure S13. SEM micrographs of Ag wire before (a) and after (b) 15 h chonoamperommetry at –1.0 V RHE in 0.1 M $KHCO_3$ with 10 mM of **1–Br_2** additive. Inset show EDS spectrum recorded in the crossed-marked areas. In (b) micrographs, arrows show defective areas in the coating.

SEM images were collected on a JEOL JSM-7401F instrument using a Schottky-type field emission gun. Samples were loaded on carbon tape prior to analysis. Energy-dispersion X-ray (EDS) were collected at an accelerating voltage of 15 kV.

SEM micrographs of the Ag wire prior to chronoamperommetry (a) show the surface of Ag wire, the parallel striation observed are due to the polishing of the wire. EDS recorded on several spots only show signal of silver in the 0-20 keV window.

After 15 h of chronoamperommetry at –1.0 V *vs* RHE in aqueous $KHCO_3$ 0.1 M in presence of 10 mM of **1–Br$_2$**, the SEM micrograph clearly shows a coating on the surface of the electrode, also evidenced by contrast with defective areas shown with white arrows. EDS spectra taken on coated areas show signals of carbon in addition to silver, probably corresponding to reduced species of **1–Br$_2$** coating the silver surface. By contrast, EDS spectrum taken on a defect area shows only traces of silver, further evidencing the nature of the coated electrode.

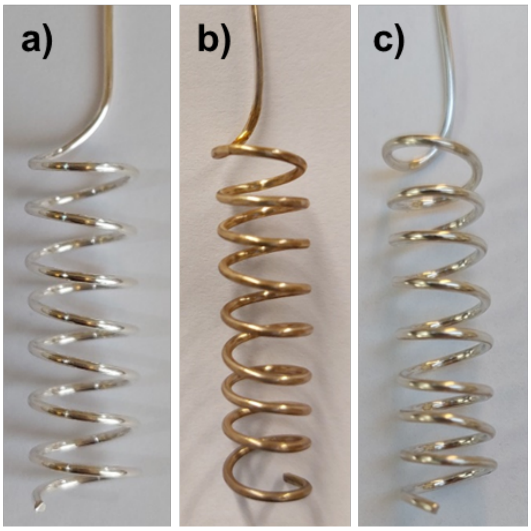

Figure S14. Optical image of the silver wire (a) before (b) after 15h CA (c) after film extraction.

6. Scale-up experiment

Scale-up experiment was done under Condition A using CO balloon, resulted in 67% yield (456 mg) of the desired product. However, due to the limit of the electrochemical cell, the scale-up reaction has not been done under Conditions **B** using our tandem set-up. Building a bigger electrochemical cell could solve the problem.

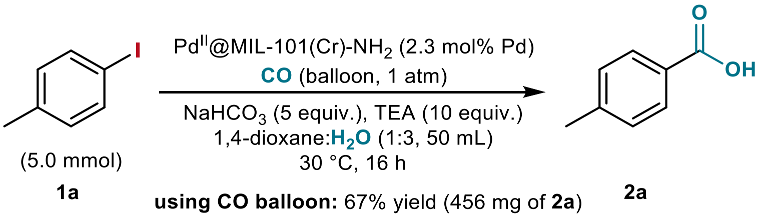

Scheme S4. Scale-up experiment using CO balloon.

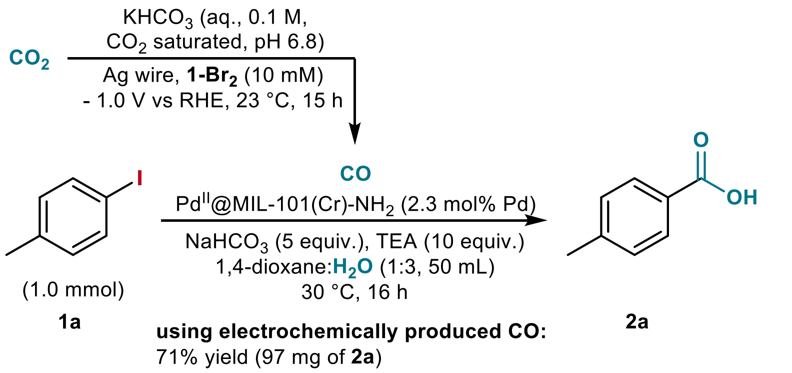

Scheme S5. Scale-up experiment using electrochemically produced CO.

7. Scope of the reactions

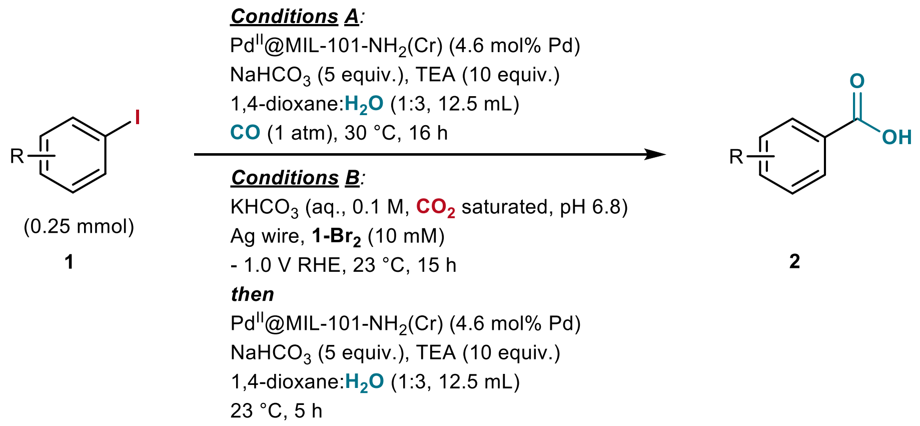

Conditions A: The general procedure for hydroxycarboxylation of aryl halides under CO atmosphere described in section 4.1 was applied for 4-iodotoluene (**1a**, 54.5 mg, 0.25 mmol). Product **2a** (30.0 mg, 88% yield) was purified by flash chromatography using n-pentane: ethyl acetate 90:10 to 50:50 solutions as eluents.

Conditions B: In the typical 3-chamber setup as described in section 4.3, the carbonylation chamber was charged with 4-iodotoluene (**1a**, 54.5 mg, 0.25 mmol), Pd^{II}@MIL-101(Cr)-NH_2 (15.2 mg, 4.6 mol% Pd loading) and $NaHCO_3$ (105.0 mg, 5 equiv., 1.25 mmol). The chamber was then adapted to the electrochemical cell filled with electrolyte and additive **1-Br₂** (202 mg; 10 mM) before starting the CA. After 15 h of CA at –1.0 V vs RHE under CO_2 atmosphere, 1,4-dioxane (3.125 mL), D.I. water (9.375 mL) and triethylamine (348 µL, 10 equiv., 2.5 mmol) were added in the carbonylation chamber. Carbonylation was run 5 h at 23 °C. After reaction, the reaction mixture was centrifuged twice for 5 min at 6000 rpm. The supernatant was then acidified to pH 1-2 with HCl 1 M before being extracted 3 times with EtOAc, dried over $MgSO_4$ and evaporated. Product **2a** (34.0 mg, 100% yield) was purified by flash chromatography using n-pentane: ethyl acetate 90:10 to 50:50 solutions as eluents. Faradaic efficiency FE = 21%.

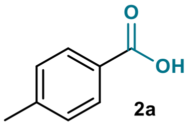 2a<br>4-methylbenzoic acid	^{1}H NMR (400 MHz, Chloroform-*d*) δ 8.02 (d, *J* = 8.2 Hz, 2H), 7.28 (d, *J* = 8.0 Hz, 2H), 2.44 (s, 3H).<br>^{13}C NMR (101 MHz, $CDCl_3$) δ 172.64, 144.80, 130.41, 129.36, 126.75, 21.90.<br>Spectroscopic data are in agreement with those in the literature.[3]

Conditions A: The general procedure for hydroxycarboxylation of aryl halides under CO atmosphere described in section 4.1 was applied for 1-iodo-4-methoxybenzene (**1b**, 58.5 mg, 0.25 mmol). Product **2b** (31.2 mg, 82% yield) was purified by flash chromatography using n-pentane: ethyl acetate 90:10 to 50:50 solutions as eluents.

Conditions B: In the typical 3-chamber setup as described in section 4.3, the carbonylation chamber was charged with 1-iodo-4-methoxybenzene (**1b**, 58.5 mg, 0.25 mmol), Pd^{II}@MIL-101(Cr)-NH_2 (15.2 mg, 4.6 mol% Pd loading) and $NaHCO_3$ (105.0 mg, 5 equiv., 1.25 mmol). The chamber was then adapted to the electrochemical cell filled with electrolyte and additive **1-Br$_2$** (202 mg; 10 mM) before starting the CA. After 15 h of CA at –1.0 V vs RHE under CO_2 atmosphere, 1,4-dioxane (3.125 mL), D.I. water (9.375 mL) and triethylamine (348 µL, 10 equiv., 2.5 mmol) were added in the carbonylation chamber. Carbonylation was run 5 h at 23 °C. After reaction, the reaction mixture was centrifuged twice for 5 min at 6000 rpm. The supernatant was then acidified to pH 1-2 with HCl 1 M before being extracted 3 times with EtOAc, dried over $MgSO_4$ and evaporated. Product **2b** (33.8 mg, 89% yield) was purified by flash chromatography using n-pentane: ethyl acetate 90:10 to 50:50 solutions as eluents. Faradaic efficiency FE = 18.4%.

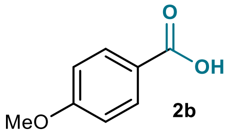 2b<br>4-methoxybenzoic acid	^{1}H NMR (400 MHz, Methanol-*d*$_4$) δ 7.99 – 7.94 (m, 2H), 7.00 – 6.94 (m, 2H), 3.85 (s, 3H).<br>^{13}C NMR (101 MHz, Methanol-*d*$_4$) δ 169.80, 165.06, 132.81, 124.01, 114.67, 55.95.<br>Spectroscopic data are in agreement with those in the literature.[4]

Conditions A: The general procedure for hydroxycarboxylation of aryl halides under CO atmosphere described in section 4.1 was applied for 4-iodophenol (**1c**, 55.0 mg, 0.25 mmol). Product **2c** (21.7 mg, 52% yield) was purified by flash chromatography using n-pentane: ethyl acetate 90:10 to 50:50 solutions as eluents.

Conditions B: In the typical 3-chamber setup as described in section 4.3, the carbonylation chamber was charged with 4-iodophenol (**1c**, 55.0 mg, 0.25 mmol), Pd^{II}@MIL-101(Cr)-NH_2 (15.2 mg, 4.6 mol% Pd loading) and $NaHCO_3$ (105.0 mg, 5 equiv., 1.25 mmol). The chamber was then adapted to the electrochemical cell filled with electrolyte and additive **1-Br$_2$** (202 mg; 10 mM) before starting the CA. After 15 h of CA at –1.0 V vs RHE under CO_2 atmosphere, 1,4-dioxane (3.125 mL), D.I. water (9.375 mL) and triethylamine (348 µL, 10 equiv., 2.5 mmol) were added in the carbonylation

chamber. Carbonylation was run 5 h at 23 °C. After reaction, the reaction mixture was centrifuged twice for 5 min at 6000 rpm. The supernatant was then acidified to pH 1-2 with HCl 1 M before being extracted 3 times with EtOAc, dried over $MgSO_4$ and evaporated. Product **2c** (25.5 mg, 74% yield) was purified by flash chromatography using n-pentane: ethyl acetate 90:10 to 50:50 solutions as eluents. Faradaic efficiency FE = 13.6%.	
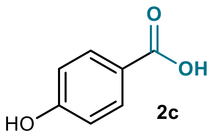<br>4-hydroxybenzoic acid	^{1}H NMR (400 MHz, Methanol-d_4) δ 7.92 – 7.84 (m, 2H), 6.85 – 6.76 (m, 2H).<br><br>^{13}C NMR (101 MHz, Methanol-d_4) δ 170.08, 163.35, 133.00, 122.71, 116.02.<br><br>Spectroscopic data are in agreement with those in the literature.[5]

**Conditions A:** The general procedure for hydroxycarboxylation of aryl halides under CO atmosphere described in section 4.1 was applied for 4-iodoaniline (**1d**, 54.8 mg, 0.25 mmol). After reaction, the solvent and volatile substances were removed under reduced pressure. The solid crude was acidified with HCl in MeOH (3 M), and 5 mL of water was added. The mixture was then extracted with EtOAc (5 mL × 5 times). Consequently, the solvent was removed under reduced pressure to yield product **2a·HCl** (30.4 mg, 70% yield).<br><br>**Conditions B:** In the typical 3-chamber setup as described in section 4.3, the carbonylation chamber was charged with 4-iodoaniline (**1d**, 54.8 mg, 0.25 mmol), Pd^{II}@MIL-101(Cr)-NH_2 (15.2 mg, 4.6 mol% Pd loading) and $NaHCO_3$ (105.0 mg, 5 equiv., 1.25 mmol). The chamber was then adapted to the electrochemical cell filled with electrolyte and additive **1-Br₂** (202 mg; 10 mM) before starting the CA. After 15 h of CA at –1.0 V vs RHE under CO_2 atmosphere, 1,4-dioxane (3.125 mL), D.I. water (9.375 mL) and triethylamine (348 µL, 10 equiv., 2.5 mmol) were added in the carbonylation chamber. Carbonylation was run 5 h at 23 °C. After reaction, the solvent and volatile substances of the carbonylation reaction were removed under reduced pressure. The solid crude was acidified with HCl in MeOH (3 M), and 5 mL of water was added. The mixture was then extracted with EtOAc (5 mL × 5 times). Consequently, the solvent was removed under reduced pressure to yield product **2a·HCl** (35.6 mg, 82% yield). Faradaic efficiency FE = 17.2%.	
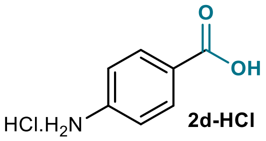<br>4-aminobenzoic acid	^{1}H NMR (400 MHz, Methanol-d_4) δ 7.77 – 7.70 (m, 1H), 6.66 – 6.60 (m, 1H).<br><br>^{13}C NMR (101 MHz, Methanol-d_4) δ 170.65, 154.47, 132.76, 119.09, 114.37.<br><br>Spectroscopic data are in agreement with those in the literature.[6]

**Conditions A:** The general procedure for hydroxycarboxylation of aryl halides under CO atmosphere described in section 4.1 was applied for 4-nitrobenzene (**1e**, 62.3 mg, 0.25 mmol). Product **2e** (40.5

mg, 97% yield) was purified by flash chromatography using n-pentane: ethyl acetate 90:10 to 50:50 solutions as eluents.

Conditions B: In the typical 3-chamber setup as described in section 4.3, the carbonylation chamber was charged with 4-nitrobenzene (**1e**, 62.3 mg, 0.25 mmol), Pd^{II}@MIL-101(Cr)-NH_2 (15.2 mg, 4.6 mol% Pd loading) and $NaHCO_3$ (105.0 mg, 5 equiv., 1.25 mmol). The chamber was then adapted to the electrochemical cell filled with electrolyte and additive **1-Br₂** (202 mg; 10 mM) before starting the CA. After 15 h of CA at –1.0 V vs RHE under CO_2 atmosphere, 1,4-dioxane (3.125 mL), D.I. water (9.375 mL) and triethylamine (348 µL, 10 equiv., 2.5 mmol) were added in the carbonylation chamber. Carbonylation was run 5 h at 23 °C. After reaction, the reaction mixture was centrifuged twice for 5 min at 6000 rpm. The supernatant was then acidified to pH 1-2 with HCl 1 M before being extracted 3 times with EtOAc, dried over $MgSO_4$ and evaporated. Product **2e** (31.3 mg, 75% yield) was purified by flash chromatography using n-pentane: ethyl acetate 90:10 to 50:50 solutions as eluents. Faradaic efficiency FE = 11.1%.

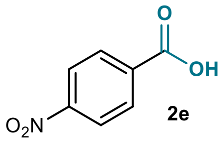

2e

4-nitrobenzoic acid

^{1}H NMR (400 MHz, Methanol-d_4) δ 8.31 (d, J = 8.7 Hz, 2H), 8.22 (d, J = 8.7 Hz, 2H).

^{13}C NMR (101 MHz, Methanol-d_4) δ 167.58, 151.95, 137.64, 131.94, 124.53.

Spectroscopic data are in agreement with those in the literature.[4]

Conditions A: The general procedure for hydroxycarboxylation of aryl halides under CO atmosphere described in section 4.1 was applied for 4-iodobenzonitrile (**1f**, 57.3 mg, 0.25 mmol). Product **2f** (30.5 mg, 83% yield) was purified by flash chromatography using n-pentane: ethyl acetate 90:10 to 50:50 solutions as eluents.

Conditions B: In the typical 3-chamber setup as described in section 4.3, the carbonylation chamber was charged with 4-iodobenzonitrile (**1f**, 57.3 mg, 0.25 mmol), Pd^{II}@MIL-101(Cr)-NH_2 (15.2 mg, 4.6 mol% Pd loading) and $NaHCO_3$ (105.0 mg, 5 equiv., 1.25 mmol). The chamber was then adapted to the electrochemical cell filled with electrolyte and additive **1-Br₂** (202 mg; 10 mM) before starting the CA. After 15 h of CA at –1.0 V vs RHE under CO_2 atmosphere, 1,4-dioxane (3.125 mL), D.I. water (9.375 mL) and triethylamine (348 µL, 10 equiv., 2.5 mmol) were added in the carbonylation chamber. Carbonylation was run 5 h at 23 °C. After reaction, the reaction mixture was centrifuged twice for 5 min at 6000 rpm. The supernatant was then acidified to pH 1-2 with HCl 1 M before being extracted 3 times with EtOAc, dried over $MgSO_4$ and evaporated. Product **2f** (22.8 mg, 62% yield) was purified by flash chromatography using n-pentane: ethyl acetate 90:10 to 50:50 solutions as eluents. Faradaic efficiency FE = 11.6%.

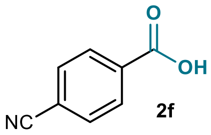

2f

^{1}H NMR (400 MHz, Methanol-d_4) δ 8.15 (d, J = 8.6 Hz, 2H), 7.83 (d, J = 8.7 Hz, 2H).

^{13}C NMR (101 MHz, Methanol-d_4) δ 167.85, 136.16, 133.44, 131.32, 119.01, 117.23.

4-cyanobenzoic acid	Spectroscopic data are in agreement with those in the literature.[5]

Conditions A: The general procedure for hydroxycarboxylation of aryl halides under CO atmosphere described in section 4.1 was applied for 1-iodo-4-(trifluoromethyl)benzene (**1g**, 68 mg, 0.25 mmol). Product **2g** (42.8 mg, 90% yield) was purified by flash chromatography using n-pentane: ethyl acetate 90:10 to 50:50 solutions as eluents.

Conditions B: In the typical 3-chamber setup as described in section 4.3, the carbonylation chamber was charged with 1-iodo-4-(trifluoromethyl)benzene (**1g**, 68 mg, 0.25 mmol), Pd^{II}@MIL-101(Cr)-NH_2 (15.2 mg, 4.6 mol% Pd loading) and $NaHCO_3$ (105.0 mg, 5 equiv., 1.25 mmol). The chamber was then adapted to the electrochemical cell filled with electrolyte and additive **1-Br₂** (202 mg; 10 mM) before starting the CA. After 15 h of CA at –1.0 V vs RHE under CO_2 atmosphere, 1,4-dioxane (3.125 mL), D.I. water (9.375 mL) and triethylamine (348 µL, 10 equiv., 2.5 mmol) were added in the carbonylation chamber. Carbonylation was run 5 h at 23 °C. After reaction, the reaction mixture was centrifuged twice for 5 min at 6000 rpm. The supernatant was then acidified to pH 1-2 with HCl 1 M before being extracted 3 times with EtOAc, dried over $MgSO_4$ and evaporated. Product **2g** (29.5 mg, 62% yield) was purified by flash chromatography using n-pentane: ethyl acetate 90:10 to 50:50 solutions as eluents. Faradaic efficiency FE = 11.9%.

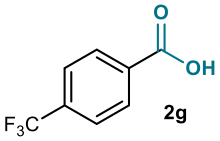

2g

4-(trifluoromethyl)benzoic acid

^{1}H NMR (400 MHz, DMSO-d_6) δ 8.16 – 8.10 (m, 2H), 7.86 (d, J = 8.3 Hz, 2H).

^{13}C NMR (101 MHz, DMSO-d_6) δ 166.20, 134.61, 132.50 (q, $J_{(C-CF)}$ = 31.9 Hz), 130.11, 125.60 (q, $J_{(C-CCF)}$ = 3.8 Hz), 123.82 (q, $^1J_{(C-F)}$ = 272.6 Hz).

Spectroscopic data are in agreement with those in the literature.[3]

Conditions A: The general procedure for hydroxycarboxylation of aryl halides under CO atmosphere described in section 4.1 was applied for methyl 4-iodobenzoate (**1h**, 65.5 mg, 0.25 mmol). Product **2h** (22.5 mg, 50% yield) was purified by flash chromatography using n-pentane: ethyl acetate 90:10 to 50:50 solutions as eluents.

Conditions B: In the typical 3-chamber setup as described in section 4.3, the carbonylation chamber was charged with 4-iodobenzoate (**1h**, 65.5 mg, 0.25 mmol), Pd^{II}@MIL-101(Cr)-NH_2 (15.2 mg, 4.6 mol% Pd loading) and $NaHCO_3$ (105.0 mg, 5 equiv., 1.25 mmol). The chamber was then adapted to the electrochemical cell filled with electrolyte and additive **1-Br₂** (202 mg; 10 mM) before starting the CA. After 15 h of CA at –1.0 V vs RHE under CO_2 atmosphere, 1,4-dioxane (3.125 mL), D.I. water (9.375 mL) and triethylamine (348 µL, 10 equiv., 2.5 mmol) were added in the carbonylation chamber. Carbonylation was run 5 h at 23 °C. After reaction, the reaction mixture was centrifuged twice for 5 min at 6000 rpm. The supernatant was then acidified to pH 1-2 with HCl 1 M before being extracted 3 times with EtOAc, dried over $MgSO_4$ and evaporated. Product **2h** (20.3 mg, 45% yield)

was purified by flash chromatography using n-pentane: ethyl acetate 90:10 to 50:50 solutions as eluents. Faradaic efficiency FE = 10.7%.

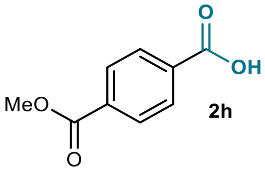 **2h** 4-(methoxycarbonyl)benzoic acid	^{1}H NMR (400 MHz, Methanol-d_4) δ 8.10 (s, 4H), 3.93 (s, 3H). ^{13}C NMR (101 MHz, MeOD) δ 168.70, 167.67, 136.09, 135.10, 130.78, 130.49, 52.90. Spectroscopic data are in agreement with those in the literature.[7]

Conditions A: The general procedure for hydroxycarboxylation of aryl halides under CO atmosphere described in section 4.1 was applied for 1-(4-iodophenyl)ethan-1-one (**1i**, 61.5 mg, 0.25 mmol). Product **2i** (39.0 mg, 95% yield) was purified by flash chromatography using n-pentane: ethyl acetate 90:10 to 50:50 solutions as eluents.

Conditions B: In the typical 3-chamber setup as described in section 4.3, the carbonylation chamber was charged with 1-(4-iodophenyl)ethan-1-one (**1i**, 61.5 mg, 0.25 mmol), Pd^{II}@MIL-101(Cr)-NH_2 (15.2 mg, 4.6 mol% Pd loading) and $NaHCO_3$ (105.0 mg, 5 equiv., 1.25 mmol). The chamber was then adapted to the electrochemical cell filled with electrolyte and additive **1-Br₂** (202 mg; 10 mM) before starting the CA. After 15 h of CA at –1.0 V vs RHE under CO_2 atmosphere, 1,4-dioxane (3.125 mL), D.I. water (9.375 mL) and triethylamine (348 µL, 10 equiv., 2.5 mmol) were added in the carbonylation chamber. Carbonylation was run 5 h at 23 °C. After reaction, the reaction mixture was centrifuged twice for 5 min at 6000 rpm. The supernatant was then acidified to pH 1-2 with HCl 1 M before being extracted 3 times with EtOAc, dried over $MgSO_4$ and evaporated. Product **2i** (36.9 mg, 90% yield) was purified by flash chromatography using n-pentane: ethyl acetate 90:10 to 50:50 solutions as eluents. Faradaic efficiency FE = 11.7%.

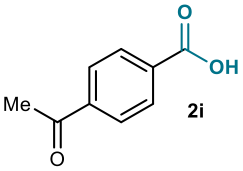 **2i** 4-acetylbenzoic acid	^{1}H NMR (400 MHz, Methanol-d_4) δ 8.12 (d, J = 8.3 Hz, 2H), 8.06 (d, J = 8.3 Hz, 2H), 2.64 (s, 3H). ^{13}C NMR (101 MHz, Methanol-d_4) δ 199.74, 168.71, 141.55, 135.96, 130.96, 129.37, 26.95. Spectroscopic data are in agreement with those in the literature.[5]

Conditions A: The general procedure for hydroxycarboxylation of aryl halides under CO atmosphere described in section 4.1 was applied for 1-fluoro-4-iodobenzene (**1j**, 55.5 mg, 0.25 mmol). Product **2j** (32.9 mg, 94% yield) was purified by flash chromatography using n-pentane: ethyl acetate 90:10 to 50:50 solutions as eluents.

Conditions B: In the typical 3-chamber setup as described in section 4.3, the carbonylation chamber was charged with 1-fluoro-4-iodobenzene (**1j**, 55.5 mg, 0.25 mmol), Pd^{II}@MIL-101(Cr)-NH_2 (15.2 mg, 4.6 mol% Pd loading) and $NaHCO_3$ (105.0 mg, 5 equiv., 1.25 mmol). The chamber was then

adapted to the electrochemical cell filled with electrolyte and additive **1-Br$_2$** (202 mg; 10 mM) before starting the CA. After 15 h of CA at –1.0 V vs RHE under CO_2 atmosphere, 1,4-dioxane (3.125 mL), D.I. water (9.375 mL) and triethylamine (348 µL, 10 equiv., 2.5 mmol) were added in the carbonylation chamber. Carbonylation was run 5 h at 23 °C. After reaction, the reaction mixture was centrifuged twice for 5 min at 6000 rpm. The supernatant was then acidified to pH 1-2 with HCl 1 M before being extracted 3 times with EtOAc, dried over $MgSO_4$ and evaporated. Product **2j** (31.5 mg, 90% yield) was purified by flash chromatography using n-pentane: ethyl acetate 90:10 to 50:50 solutions as eluents. Faradaic efficiency FE = 18.4%.

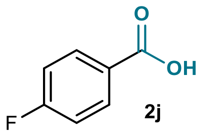

2j

4-fluorobenzoic acid

^{1}H NMR (400 MHz, Methanol-d_4) δ 8.10 – 8.04 (m, 2H), 7.19 (t, J = 8.6 Hz, 2H).

^{13}C NMR (101 MHz, Methanol-d_4) δ 168.71, 167.17 (d, $J_{(C\text{-}F)}$ = 251.8 Hz), 133.46 (d, $J_{(C\text{-}CCF)}$ = 9.4 Hz), 128.59 – 128.23, 116.38 (d, $J_{(C\text{-}CF)}$ = 22.4 Hz).

Spectroscopic data are in agreement with those in the literature.[8]

Conditions A: The general procedure for hydroxycarboxylation of aryl halides under CO atmosphere described in section 4.1 was applied for 1-chloro-4-iodobenzene (**1k**, 59.6 mg; 0.25 mmol). Product **2k** (32.9 mg, 84% yield) was purified by flash chromatography using n-pentane: ethyl acetate 90:10 to 50:50 solutions as eluents.

Conditions B: In the typical 3-chamber setup as described in section 4.3, the carbonylation chamber was charged with 1-chloro-4-iodobenzene (**1k**, 59.6 mg; 0.25 mmol), Pd^{II}@MIL-101(Cr)-NH$_2$ (15.2 mg, 4.6 mol% Pd loading) and $NaHCO_3$ (105.0 mg, 5 equiv., 1.25 mmol). The chamber was then adapted to the electrochemical cell filled with electrolyte and additive **1-Br$_2$** (202 mg; 10 mM) before starting the CA. After 15 h of CA at –1.0 V vs RHE under CO_2 atmosphere, 1,4-dioxane (3.125 mL), D.I. water (9.375 mL) and triethylamine (348 µL, 10 equiv., 2.5 mmol) were added in the carbonylation chamber. Carbonylation was run 5 h at 23 °C. After reaction, the reaction mixture was centrifuged twice for 5 min at 6000 rpm. The supernatant was then acidified to pH 1-2 with HCl 1 M before being extracted 3 times with EtOAc, dried over $MgSO_4$ and evaporated. Product **2k** (38.8 mg, 99% yield) was purified by flash chromatography using n-pentane: ethyl acetate 90:10 to 50:50 solutions as eluents. Faradaic efficiency FE = 20.8%.

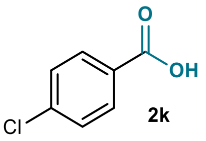

2k

4-chlorobenzoic acid

^{1}H NMR (400 MHz, Methanol-d_4) δ 8.01 – 7.97 (m, 2H), 7.51 – 7.45 (m, 2H).

^{13}C NMR (101 MHz, Methanol-d_4) δ 168.70, 140.26, 132.34, 130.75, 129.74.

Spectroscopic data are in agreement with those in the literature.[5]

Conditions A: The general procedure for hydroxycarboxylation of aryl halides under CO atmosphere described in section 4.1 was applied for 1-bromo-4-iodobenzene (**1l**, 70.7 mg; 0.25 mmol). Product

2l (42.2 mg, 84% yield) was purified by flash chromatography using n-pentane: ethyl acetate 90:10 to 50:50 solutions as eluents.

Conditions B: In the typical 3-chamber setup as described in section 4.3, the carbonylation chamber was charged with 1-bromo-4-iodobenzene (**1l**, 70.7 mg; 0.25 mmol), Pd^{II}@MIL-101(Cr)-NH_2 (15.2 mg, 4.6 mol% Pd loading) and $NaHCO_3$ (105.0 mg, 5 equiv., 1.25 mmol). The chamber was then adapted to the electrochemical cell filled with electrolyte and additive **1-Br₂** (202 mg; 10 mM) before starting the CA. After 15 h of CA at –1.0 V vs RHE under CO_2 atmosphere, 1,4-dioxane (3.125 mL), D.I. water (9.375 mL) and triethylamine (348 µL, 10 equiv., 2.5 mmol) were added in the carbonylation chamber. Carbonylation was run 5 h at 23 °C. After reaction, the reaction mixture was centrifuged twice for 5 min at 6000 rpm. The supernatant was then acidified to pH 1-2 with HCl 1 M before being extracted 3 times with EtOAc, dried over $MgSO_4$ and evaporated. Product **2l** (28.1 mg, 56% yield) was purified by flash chromatography using n-pentane: ethyl acetate 90:10 to 50:50 solutions as eluents. Faradaic efficiency FE = 12.2%.

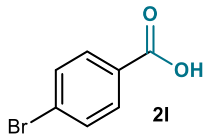

2l

4-bromobenzoic acid

^{1}H NMR (400 MHz, Methanol-d_4) δ 7.92 (d, J = 8.4 Hz, 2H), 7.64 (d, J = 8.4 Hz, 2H).

^{13}C NMR (101 MHz, Methanol-d_4) δ 168.87, 132.80, 132.46, 131.17, 128.76.

Spectroscopic data are in agreement with those in the literature.[5]

Conditions A: The general procedure for hydroxycarboxylation of aryl halides under CO atmosphere described in section 4.1 was applied for 1-iodo-3-methylbenzene (**1m**, 54.6 mg; 0.25 mmol). Product **2m** (25.2 mg, 74% yield) was purified by flash chromatography using n-pentane: ethyl acetate 90:10 to 50:50 solutions as eluents.

Conditions B: In the typical 3-chamber setup as described in section 4.3, the carbonylation chamber was charged with 1-iodo-3-methylbenzene (**1m**, 54.6 mg; 0.25 mmol), Pd^{II}@MIL-101(Cr)-NH_2 (15.2 mg, 4.6 mol% Pd loading) and $NaHCO_3$ (105.0 mg, 5 equiv., 1.25 mmol). The chamber was then adapted to the electrochemical cell filled with electrolyte and additive **1-Br₂** (202 mg; 10 mM) before starting the CA. After 15 h of CA at –1.0 V vs RHE under CO_2 atmosphere, 1,4-dioxane (3.125 mL), D.I. water (9.375 mL) and triethylamine (348 µL, 10 equiv., 2.5 mmol) were added in the carbonylation chamber. Carbonylation was run 5 h at 23 °C. After reaction, the reaction mixture was centrifuged twice for 5 min at 6000 rpm. The supernatant was then acidified to pH 1-2 with HCl 1 M before being extracted 3 times with EtOAc, dried over $MgSO_4$ and evaporated. Product **2m** (24.2 mg, 71% yield) was purified by flash chromatography using n-pentane: ethyl acetate 90:10 to 50:50 solutions as eluents. Faradaic efficiency FE = 13.2%.

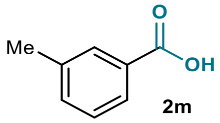

2m

3-methylbenzoic acid

^{1}H NMR (400 MHz, Methanol-d_4) δ 7.85 – 7.78 (m, 2H), 7.40 (d, J = 7.6 Hz, 1H), 7.33 (t, J = 7.6 Hz, 1H), 2.38 (s, 3H).

^{13}C NMR (101 MHz, Methanol-d_4) δ 170.04, 139.43, 134.69, 131.85, 131.17, 129.35, 127.89, 21.27.

	Spectroscopic data are in agreement with those in the literature.[5]

Conditions A: The general procedure for hydroxycarboxylation of aryl halides under CO atmosphere described in section 4.1 was applied for 1-bromo-3-iodobenzene (**1n**, 70.7 mg, 0.25 mmol). Product **2n** (31.7 mg, 63% yield) was purified by flash chromatography using n-pentane: ethyl acetate 90:10 to 50:50 solutions as eluents.

Conditions B: In the typical 3-chamber setup as described in section 4.3, the carbonylation chamber was charged with 1-bromo-3-iodobenzene (**1n**, 70.7 mg, 0.25 mmol), Pd^{II}@MIL-101(Cr)-NH_2 (15.2 mg, 4.6 mol% Pd loading) and $NaHCO_3$ (105.0 mg, 5 equiv., 1.25 mmol). The chamber was then adapted to the electrochemical cell filled with electrolyte and additive **1-Br₂** (202 mg; 10 mM) before starting the CA. After 15 h of CA at –1.0 V vs RHE under CO_2 atmosphere, 1,4-dioxane (3.125 mL), D.I. water (9.375 mL) and triethylamine (348 µL, 10 equiv., 2.5 mmol) were added in the carbonylation chamber. Carbonylation was run 5 h at 23 °C. After reaction, the reaction mixture was centrifuged twice for 5 min at 6000 rpm. The supernatant was then acidified to pH 1-2 with HCl 1 M before being extracted 3 times with EtOAc, dried over $MgSO_4$ and evaporated. Product **2n** (43.2 mg, 86% yield) was purified by flash chromatography using n-pentane: ethyl acetate 90:10 to 50:50 solutions as eluents. Faradaic efficiency FE = 18.5%.

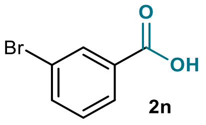

2n

3-bromobenzoic acid

^{1}H NMR (400 MHz, Methanol-d_4) δ 8.13 (t, J = 1.8 Hz, 1H), 7.97 (dt, J = 7.8, 1.3 Hz, 1H), 7.73 (ddd, J = 8.0, 2.1, 1.0 Hz, 1H), 7.39 (t, J = 7.9 Hz, 1H).

^{13}C NMR (101 MHz, Methanol-d_4) δ 168.11, 136.83, 134.23, 133.54, 131.35, 129.42, 123.29.

Spectroscopic data are in agreement with those in the literature.[7]

Conditions A: The general procedure for hydroxycarboxylation of aryl halides under CO atmosphere described in section 4.1 was applied for 1-iodo-3-methoxybenzene (**1o**, 58.5 mg, 0.25 mmol). Product **2o** (30.8 mg, 81% yield) was purified by flash chromatography using n-pentane: ethyl acetate 90:10 to 50:50 solutions as eluents.

Conditions B: In the typical 3-chamber setup as described in section 4.3, the carbonylation chamber was charged with 1-iodo-3-methoxybenzene (**1o**, 58.5 mg, 0.25 mmol), Pd^{II}@MIL-101(Cr)-NH_2 (15.2 mg, 4.6 mol% Pd loading) and $NaHCO_3$ (105.0 mg, 5 equiv., 1.25 mmol). The chamber was then adapted to the electrochemical cell filled with electrolyte and additive **1-Br₂** (202 mg; 10 mM) before starting the CA. After 15 h of CA at –1.0 V vs RHE under CO_2 atmosphere, 1,4-dioxane (3.125 mL), D.I. water (9.375 mL) and triethylamine (348 µL, 10 equiv., 2.5 mmol) were added in the carbonylation chamber. Carbonylation was run 5 h at 23 °C. After reaction, the reaction mixture was centrifuged twice for 5 min at 6000 rpm. The supernatant was then acidified to pH 1-2 with HCl 1 M before being extracted 3 times with EtOAc, dried over $MgSO_4$ and evaporated. Product **2o** (34.2 mg,

90% yield) was purified by flash chromatography using n-pentane: ethyl acetate 90:10 to 50:50 solutions as eluents. Faradaic efficiency FE = 19.6%.

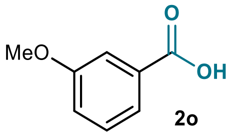

2o

3-methoxybenzoic acid

^{1}H NMR (400 MHz, Methanol-d_4) δ 7.60 (dt, J = 7.6, 1.3 Hz, 1H), 7.54 (dd, J = 2.9, 1.4 Hz, 1H), 7.36 (t, J = 7.9 Hz, 1H), 7.13 (dd, J = 8.3, 2.6 Hz, 1H), 3.82 (d, J = 1.1 Hz, 3H).

^{13}C NMR (101 MHz, Methanol-d_4) δ 169.70, 161.13, 133.24, 130.50, 123.01, 120.01, 115.45, 55.81.

Spectroscopic data are in agreement with those in the literature.[5]

Conditions A: The general procedure for hydroxycarboxylation of aryl halides under CO atmosphere described in section 4.1 was applied for 1-iodo-2-methylbenzene (**1p**, 54.5 mg; 0.25 mmol). Product **2p** (26.2 mg, 77% yield) was purified by flash chromatography using n-pentane: ethyl acetate 90:10 to 50:50 solutions as eluents.

Conditions B: In the typical 3-chamber setup as described in section 4.3, the carbonylation chamber was charged with 1-iodo-2-methylbenzene (**1p**, 54.5 mg; 0.25 mmol), Pd^{II}@MIL-101(Cr)-NH_2 (15.2 mg, 4.6 mol% Pd loading) and $NaHCO_3$ (105.0 mg, 5 equiv., 1.25 mmol). The chamber was then adapted to the electrochemical cell filled with electrolyte and additive **1-Br₂** (202 mg; 10 mM) before starting the CA. After 15 h of CA at –1.0 V vs RHE under CO_2 atmosphere, 1,4-dioxane (3.125 mL), D.I. water (9.375 mL) and triethylamine (348 µL, 10 equiv., 2.5 mmol) were added in the carbonylation chamber. Carbonylation was run 5 h at 23 °C. After reaction, the reaction mixture was centrifuged twice for 5 min at 6000 rpm. The supernatant was then acidified to pH 1-2 with HCl 1 M before being extracted 3 times with EtOAc, dried over $MgSO_4$ and evaporated. Product **2p** (17.4 mg, 51% yield) was purified by flash chromatography using n-pentane: ethyl acetate 90:10 to 50:50 solutions as eluents. Faradaic efficiency FE = 10.1%.

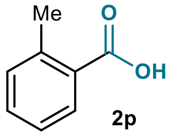

2p

2-methylbenzoic acid

^{1}H NMR (400 MHz, Methanol-d_4) δ 7.89 (dd, J = 7.7, 1.5 Hz, 1H), 7.41 (td, J = 7.5, 1.4 Hz, 1H), 7.31 – 7.20 (m, 2H), 2.57 (s, 3H).

13C NMR (101 MHz, Methanol-d_4) δ 169.77, 139.67, 131.58, 131.23, 130.30, 130.05, 125.35, 20.43.

Spectroscopic data are in agreement with those in the literature.[4]

Conditions A: The general procedure for hydroxycarboxylation of aryl halides under CO atmosphere described in section 4.1 was applied for 1-bromo-2-iodobenzene (**1q**, 70.7 mg, 0.25 mmol). Product **2q** (30.2 mg, 60% yield) was purified by flash chromatography using n-pentane: ethyl acetate 90:10 to 50:50 solutions as eluents.

Conditions B: In the typical 3-chamber setup as described in section 4.3, the carbonylation chamber was charged with 1-bromo-2-iodobenzene (**1q**, 70.7 mg, 0.25 mmol), Pd^{II}@MIL-101(Cr)-NH_2 (15.2

mg, 4.6 mol% Pd loading) and $NaHCO_3$ (105.0 mg, 5 equiv., 1.25 mmol). The chamber was then adapted to the electrochemical cell filled with electrolyte and additive **1-Br₂** (202 mg; 10 mM) before starting the CA. After 15 h of CA at –1.0 V vs RHE under CO_2 atmosphere, 1,4-dioxane (3.125 mL), D.I. water (9.375 mL) and triethylamine (348 µL, 10 equiv., 2.5 mmol) were added in the carbonylation chamber. Carbonylation was run 5 h at 23 °C. After reaction, the reaction mixture was centrifuged twice for 5 min at 6000 rpm. The supernatant was then acidified to pH 1-2 with HCl 1 M before being extracted 3 times with EtOAc, dried over $MgSO_4$ and evaporated. Product **2q** (49.7 mg, 99% yield) was purified by flash chromatography using n-pentane: ethyl acetate 90:10 to 50:50 solutions as eluents. Faradaic efficiency FE = 21%.

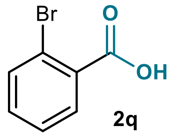

2q

2-bromobenzoic acid

^{1}H NMR (400 MHz, Methanol-d_4) δ 7.79 (dd, J = 7.5, 1.9 Hz, 1H), 7.68 (dd, J = 7.8, 1.4 Hz, 1H), 7.44 – 7.35 (m, 1H).

^{13}C NMR (101 MHz, Methanol-d_4) δ 169.57, 135.23, 133.51, 132.05, 130.70, 128.42, 121.94.

Spectroscopic data are in agreement with those in the literature.[8]

Conditions A: The general procedure for hydroxycarboxylation of aryl halides under CO atmosphere described in section 4.1 was applied for 1-iodo-2-methoxybenzene (**1r**, 58.5 mg, 0.25 mmol). Product **2r** (32.3 mg, 85% yield) was purified by flash chromatography using n-pentane: ethyl acetate 90:10 to 50:50 solutions as eluents.

Conditions B: In the typical 3-chamber setup as described in section 4.3, the carbonylation chamber was charged with 1-iodo-2-methoxybenzene (**1r**, 58.5 mg, 0.25 mmol), Pd^{II}@MIL-101(Cr)-NH_2 (15.2 mg, 4.6 mol% Pd loading) and $NaHCO_3$ (105.0 mg, 5 equiv., 1.25 mmol). The chamber was then adapted to the electrochemical cell filled with electrolyte and additive **1-Br₂** (202 mg; 10 mM) before starting the CA. After 15 h of CA at –1.0 V vs RHE under CO_2 atmosphere, 1,4-dioxane (3.125 mL), D.I. water (9.375 mL) and triethylamine (348 µL, 10 equiv., 2.5 mmol) were added in the carbonylation chamber. Carbonylation was run 5 h at 23 °C. After reaction, the reaction mixture was centrifuged twice for 5 min at 6000 rpm. The supernatant was then acidified to pH 1-2 with HCl 1 M before being extracted 3 times with EtOAc, dried over $MgSO_4$ and evaporated. Product **2r** (27.0 mg, 71% yield) was purified by flash chromatography using n-pentane: ethyl acetate 90:10 to 50:50 solutions as eluents. Faradaic efficiency FE = 17%.

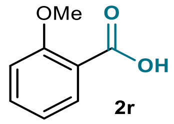

2r

2-methoxybenzoic acid

^{1}H NMR (400 MHz, Methanol-d_4) δ 7.82 (dd, J = 7.8, 1.8 Hz, 1H), 7.53 (ddd, J = 8.5, 7.4, 1.8 Hz, 1H), 7.12 (dd, J = 8.4, 1.0 Hz, 1H), 7.01 (td, J = 7.5, 1.0 Hz, 1H), 3.91 (s, 3H).

^{13}C NMR (101 MHz, Methanol-d_4) δ 169.56, 160.45, 135.14, 132.87, 121.48, 120.95, 113.31, 56.46.

Spectroscopic data are in agreement with those in the literature.[3]

Conditions A: The general procedure for hydroxycarboxylation of aryl halides under CO atmosphere described in section 4.1 was applied for 1,3-diiodobenzene (**1s**, 82.5 mg, 0.25 mmol). Product **2s** (14.5 mg, 35% yield) was purified by flash chromatography using n-pentane: ethyl acetate 90:10 to 20:80 solutions as eluents.

Conditions B: In the typical 3-chamber setup as described in section 4.3, the carbonylation chamber was charged with 1,3-diiodobenzene (**1s**, 82.5 mg, 0.25 mmol), Pd^{II}@MIL-101(Cr)-NH_2 (15.2 mg, 4.6 mol% Pd loading) and $NaHCO_3$ (105.0 mg, 5 equiv., 1.25 mmol). The chamber was then adapted to the electrochemical cell filled with electrolyte and additive **1-Br₂** (202 mg; 10 mM) before starting the CA. After 15 h of CA at –1.0 V vs RHE under CO_2 atmosphere, 1,4-dioxane (3.125 mL), D.I. water (9.375 mL) and triethylamine (348 µL, 10 equiv., 2.5 mmol) were added in the carbonylation chamber. Carbonylation was run 5 h at 23 °C. After reaction, the reaction mixture was centrifuged twice for 5 min at 6000 rpm. The supernatant was then acidified to pH 1-2 with HCl 1 M before being extracted 3 times with EtOAc, dried over $MgSO_4$ and evaporated. Product **2s** (26.2 mg, 63% yield) was purified by flash chromatography using n-pentane: ethyl acetate 90:10 to 20:80 solutions as eluents. Faradaic efficiency FE = 13%.

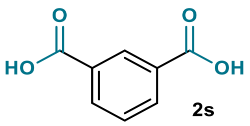 **2s**<br>isophthalic acid	^{1}H NMR (400 MHz, DMSO-d_6) δ 8.48 (t, J = 1.8 Hz, 1H), 8.16 (dd, J = 7.7, 1.8 Hz, 2H), 7.63 (t, J = 7.7 Hz, 1H).<br>^{13}C NMR (101 MHz, DMSO-d_6) δ 166.67, 133.40, 131.31, 130.01, 129.17.<br>Spectroscopic data are in agreement with those in the literature.[9]
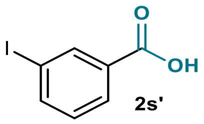 **2s'**<br>3-iodobenzoic acid	^{1}H NMR (400 MHz, Methanol-d_4) δ 8.33 (d, J = 1.7 Hz, 1H), 8.01 (d, J = 7.8 Hz, 1H), 7.94 (dd, J = 7.9, 1.6 Hz, 1H), 7.26 (t, J = 7.8 Hz, 1H).<br>^{13}C NMR (101 MHz, Methanol-d_4) δ 167.88, 142.86, 139.69, 131.37, 129.98, 94.39.<br>Spectroscopic data are in agreement with those in the literature.[10]

Conditions A: The general procedure for hydroxycarboxylation of aryl halides under CO atmosphere described in section 4.1 was applied for 2-iodothiophene (**1t**, 52.5 mg, 0.25 mmol). Yield of product **2t** (6% yield) determined by ^{1}H NMR.

Conditions B: In the typical 3-chamber setup as described in section 4.3, the carbonylation chamber was charged with 2-iodothiophene (**1t**, 52.5 mg, 0.25 mmol), Pd^{II}@MIL-101(Cr)-NH_2 (15.2 mg, 4.6 mol% Pd loading) and $NaHCO_3$ (105.0 mg, 5 equiv., 1.25 mmol). The chamber was then adapted to the electrochemical cell filled with electrolyte and additive **1-Br₂** (202 mg; 10 mM) before starting the CA. After 15 h of CA at –1.0 V vs RHE under CO_2 atmosphere, 1,4-dioxane (3.125 mL), D.I. water (9.375 mL) and triethylamine (348 µL, 10 equiv., 2.5 mmol) were added in the carbonylation chamber. Carbonylation was run 5 h at 23 °C. After reaction, the reaction mixture was centrifuged twice for 5 min at 6000 rpm. The supernatant was then acidified to pH 1-2 with HCl 1 M before being extracted 3 times with EtOAc, dried over $MgSO_4$ and evaporated. Yield of product **2t** (2% yield) was determined by ^{1}H NMR. Faradaic efficiency FE = 0.4%.

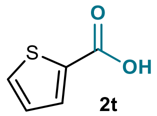 2t<br>thiophene-2-carboxylic acid	^{1}H NMR (400 MHz, Methanol-d_4) δ 7.77 (dd, J = 3.7, 1.3 Hz, 1H), 7.72 (dd, J = 5.0, 1.3 Hz, 1H), 7.14 (dd, J = 5.0, 3.7 Hz, 1H).<br>^{13}C NMR (101 MHz, Methanol-d_4) δ 165.36, 135.73, 134.56, 133.82, 128.89.<br>Spectroscopic data are in agreement with those in the literature.[8]

Conditions A: The general procedure for hydroxycarboxylation of aryl halides under CO atmosphere described in section 4.1 was applied for 2-iodofuran (**1u**, 48.5 mg, 0.25 mmol). The yield of product **2u** (49%) was determined by ^{1}H NMR.

Conditions B: In the typical 3-chamber setup as described in section 4.3, the carbonylation chamber was charged with 2-iodofuran (**1u**, 48.5 mg, 0.25 mmol), Pd^{II}@MIL-101(Cr)-NH$_2$ (15.2 mg, 4.6 mol% Pd loading) and NaHCO$_3$ (105.0 mg, 5 equiv., 1.25 mmol). The chamber was then adapted to the electrochemical cell filled with electrolyte and additive **1-Br$_2$** (202 mg; 10 mM) before starting the CA. After 15 h of CA at –1.0 V vs RHE under CO$_2$ atmosphere, 1,4-dioxane (3.125 mL), D.I. water (9.375 mL) and triethylamine (348 µL, 10 equiv., 2.5 mmol) were added in the carbonylation chamber. Carbonylation was run 5 h at 23 °C. After reaction, the reaction mixture was centrifuged twice for 5 min at 6000 rpm. The supernatant was then acidified to pH 1-2 with HCl 1 M before being extracted 3 times with EtOAc, dried over MgSO$_4$ and evaporated. The yield of product **2u** (57%) was determined by ^{1}H NMR. Faradaic efficiency FE = 9.6%.

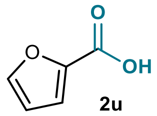 2u<br>furan-2-carboxylic acid	^{1}H NMR (400 MHz, Methanol-d_4) δ 7.71 (d, J = 2.4 Hz, 1H), 7.19 (dd, J = 3.5, 0.9 Hz, 2H), 6.58 (dd, J = 3.5, 1.8 Hz, 2H).<br>^{13}C NMR (101 MHz, Methanol-d_4) δ 161.98, 147.91, 146.65, 118.89, 112.92.<br>Spectroscopic data are in agreement with those in the literature.[8]

Conditions A: The general procedure for hydroxycarboxylation of aryl halides under CO atmosphere described in section 4.1 was applied for 2-iodopyridine (**1v**, 51.3 mg, 0.25 mmol). No desired product was observed.

Conditions B: In the typical 3-chamber setup as described in section 4.3, the carbonylation chamber was charged with 2-iodopyridine (**1v**, 51.3 mg, 0.25 mmol), Pd^{II}@MIL-101(Cr)-NH$_2$ (15.2 mg, 4.6 mol% Pd loading) and NaHCO$_3$ (105.0 mg, 5 equiv., 1.25 mmol). The chamber was then adapted to the electrochemical cell filled with electrolyte and additive **1-Br$_2$** (202 mg; 10 mM) before starting the CA. After 15 h of CA at –1.0 V vs RHE under CO$_2$ atmosphere, 1,4-dioxane (3.125 mL), D.I. water (9.375 mL) and triethylamine (348 µL, 10 equiv., 2.5 mmol) were added in the carbonylation chamber. Carbonylation was run 5 h at 23 °C. After reaction, the reaction mixture was centrifuged twice for 5 min at 6000 rpm. The supernatant was then acidified to pH 1-2 with HCl 1 M before being

extracted 3 times with EtOAc, dried over $MgSO_4$ and evaporated. No desired product was observed. Faradaic efficiency FE = 0%.

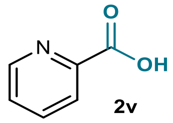 2v<br>picolinic acid	Not observed

Conditions A: The general procedure for hydroxycarboxylation of aryl halides under CO atmosphere described in section 4.1 was applied for 3-iodopyridine (**1w**, 51.3 mg, 0.25 mmol). Product **2w** (25.2 mg, 82% yield) was converted to ethyl nicotinate ester **2w-Et** with EtOH *via* Fischer esterification. The ester was purified by column chromatography using EtOAc/n-pentane 10:90 to 30:70 solutions as eluents.

Conditions B: In the typical 3-chamber setup as described in section 4.3, the carbonylation chamber was charged with 3-iodopyridine (**1w**, 51.3 mg, 0.25 mmol), Pd^{II}@MIL-101(Cr)-NH_2 (15.2 mg, 4.6 mol% Pd loading) and $NaHCO_3$ (105.0 mg, 5 equiv., 1.25 mmol). The chamber was then adapted to the electrochemical cell filled with electrolyte and additive **1-Br₂** (202 mg; 10 mM) before starting the CA. After 15 h of CA at –1.0 V vs RHE under CO_2 atmosphere, 1,4-dioxane (3.125 mL), D.I. water (9.375 mL) and triethylamine (348 µL, 10 equiv., 2.5 mmol) were added in the carbonylation chamber. Carbonylation was run 5 h at 23 °C. After reaction, the reaction mixture was centrifuged twice for 5 min at 6000 rpm. The supernatant was then acidified to pH 1-2 with HCl 1 M before being extracted 3 times with EtOAc, dried over $MgSO_4$ and evaporated. Product **2w** (30.1 mg, 98% yield) was converted to ethyl nicotinate ester **2w-Et** with EtOH *via* Fischer esterification. The ester was purified by column chromatography using EtOAc/n-pentane 10:90 to 30:70 solutions as eluents. Faradaic efficiency FE = 18.4%.

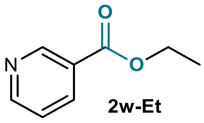 2w-Et<br>ethyl nicotinate	^{1}H NMR (400 MHz, Chloroform-*d*) δ 9.23 (dd, *J* = 2.2, 0.9 Hz, 1H), 8.77 (dd, *J* = 4.9, 1.8 Hz, 1H), 8.30 (dt, *J* = 7.9, 2.0 Hz, 1H), 7.39 (ddd, *J* = 7.9, 4.9, 0.9 Hz, 1H), 4.42 (q, *J* = 7.1 Hz, 2H), 1.42 (t, *J* = 7.1 Hz, 3H).<br><br>^{13}C NMR (101 MHz, Chloroform-*d*) δ 165.45, 153.48, 151.08, 137.16, 126.49, 123.39, 61.60, 14.42.<br><br>Spectroscopic data are in agreement with those in the literature.[11]

Conditions A: The general procedure for hydroxycarboxylation of aryl halides under CO atmosphere described in section 4.1 was applied for 4-iodopyridine (**1x**, 51.3 mg, 0.25 mmol). Product **2x** (15.4 mg, 50% yield) was converted to an ester with EtOH *via* Fischer esterification. The ester was purified by column chromatography using EtOAc/n-pentane 10:90 to 30:70 solutions as eluents.

Conditions B: In the typical 3-chamber setup as described in section 4.3, the carbonylation chamber was charged with 4-iodopyridine (**1x**, 51.3 mg, 0.25 mmol), Pd^{II}@MIL-101(Cr)-NH_2 (15.2 mg, 4.6 mol% Pd loading) and $NaHCO_3$ (105.0 mg, 5 equiv., 1.25 mmol). The chamber was then adapted to

the electrochemical cell filled with electrolyte and additive **1-Br$_2$** (202 mg; 10 mM) before starting the CA. After 15 h of CA at –1.0 V vs RHE under CO_2 atmosphere, 1,4-dioxane (3.125 mL), D.I. water (9.375 mL) and triethylamine (348 µL, 10 equiv., 2.5 mmol) were added in the carbonylation chamber. Carbonylation was run 5 h at 23 °C. After reaction, the reaction mixture was centrifuged twice for 5 min at 6000 rpm. The supernatant was then acidified to pH 1-2 with HCl 1 M before being extracted 3 times with EtOAc, dried over $MgSO_4$ and evaporated. Product **2x** (12.3 mg, 40% yield) was converted to an ester with EtOH *via* Fischer esterification. The ester was purified by column chromatography using EtOAc/n-pentane 10:90 to 30:70 solutions as eluents. Faradaic efficiency FE = 8.8%.

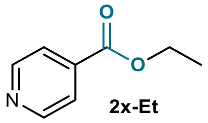

2x-Et

ethyl isonicotinate

^{1}H NMR (400 MHz, Chloroform-*d*) δ 8.82 – 8.73 (m, 2H), 7.86 – 7.83 (m, 2H), 4.42 (q, *J* = 7.1 Hz, 2H), 1.41 (t, *J* = 7.1 Hz, 3H).

^{13}C NMR (101 MHz, $CDCl_3$) δ 165.27, 150.71, 137.79, 122.99, 61.97, 14.34.

Spectroscopic data are in agreement with those in the literature.[11]

Conditions A: The general procedure for hydroxycarboxylation of aryl halides under CO atmosphere described in section 4.1 was applied for 4-iodophenyl oleate (**1y**, 121 mg, 0.25 mmol). Product **2y** (23.1 mg, 23% yield) was purified by column chromatography using n-pentane: ethyl acetate 90:10 to 50:50 solutions as eluents.

Conditions B: In the typical 3-chamber setup as described in section 4.3, the carbonylation chamber was charged with 4-iodophenyl oleate (**1y**, 121 mg, 0.25 mmol), Pd^{II}@MIL-101(Cr)-NH_2 (15.2 mg, 4.6 mol% Pd loading) and $NaHCO_3$ (105.0 mg, 5 equiv., 1.25 mmol). The chamber was then adapted to the electrochemical cell filled with electrolyte and additive **1-Br$_2$** (202 mg; 10 mM) before starting the CA. After 15 h of CA at –1.0 V vs RHE under CO_2 atmosphere, 1,4-dioxane (3.125 mL), D.I. water (9.375 mL) and triethylamine (348 µL, 10 equiv., 2.5 mmol) were added in the carbonylation chamber. Carbonylation was run 5 h at 23 °C. After reaction, the reaction mixture was centrifuged twice for 5 min at 6000 rpm. The supernatant was then acidified to pH 1-2 with HCl 1 M before being extracted 3 times with EtOAc, dried over $MgSO_4$ and evaporated. Only trace amount of product **2y** (2% yield) was observed in ^{1}H NMR. Faradaic efficiency FE = 0.3%.

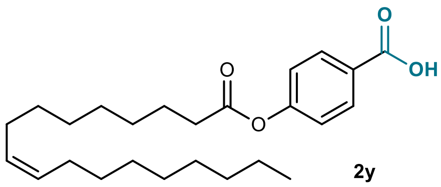

2y

4-(oleoyloxy)benzoic acid

^{1}H NMR (400 MHz, Chloroform-*d*) δ 8.05 (d, *J* = 8.3 Hz, 2H), 7.13 (d, *J* = 8.3 Hz, 2H), 5.34 – 5.30 (m, 2H), 2.54 (t, *J* = 7.5 Hz, 2H), 2.02 – 1.96 (m, 4H), 1.72 (p, *J* = 7.5 Hz, 2H), 1.35 – 1.19 (m, 20H), 0.84 (t, *J* = 6.7 Hz, 3H).

^{13}C NMR (101 MHz, Chloroform-*d*) δ 172.02, 154.55, 131.61, 130.14, 129.77, 127.71, 121.64, 34.46, 33.81, 31.97, 29.83, 29.76, 29.74, 29.58, 29.38, 29.21, 29.14, 29.12, 27.29, 27.21, 25.64, 24.95, 24.90, 22.74, 14.15.

8. NMR spectra

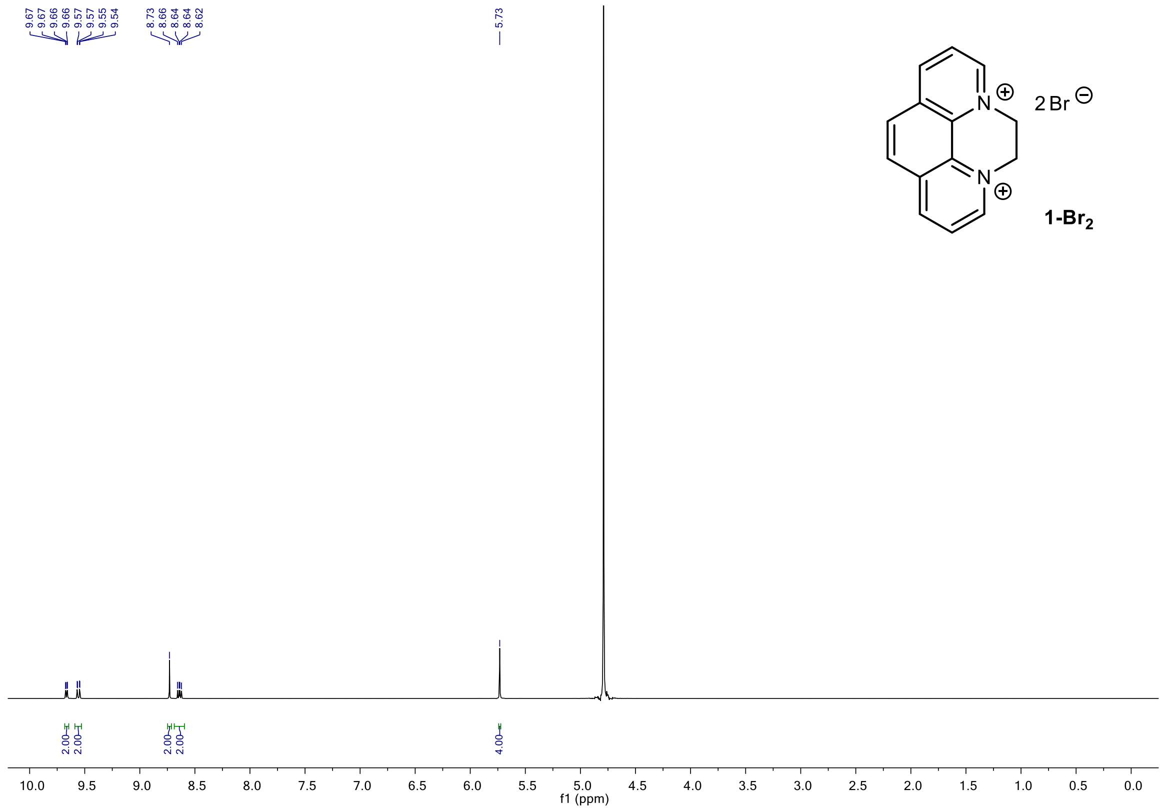

Figure S15: ^{1}H NMR (400 MHz, D_2O) spectrum of *N,N*-ethylene-phenanthrolinium dibromide (**1-Br$_2$**).

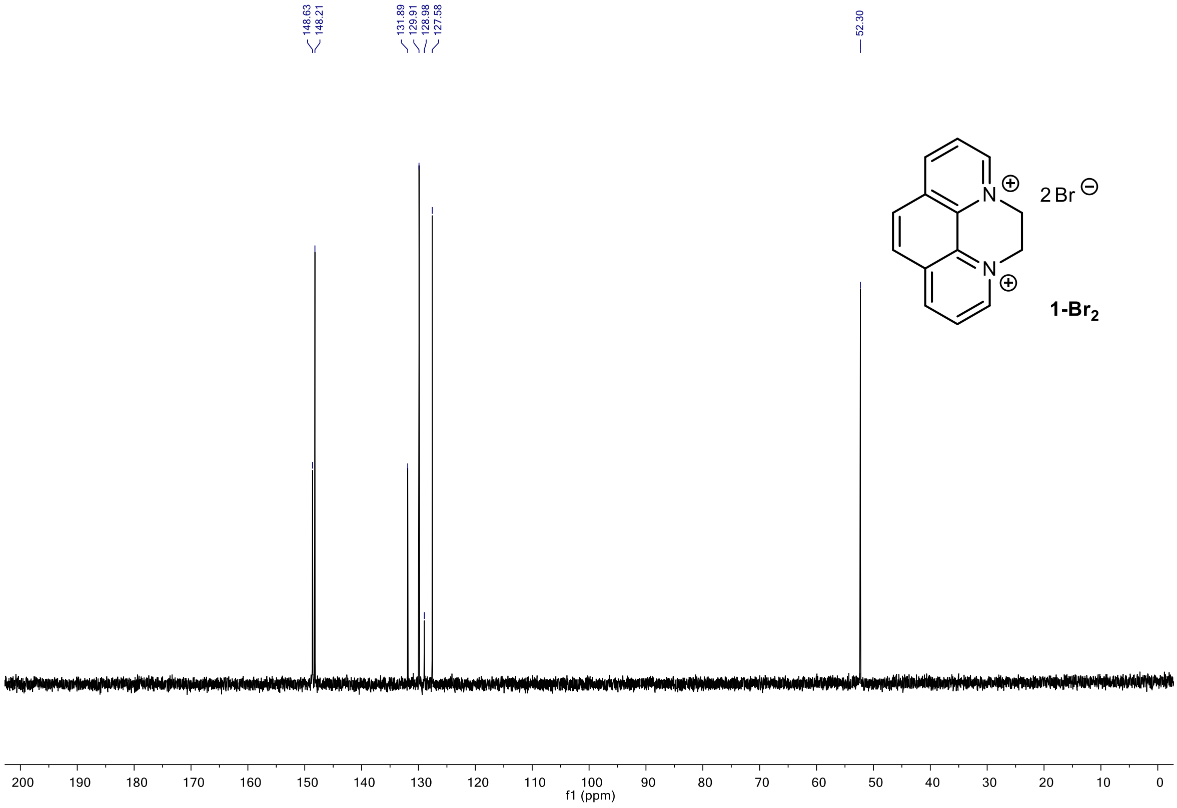

Figure S16: ^{13}C NMR (101 MHz, D_2O) spectrum of *N,N*-ethylene-phenanthrolinium dibromide (**1-Br$_2$**).

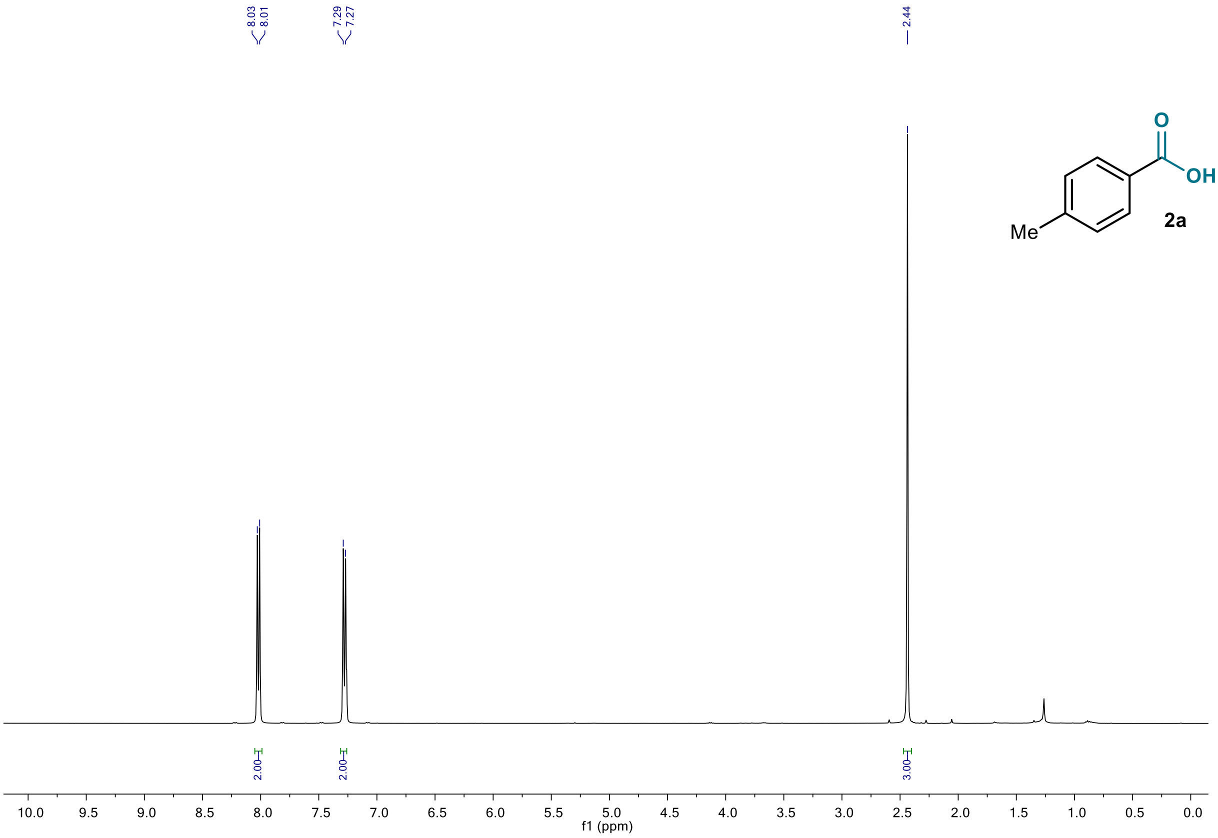

Figure S17: ^{1}H NMR (400 MHz, Methanol-d_4) spectrum of 4-methylbenzoic acid (**2a**).

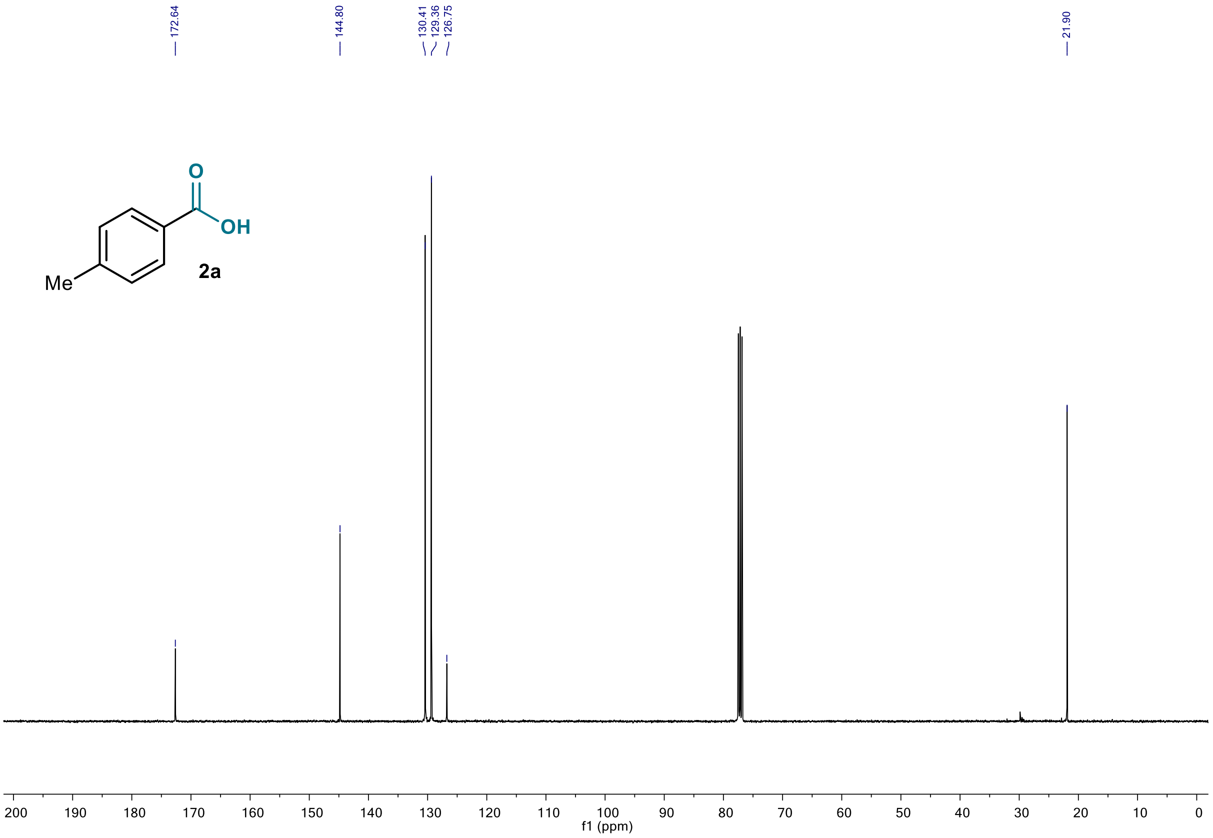

Figure S18: ^{13}C NMR (101 MHz, Methanol-d_4) spectrum of 4-methylbenzoic acid (**2a**).

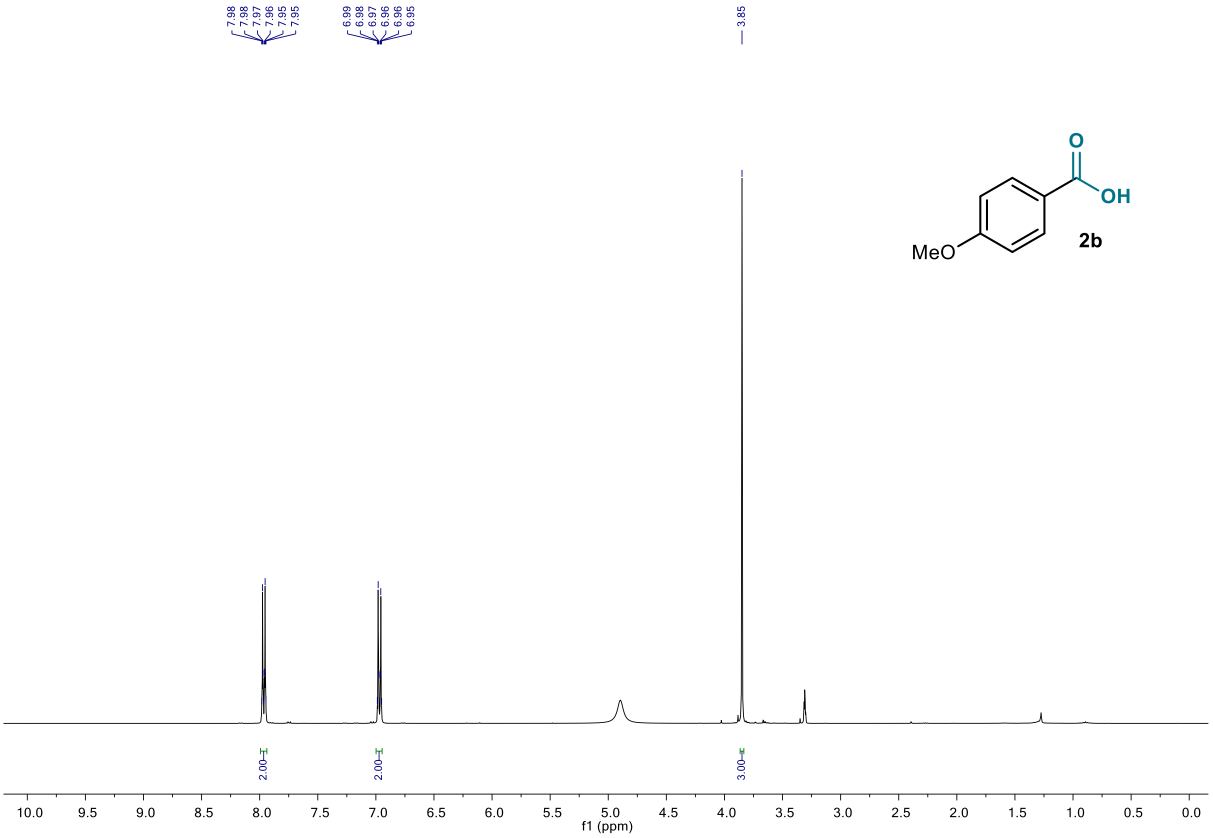

Figure S19: ^{1}H NMR (400 MHz, Methanol-d_4) spectrum of 4-methoxylbenzoic acid (**2b**).

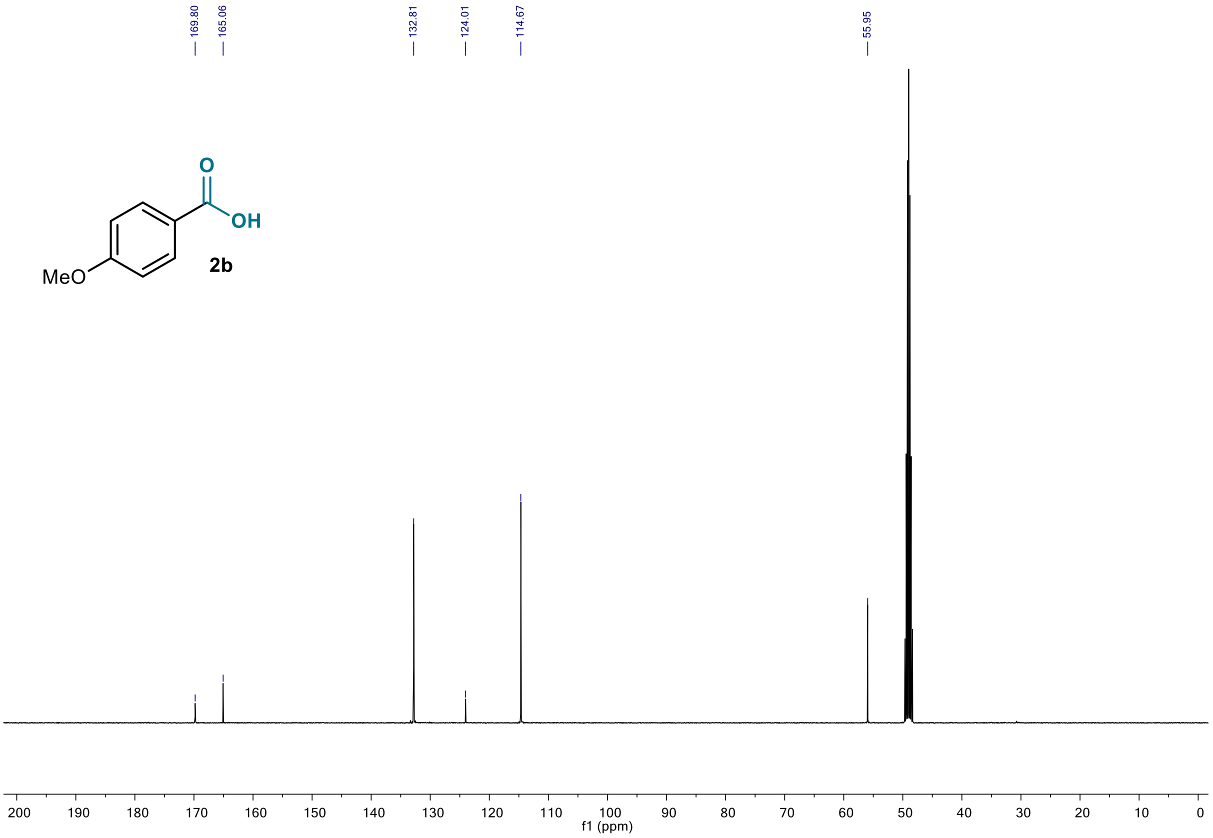

Figure S20: ^{13}C NMR (101 MHz, Methanol-d_4) spectrum of 4-methoxylbenzoic acid (**2b**).

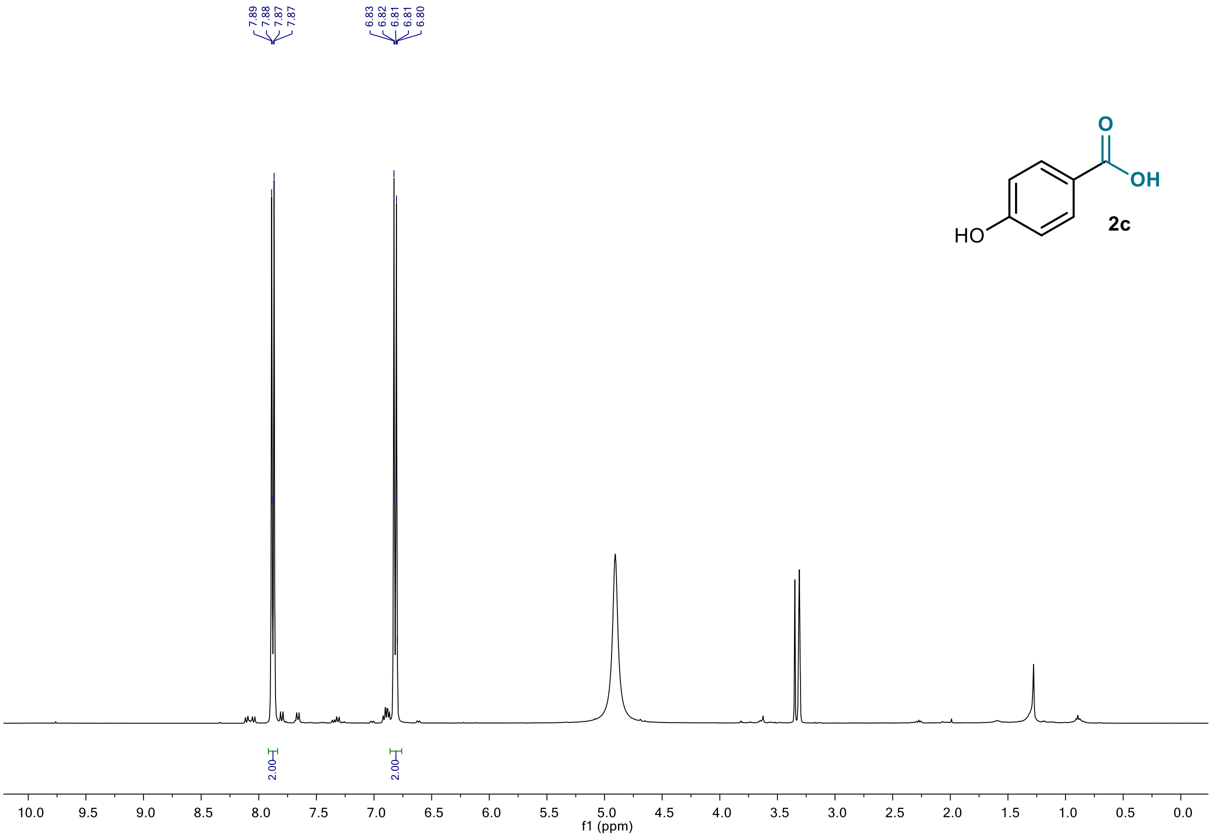

Figure S21: ^{1}H NMR (400 MHz, Methanol-d_4) spectrum of 4-hydroxybenzoic acid (**2c**).

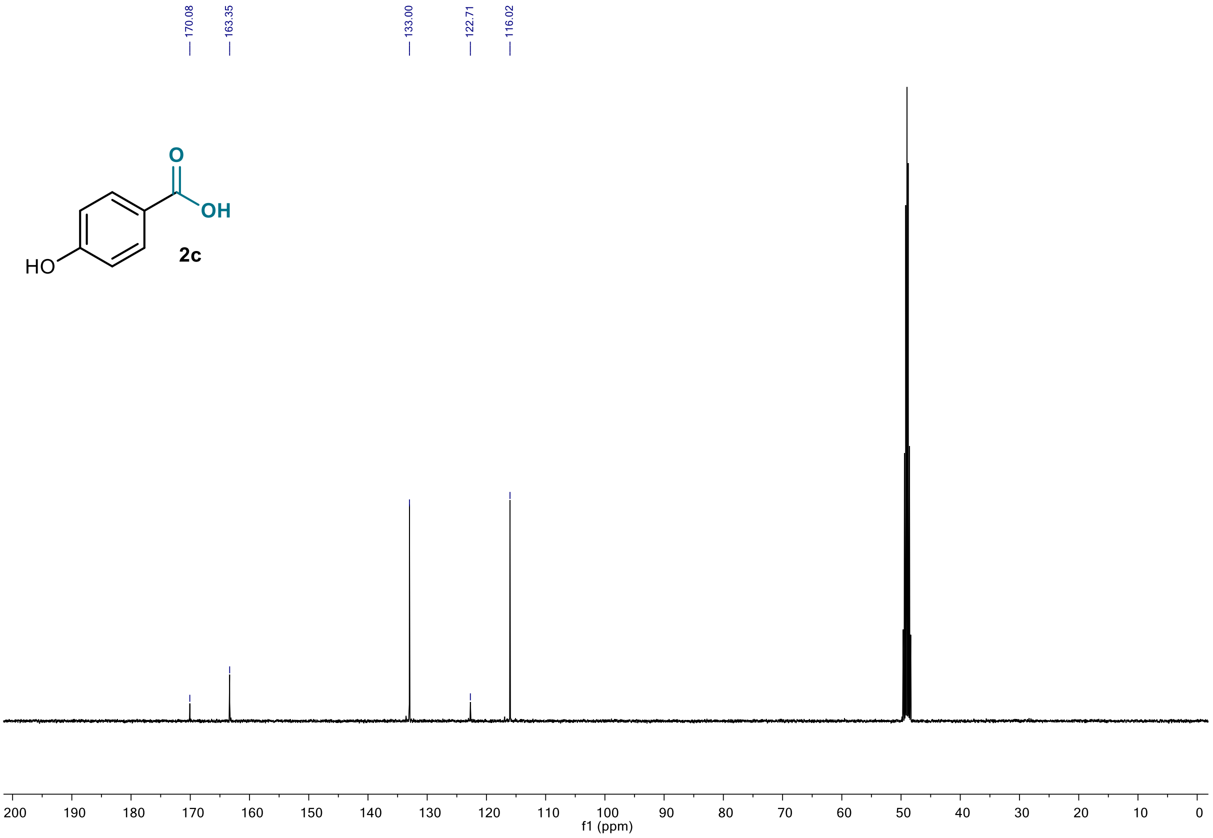

Figure S22: ^{13}C NMR (101 MHz, Methanol-d_4) spectrum of 4-hydroxybenzoic acid (**2c**).

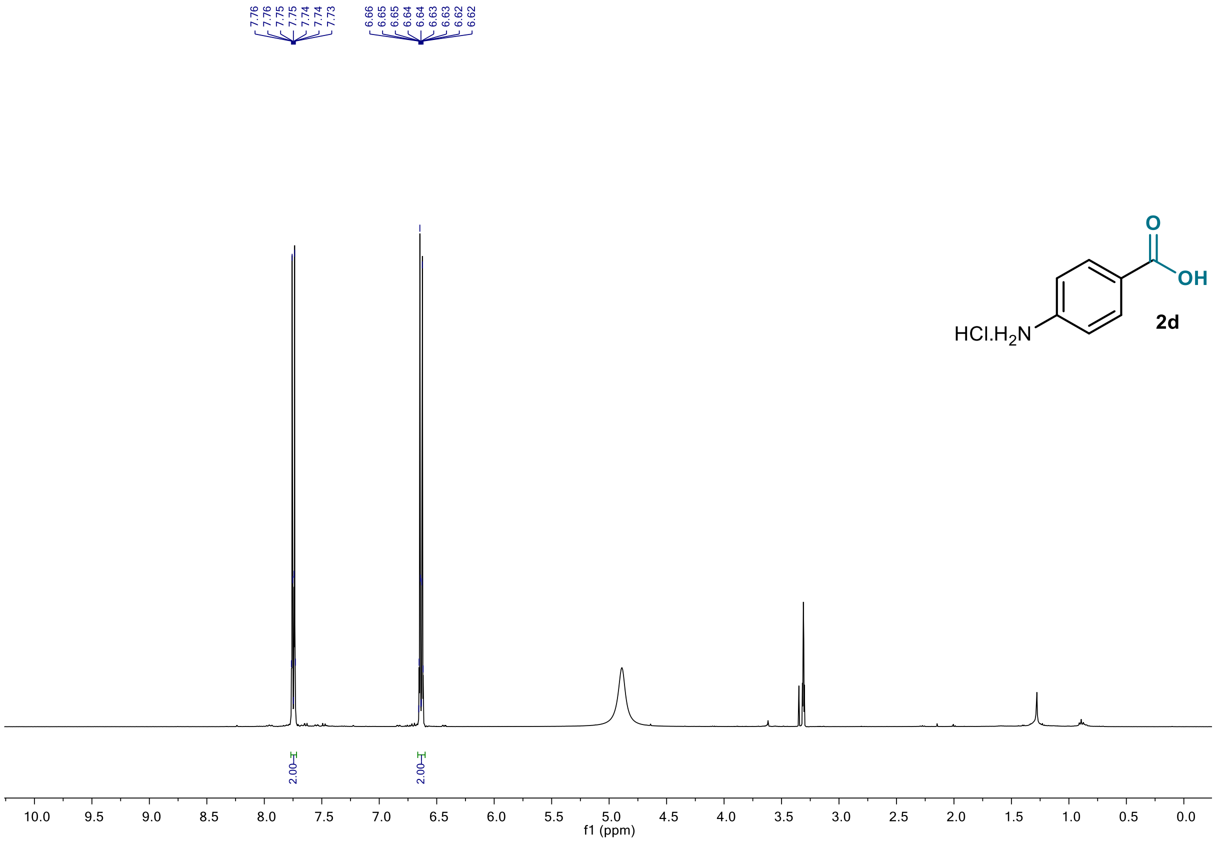

Figure S23: ^{1}H NMR (400 MHz, Methanol-*d*$_4$) spectrum of 4-aminobenzoic acid hydrochloride (**2d-HCl**).

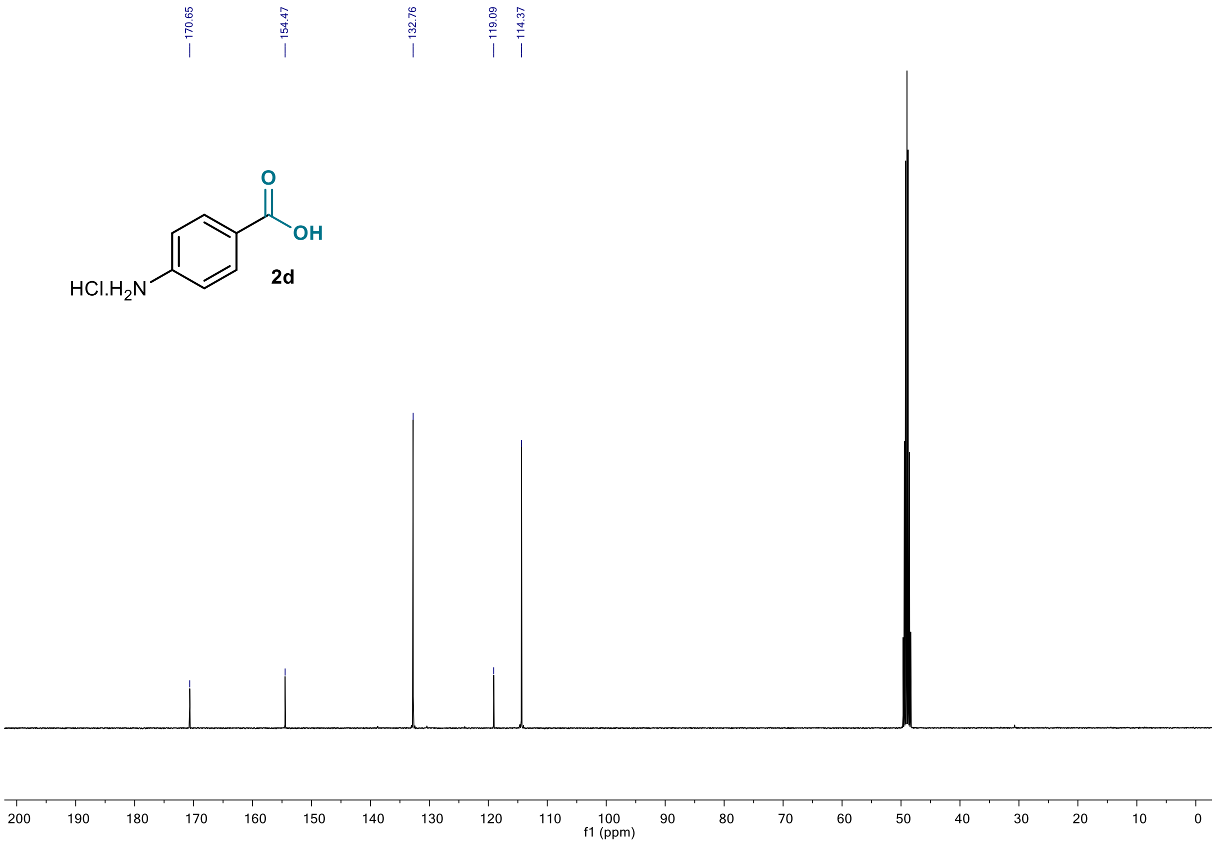

Figure S24: ^{13}C NMR (101 MHz, Methanol-*d*$_4$) spectrum of 4-Aminobenzoic acid hydrochloride (**2d-HCl**).

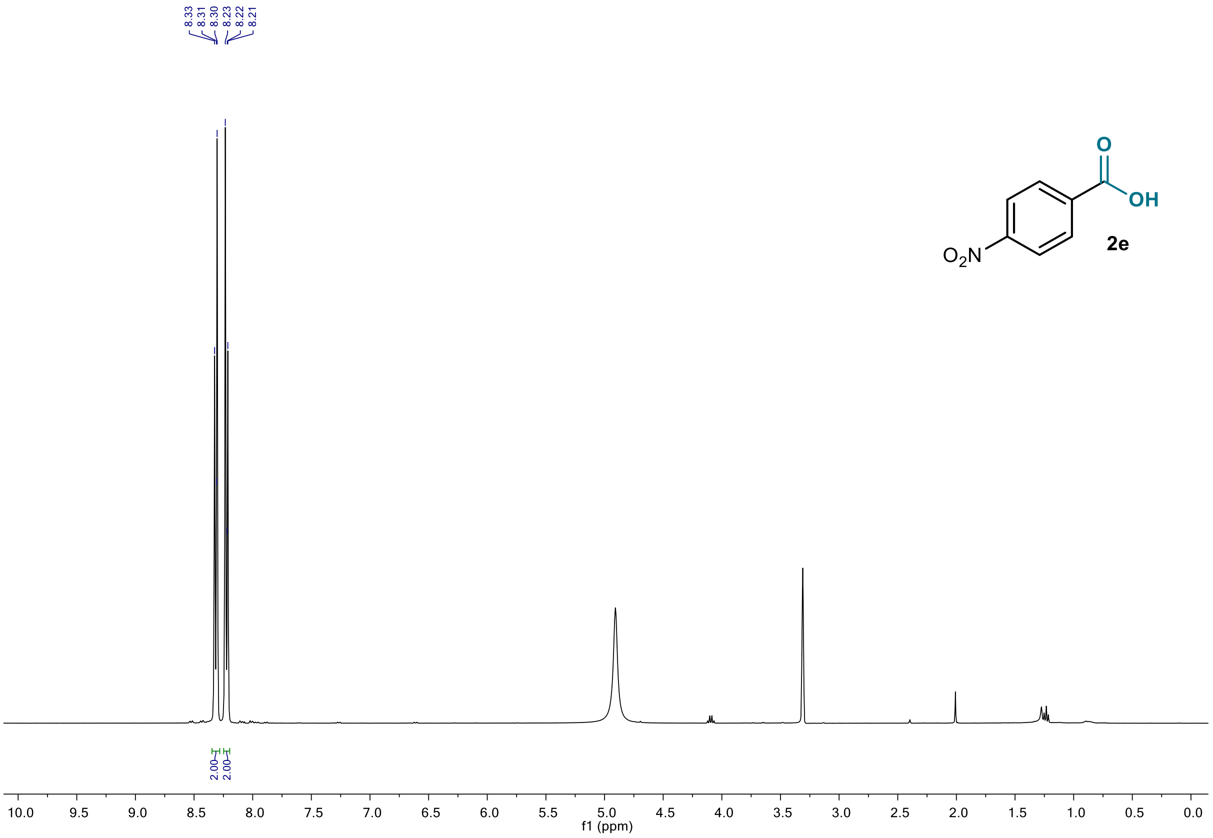

Figure S25: ^{1}H NMR (400 MHz, Methanol-d_4) spectrum of 4-nitrobenzoic acid (**2e**).

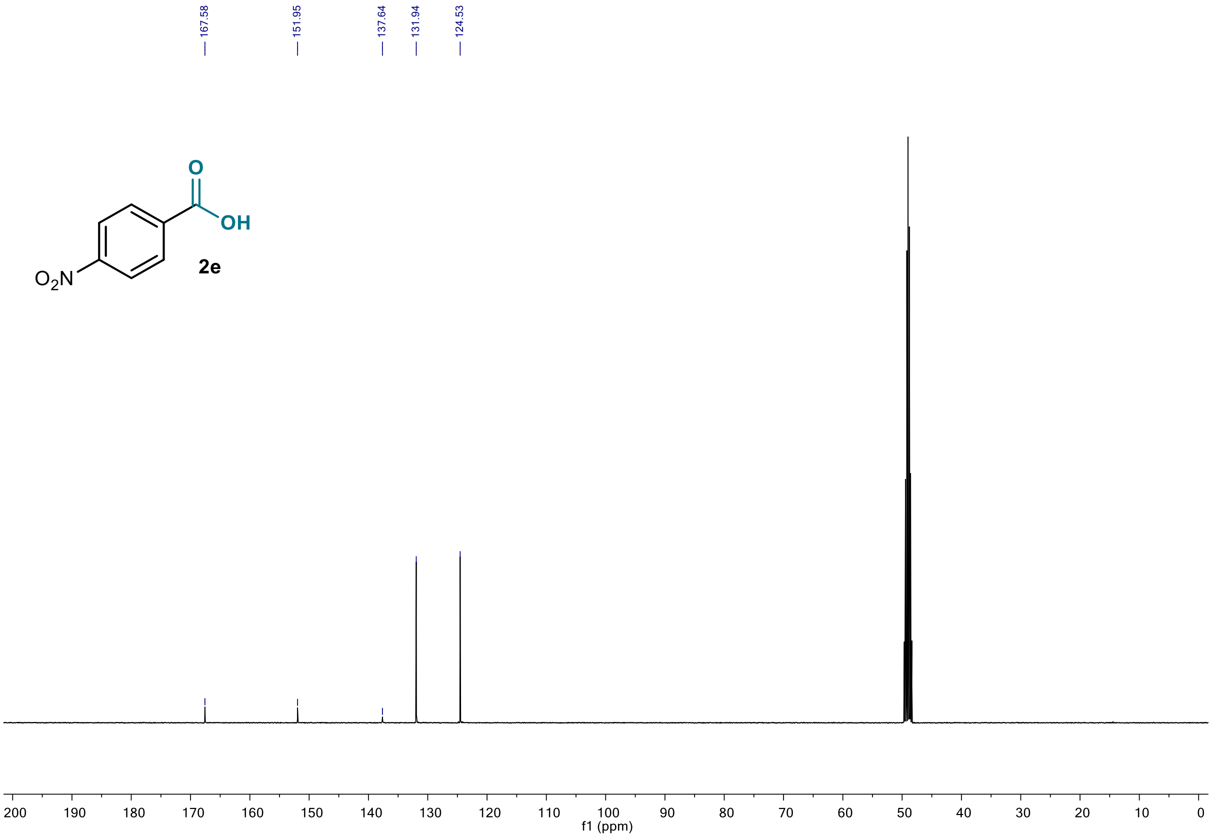

Figure S26: ^{13}C NMR (101 MHz, Methanol-d_4) spectrum of 4-nitrobenzoic acid (**2e**).

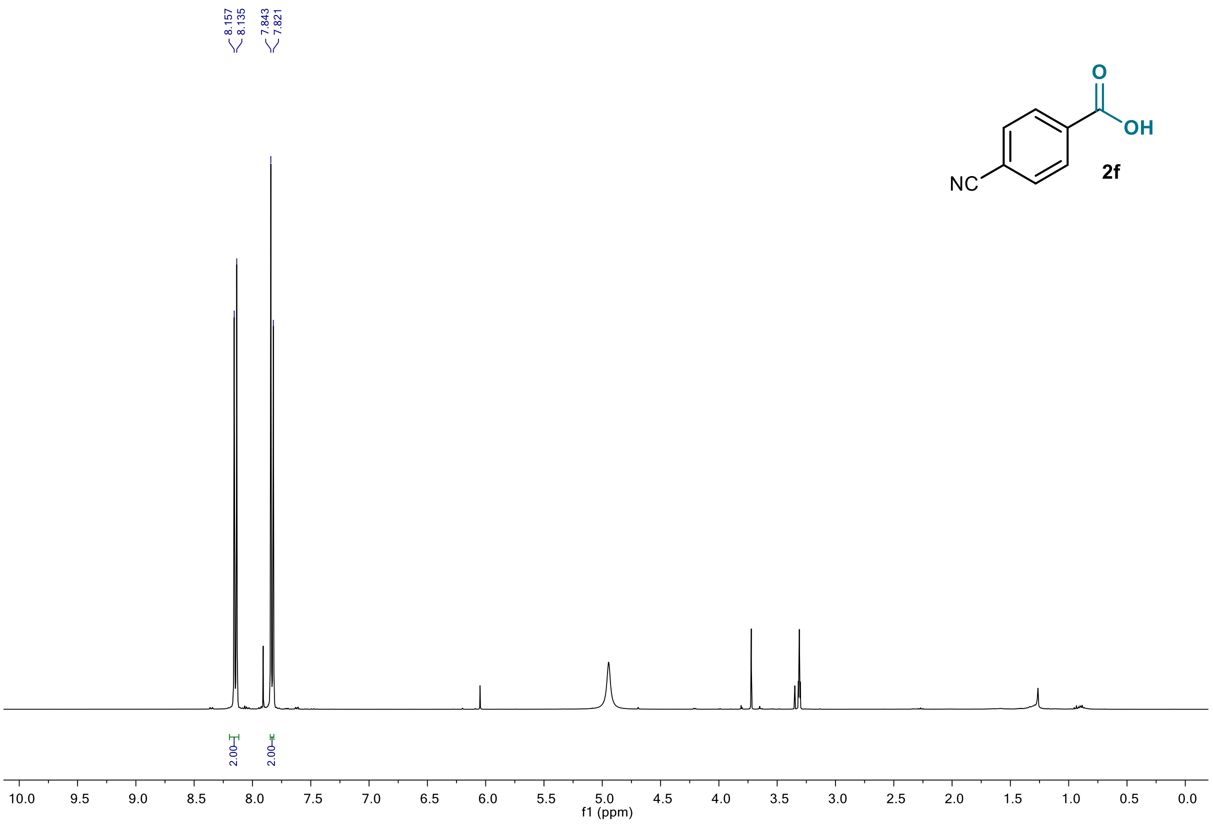

Figure S27: ^{1}H NMR (400 MHz, Methanol-d_4) spectrum of 4-cyanobenzoic acid (**2f**).

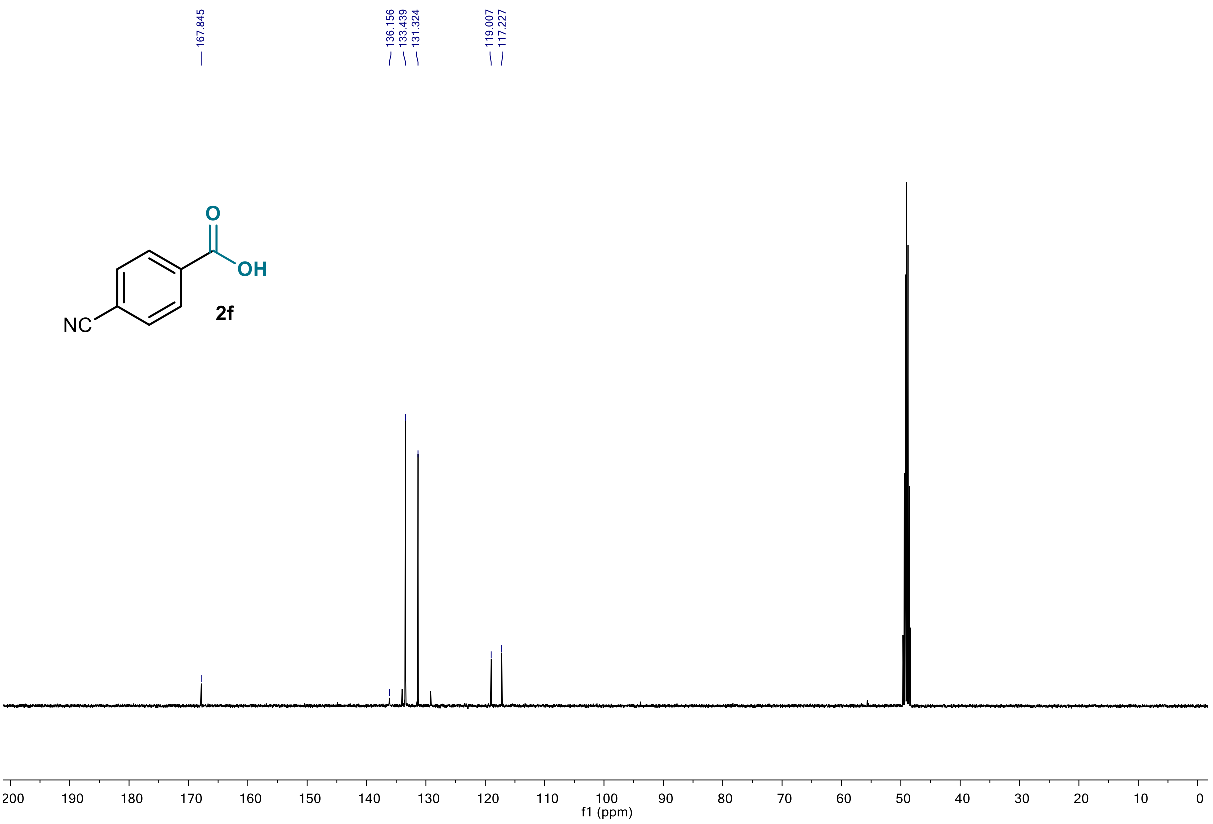

Figure S28: ^{13}C NMR (101 MHz, Methanol-d_4) spectrum of 4-cyanobenzoic acid (**2f**).

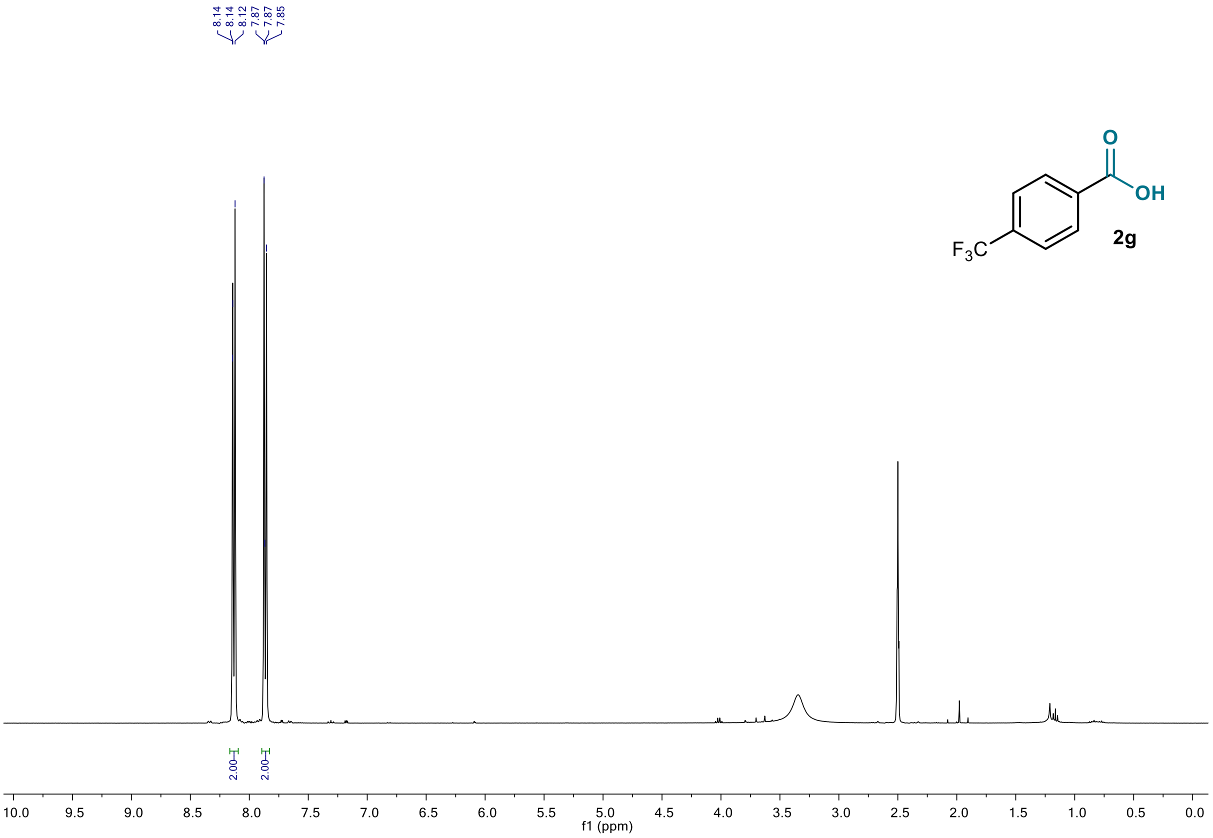

Figure S29: ^{1}H NMR (400 MHz, Methanol-d_4) spectrum of 4-(trifluoromethyl)benzoic acid (**2g**).

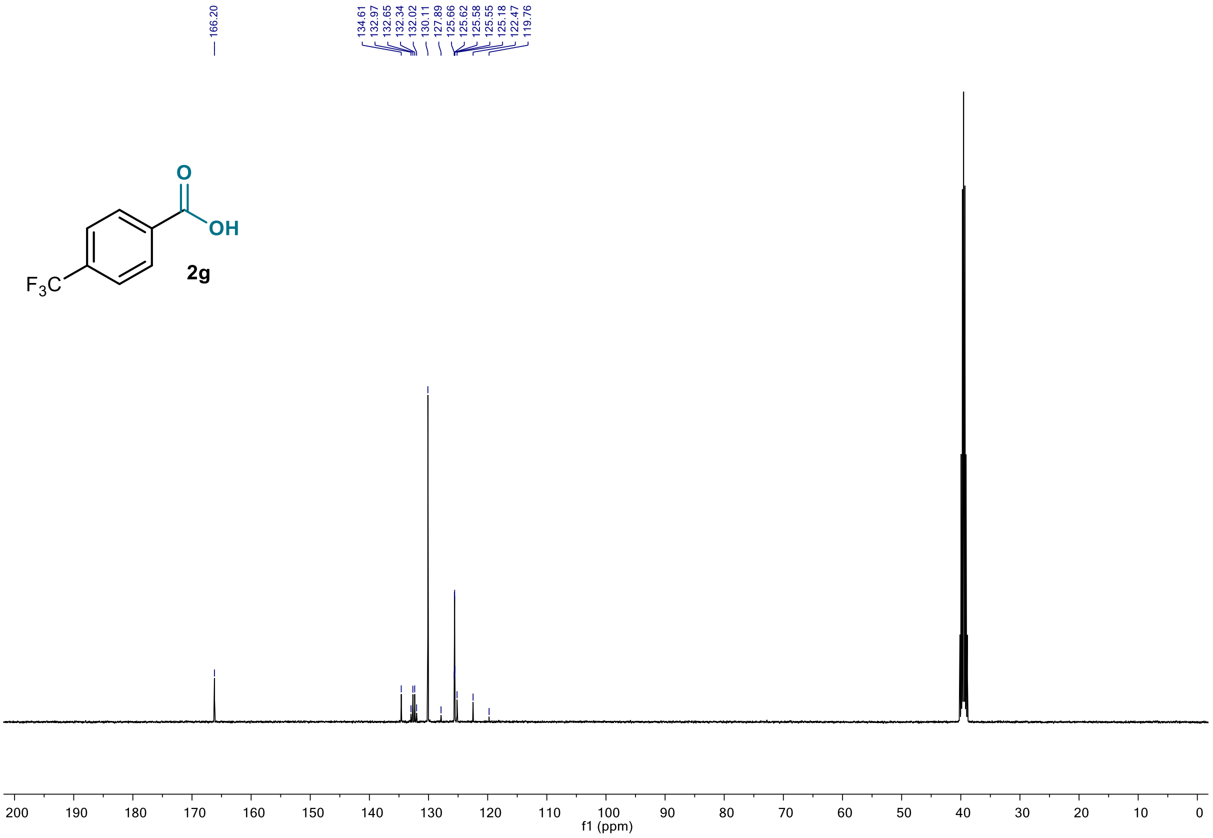

Figure S30: ^{13}C NMR (101 MHz, Methanol-d_4) spectrum of 4-(trifluoromethyl)benzoic acid (**2g**).

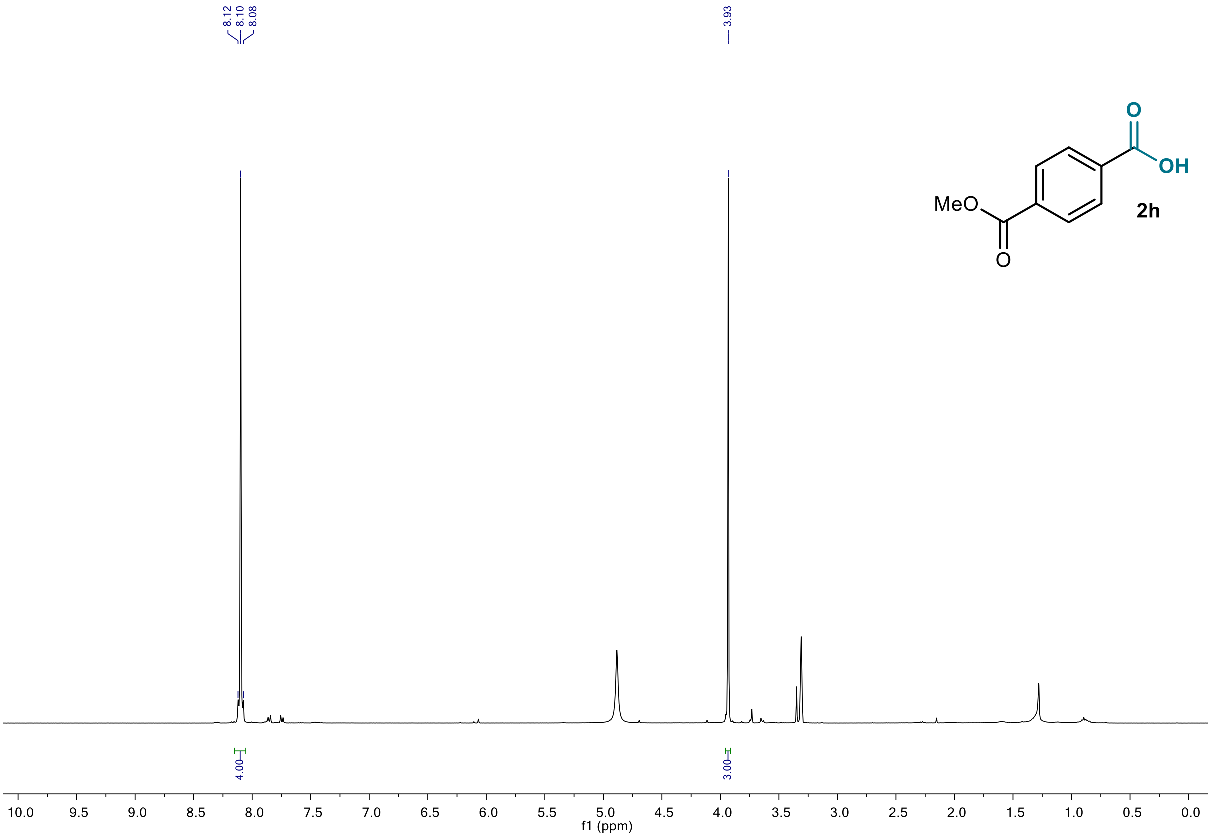

Figure S31: ^{1}H NMR (400 MHz, Methanol-d_4) spectrum of 4-(methoxycarbonyl)benzoic acid (**2h**).

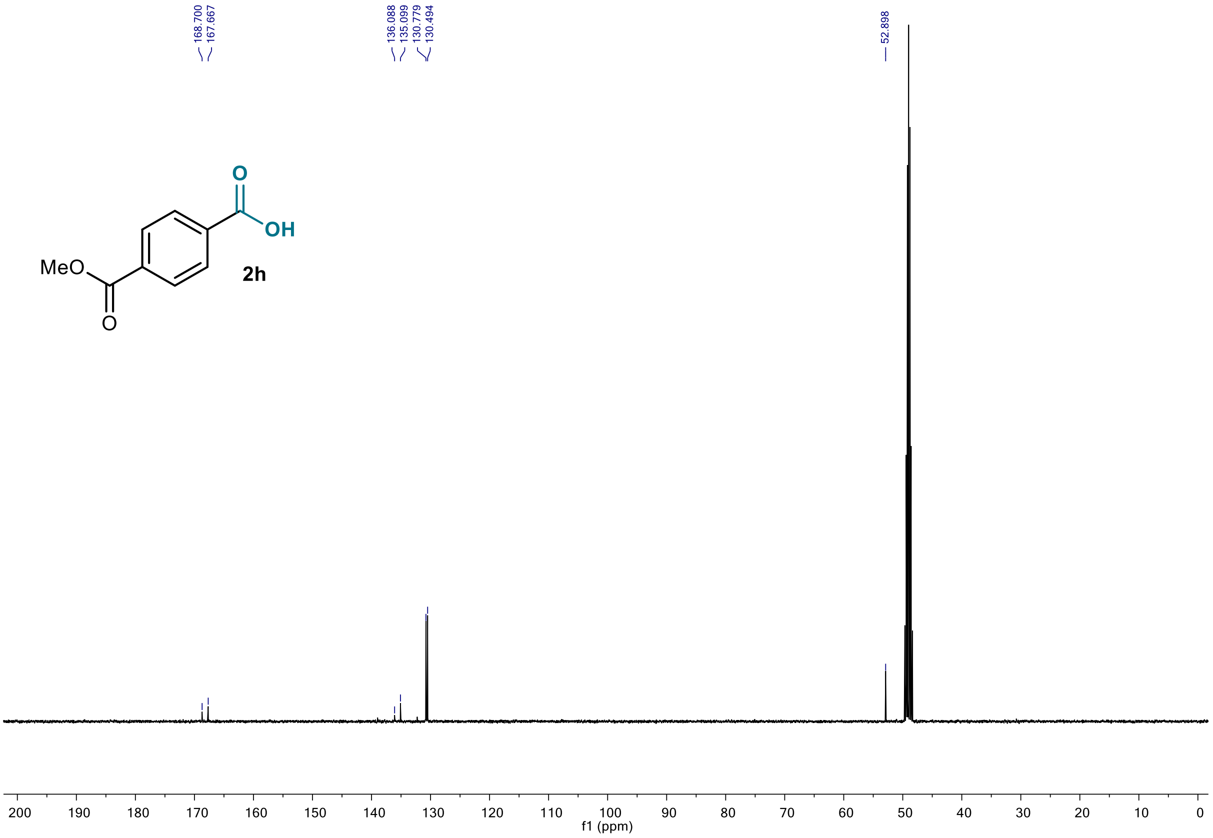

Figure S32: ^{13}C NMR (101 MHz, Methanol-d_4) spectrum of 4-(methoxycarbonyl)benzoic acid (**2h**).

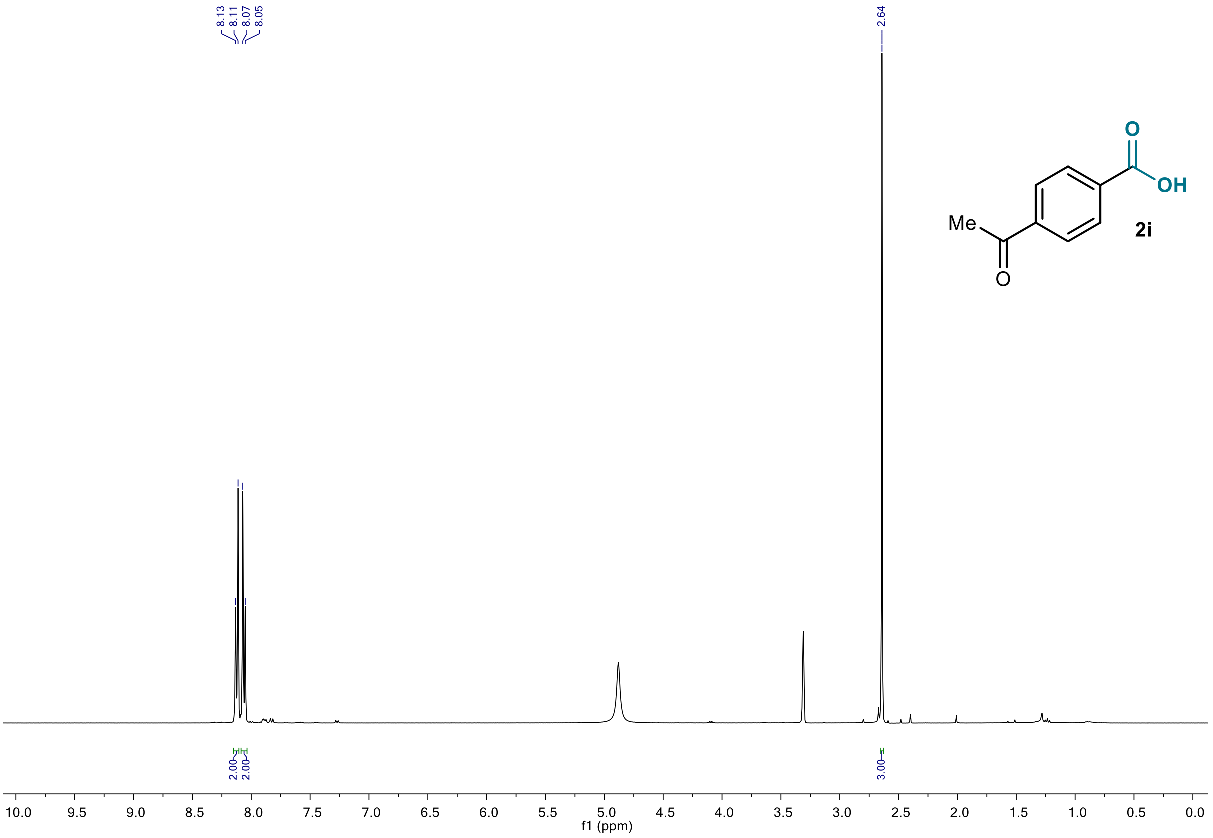

Figure S33: ^{1}H NMR (400 MHz, Methanol-d_4) spectrum of 4-acetylbenzoic acid (**2i**).

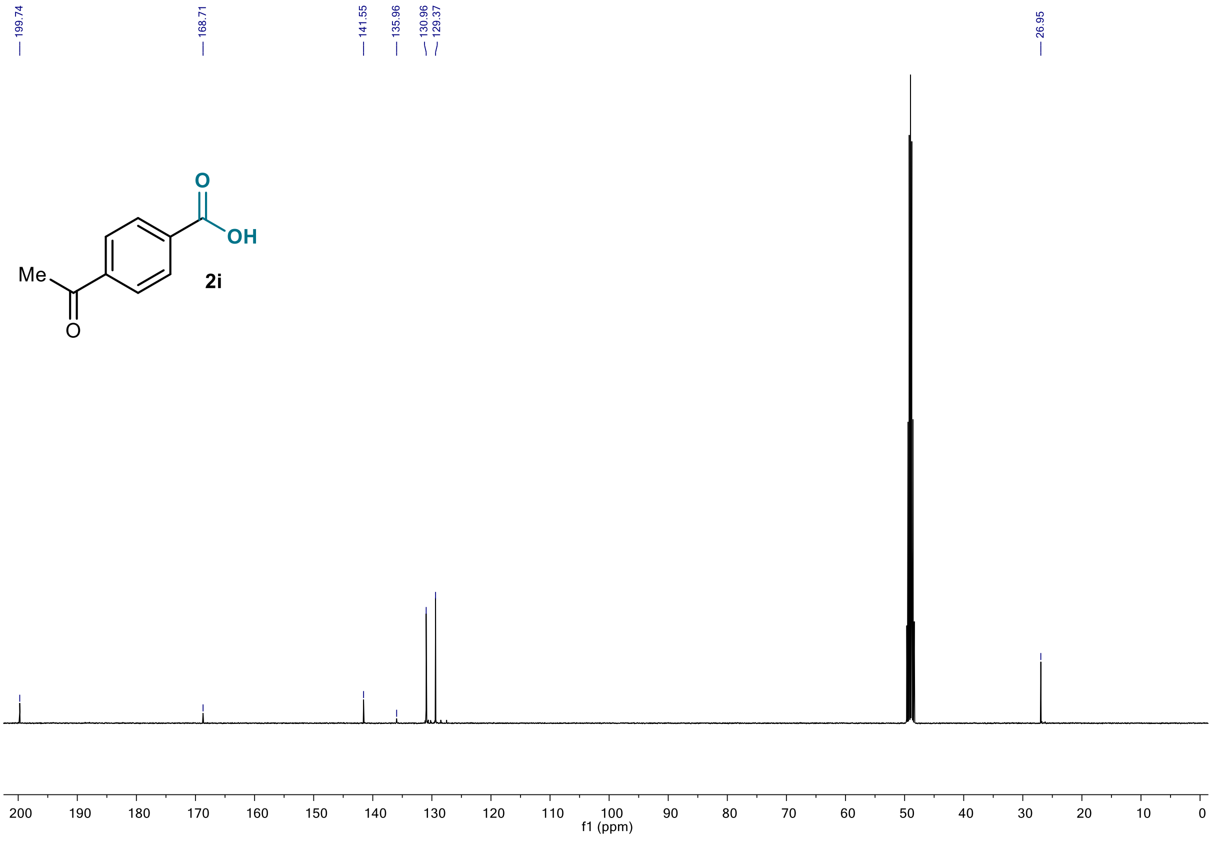

Figure S34: ^{13}C NMR (101 MHz, Methanol-d_4) spectrum of 4-acetylbenzoic acid (**2i**).

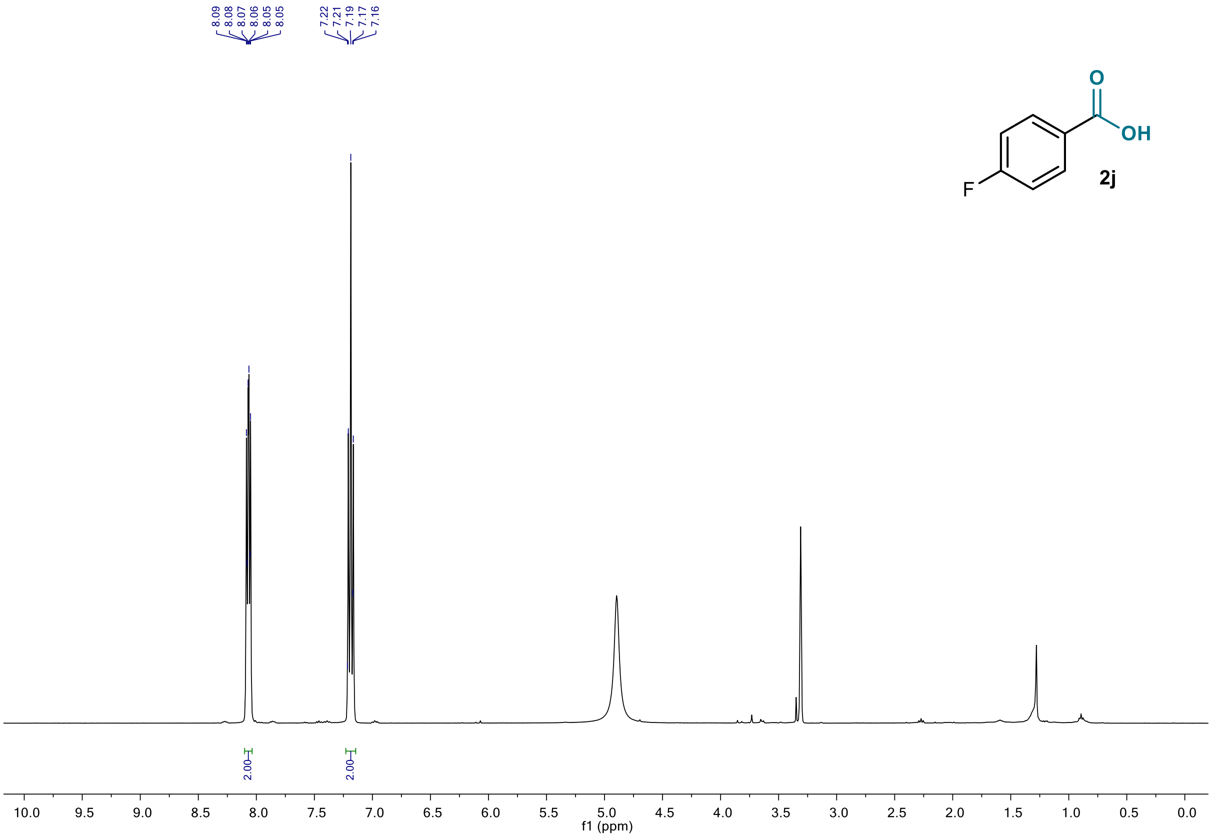

Figure S35: ^{1}H NMR (400 MHz, Methanol-d_4) spectrum of 4-fluorobenzoic acid (**2j**).

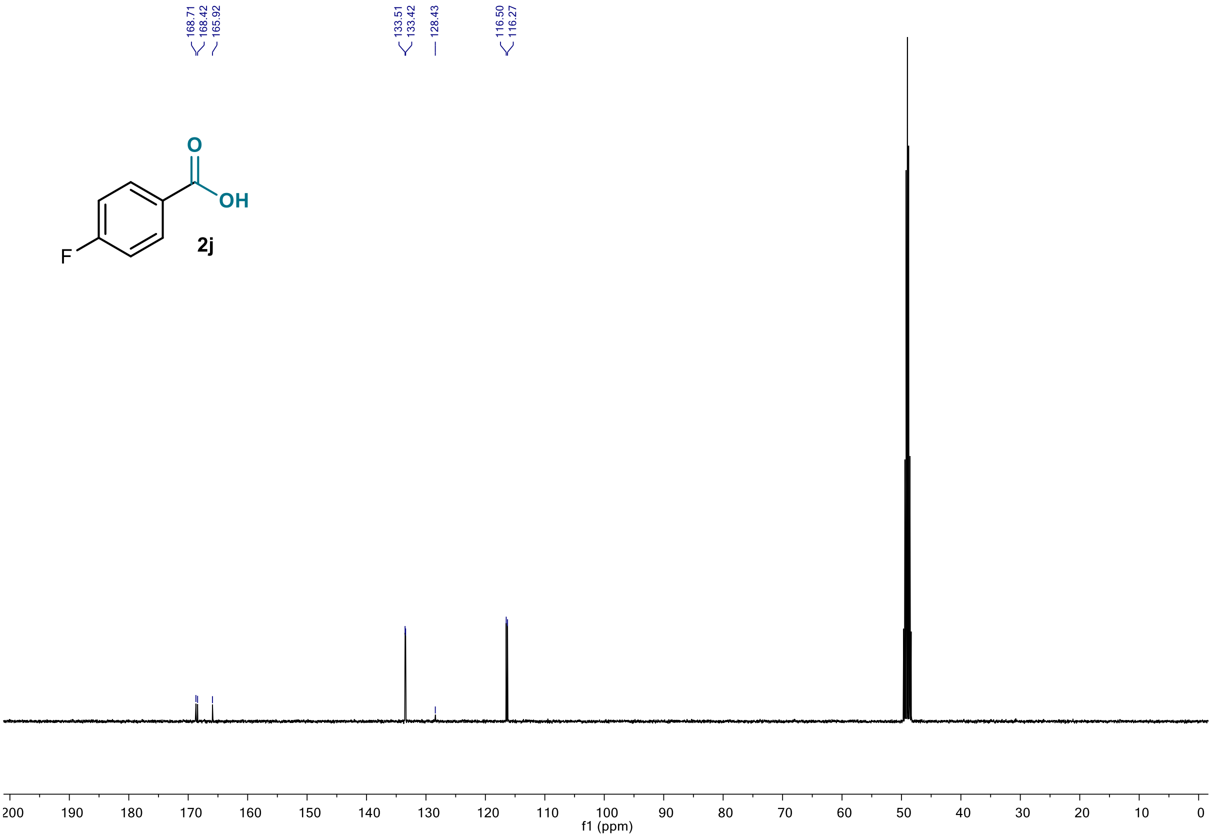

Figure S36: ^{13}C NMR (101 MHz, Methanol-d_4) spectrum of 4-fluorobenzoic acid (**2j**).

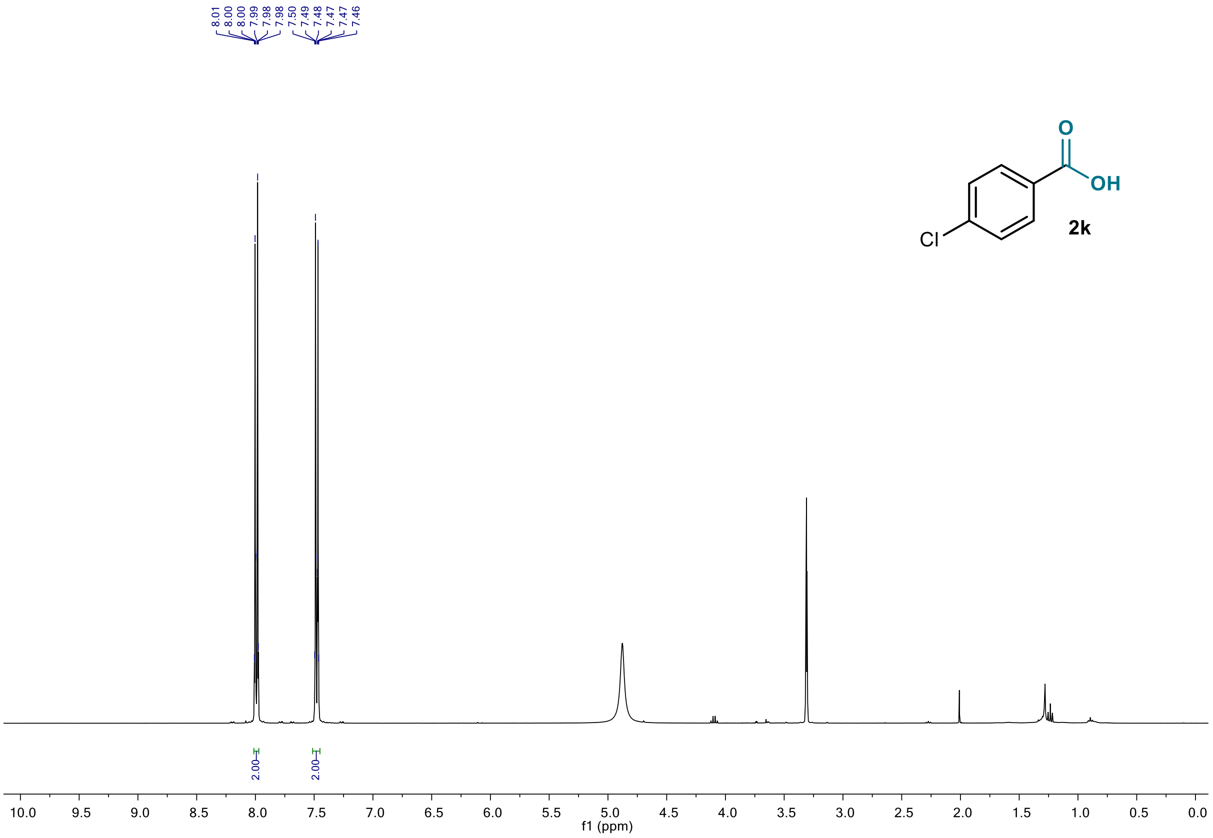

Figure S37: ^{1}H NMR (400 MHz, Methanol-d_4) spectrum of 4-chlorobenzoic acid (**2k**).

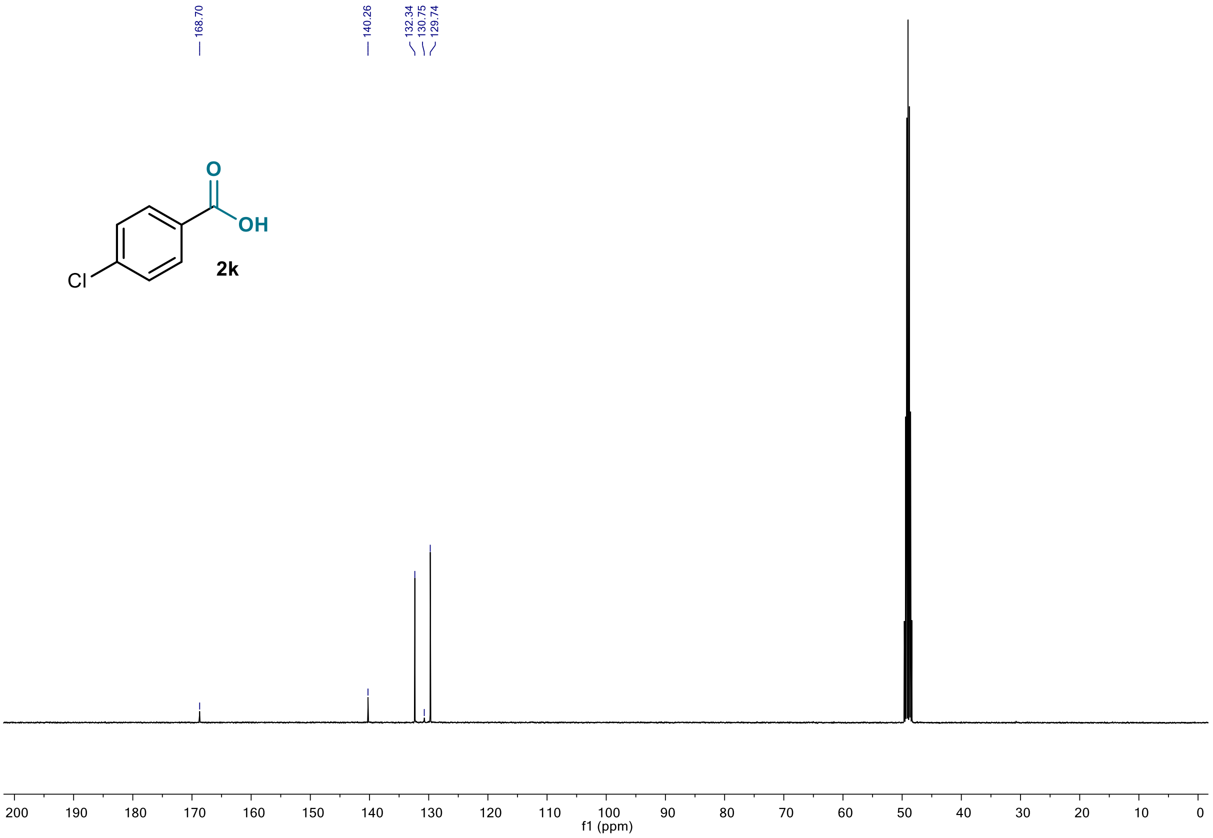

Figure S38: ^{13}C NMR (101 MHz, Methanol-d_4) spectrum of 4-chlorobenzoic acid (**2k**).

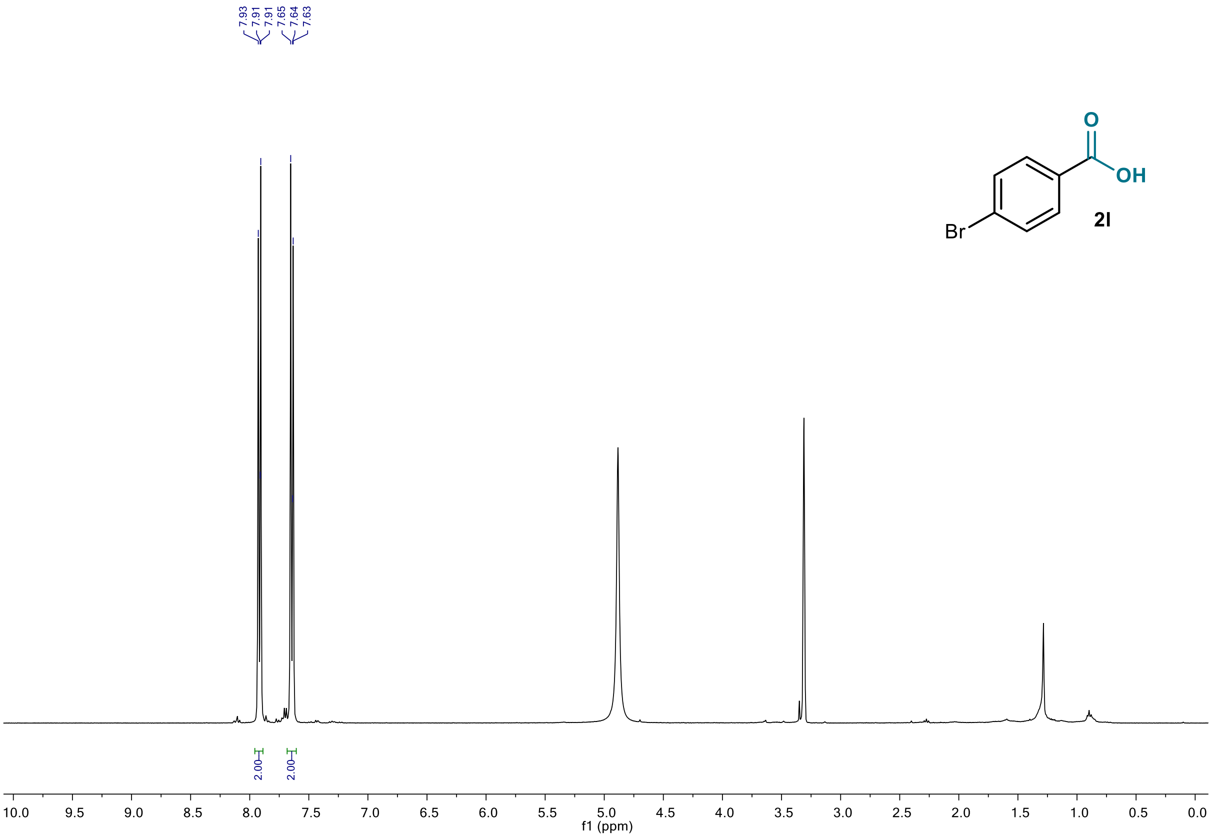

Figure S39: ^{1}H NMR (400 MHz, Methanol-d_4) spectrum of 4-bromobenzoic acid (**2l**).

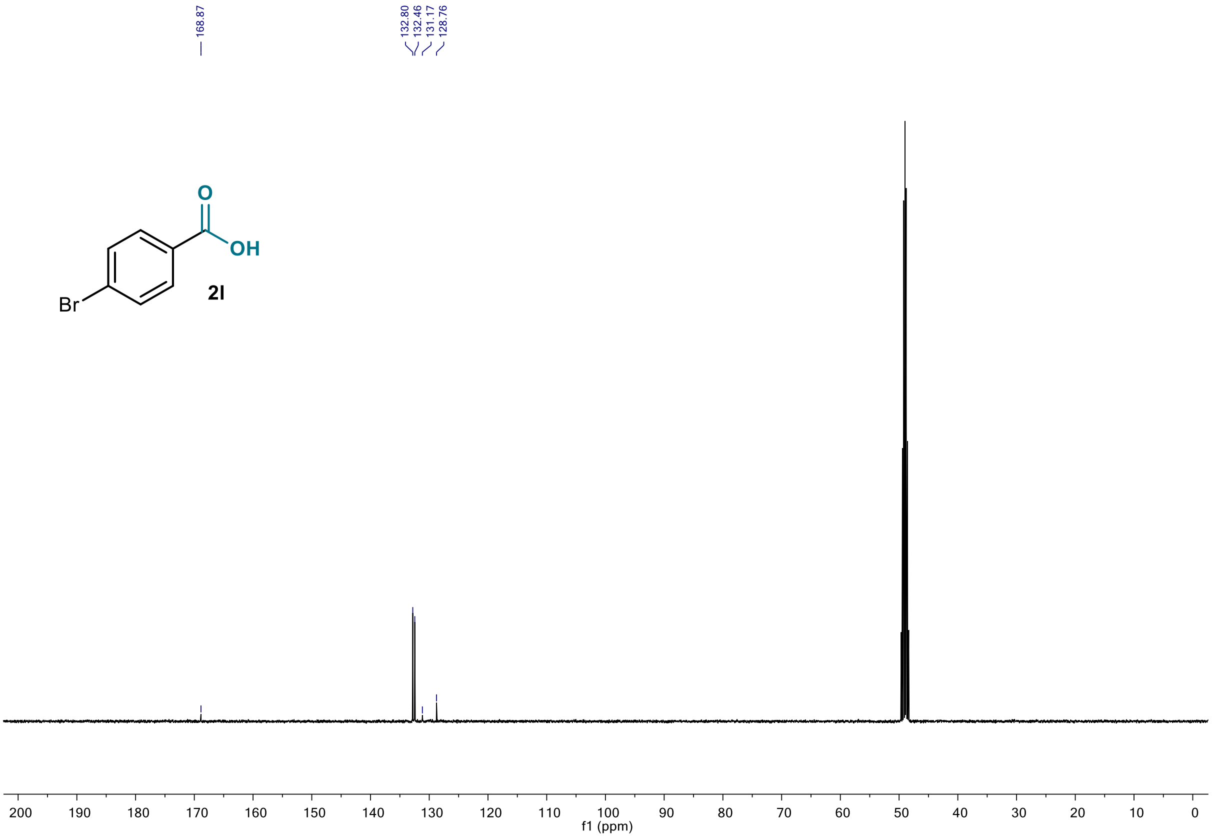

Figure S40: ^{13}C NMR (101MHz, Methanol-d_4) spectrum of 4-bromobenzoic acid (**2l**).

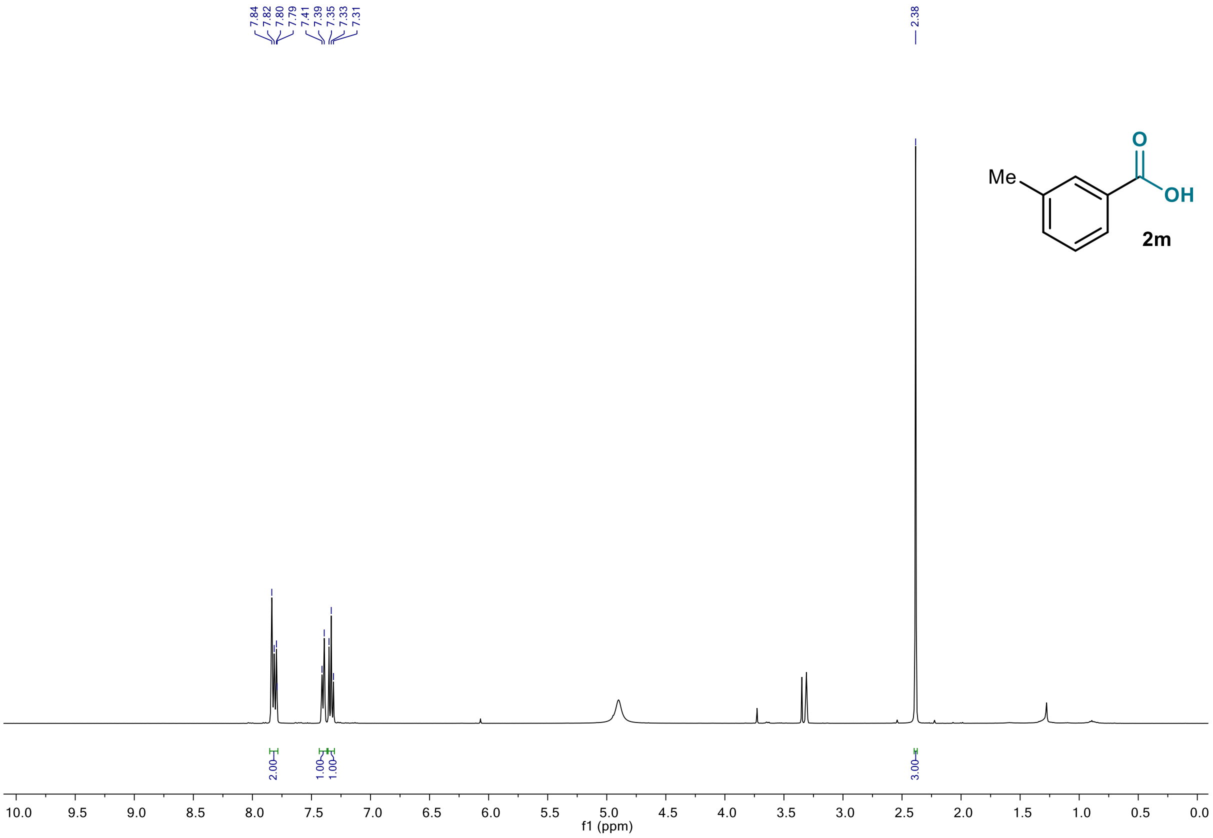

Figure S41: ^{1}H NMR (400 MHz, Methanol-d_4) spectrum of 3-methylbenzoic acid (**2m**).

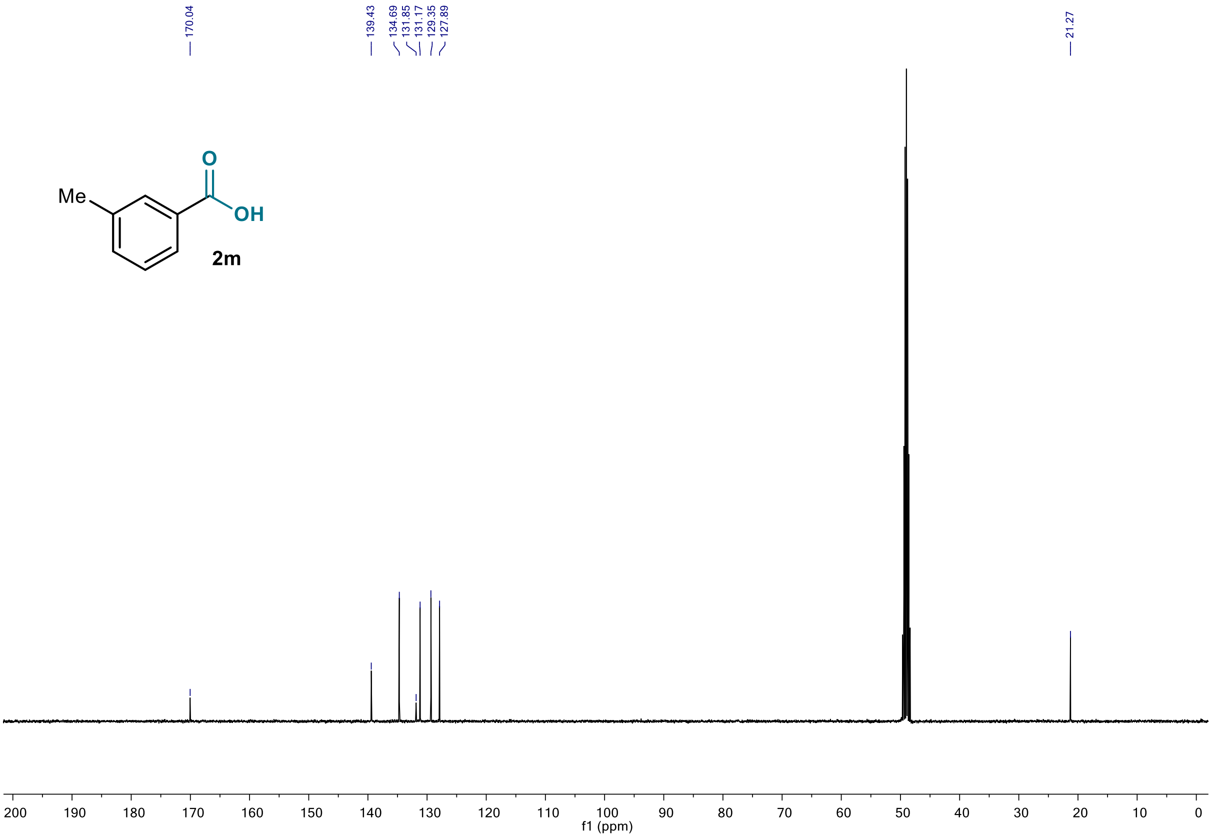

Figure S42: ^{13}C NMR (101 MHz, Methanol-d_4) spectrum of 3-methylbenzoic acid (**2m**).

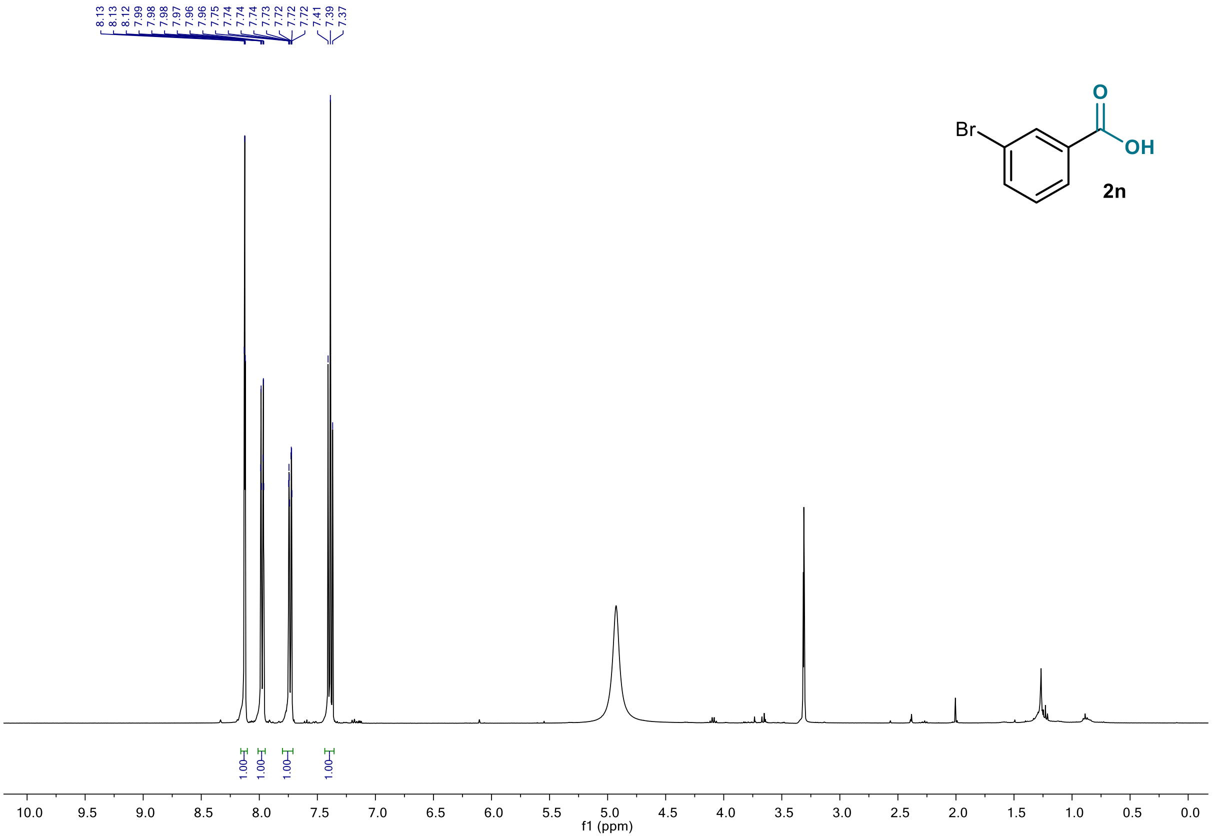

Figure S43: ^{1}H NMR (400 MHz, Methanol-d_4) spectrum of 3-bromobenzoic acid (**2n**).

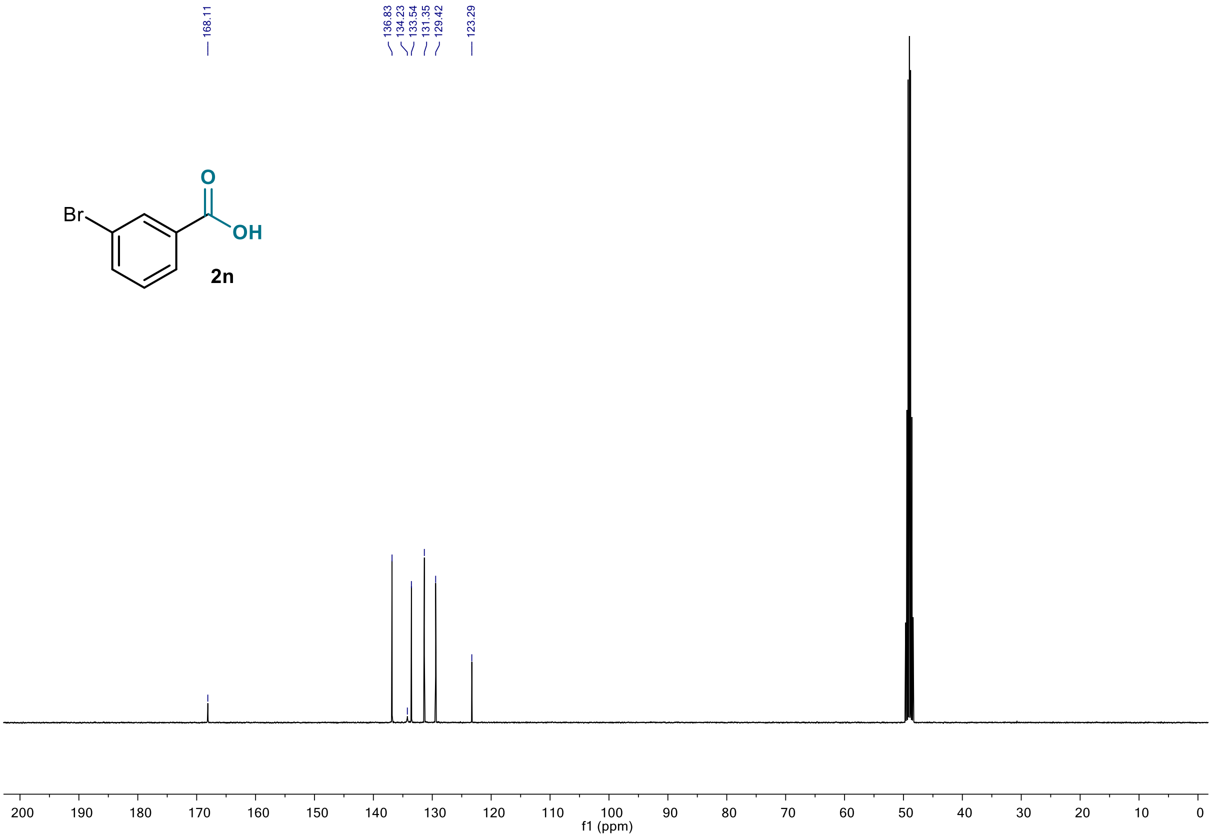

Figure S44: ^{13}C NMR (101 MHz, Methanol-d_4) spectrum of 3-bromobenzoic acid (**2n**).

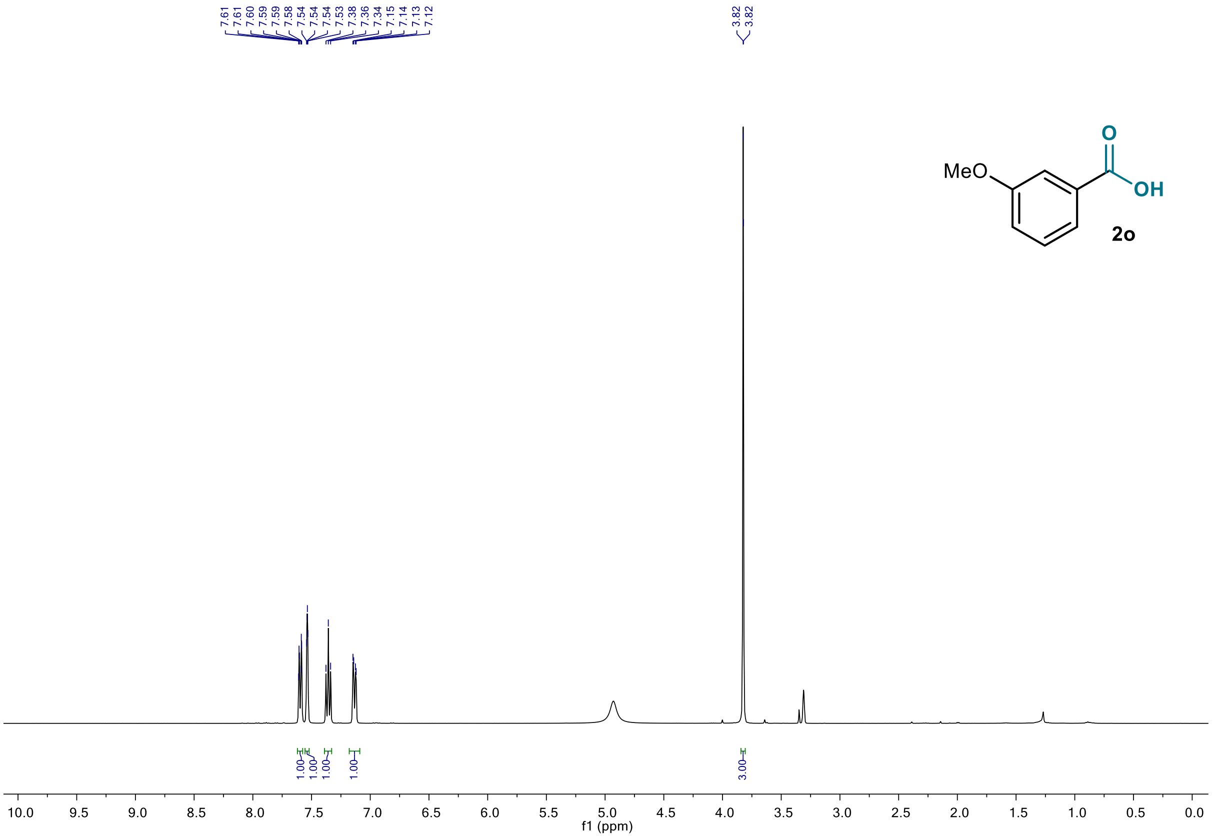

Figure S45: ^{1}H NMR (400 MHz, Methanol-d_4) spectrum of 3-methoxylbenzoic acid (**2o**).

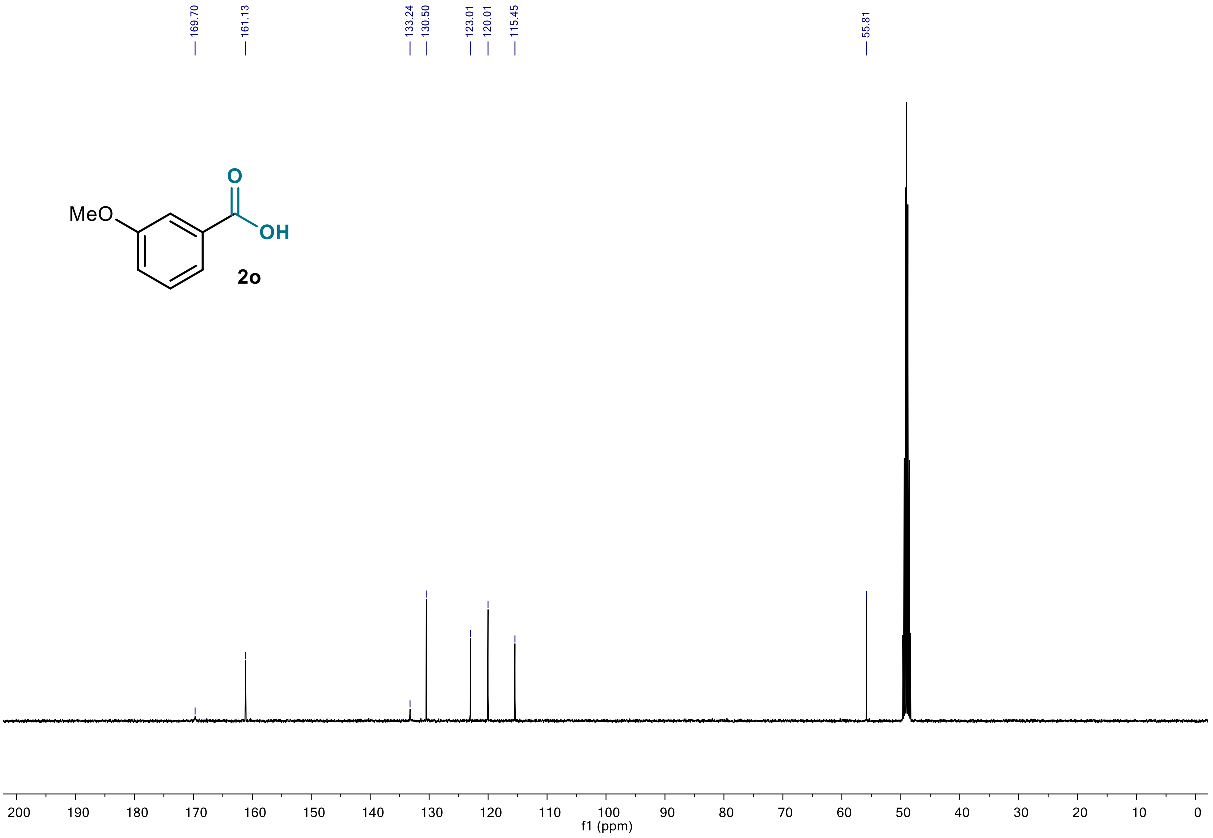

Figure S46: ^{13}C NMR (101 MHz, Methanol-d_4) spectrum of 3-methoxylbenzoic acid (**2o**).

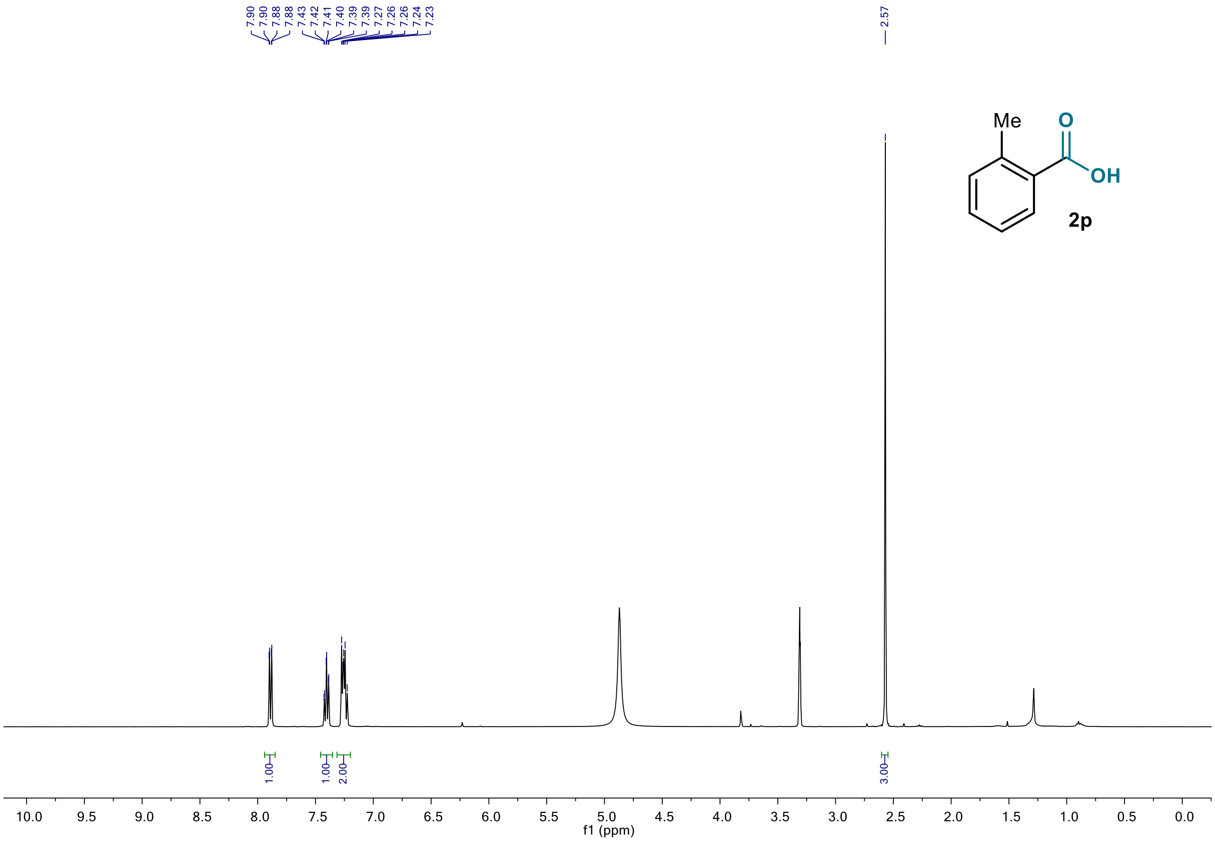

Figure S47: ^{1}H NMR (400 MHz, Methanol-d_4) spectrum of 2-methylbenzoic acid (**2p**).

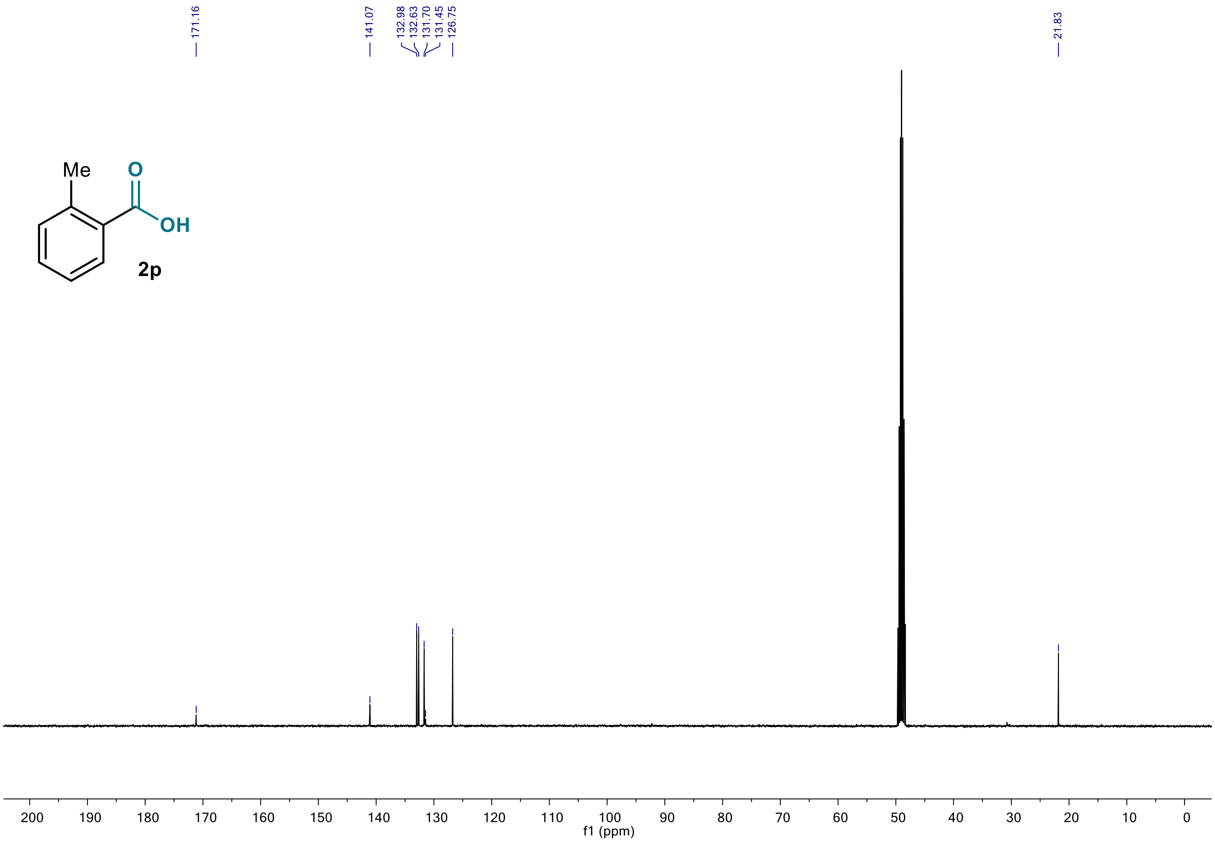

Figure S48: ^{13}C NMR (101 MHz, Methanol-d_4) spectrum of 2-methylbenzoic acid (**2p**).

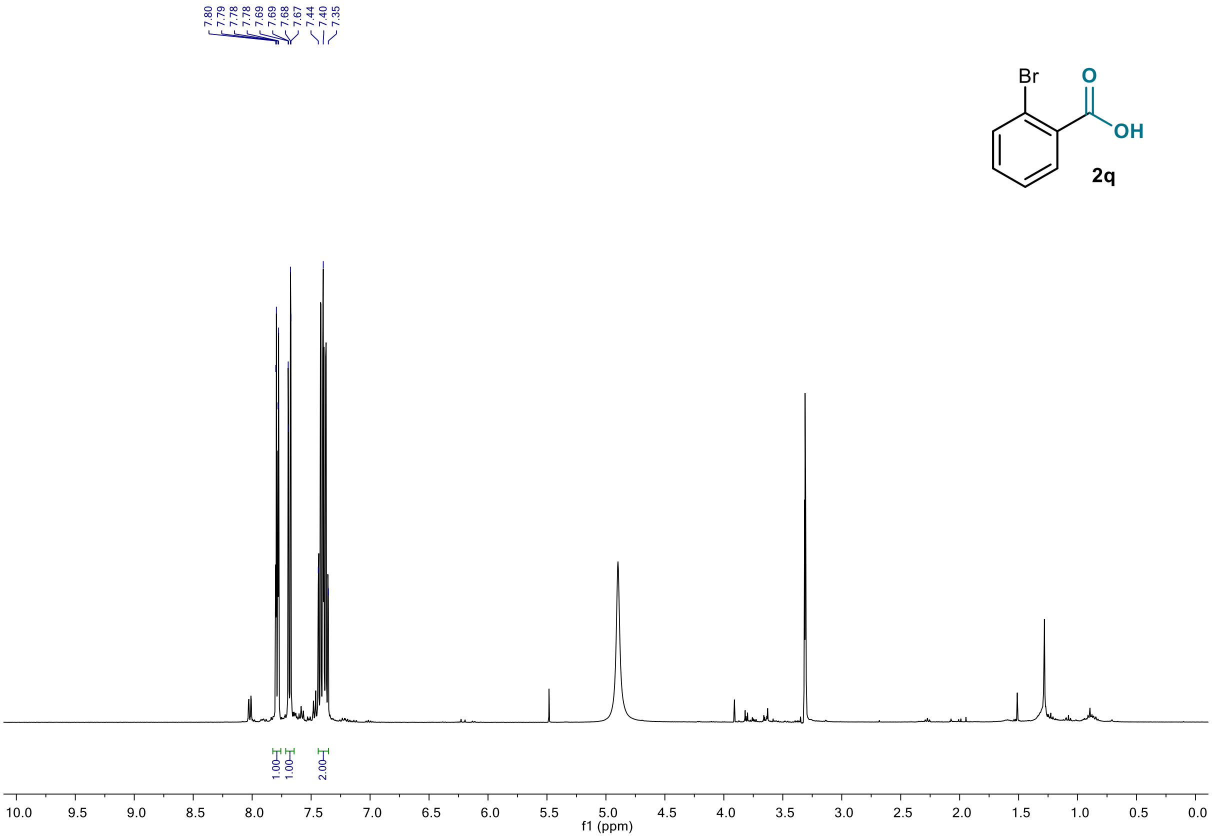

Figure S49: ^{1}H NMR (400 MHz, Methanol-d_4) spectrum of 2-bromobenzoic acid (**2q**).

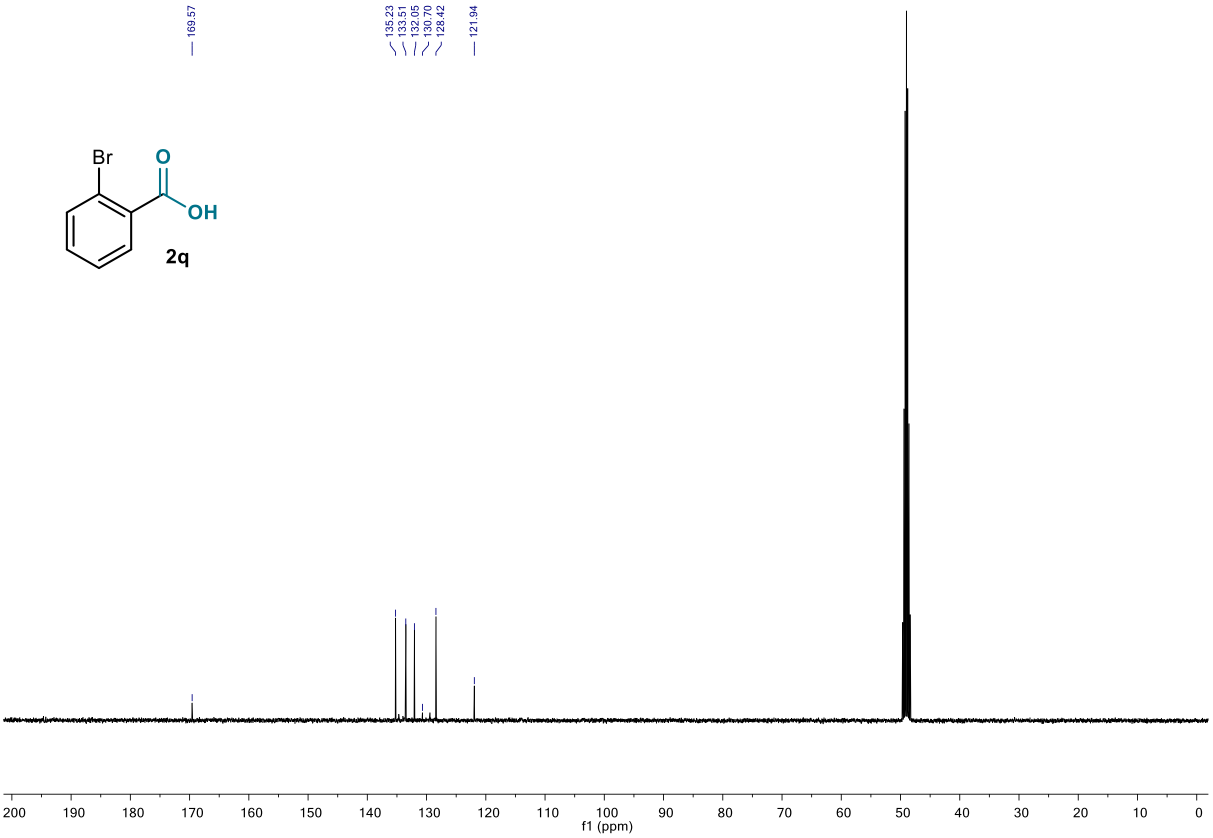

Figure S50: ^{13}C NMR (101 MHz, Methanol-d_4) spectrum of 2-bromobenzoic acid (**2q**).

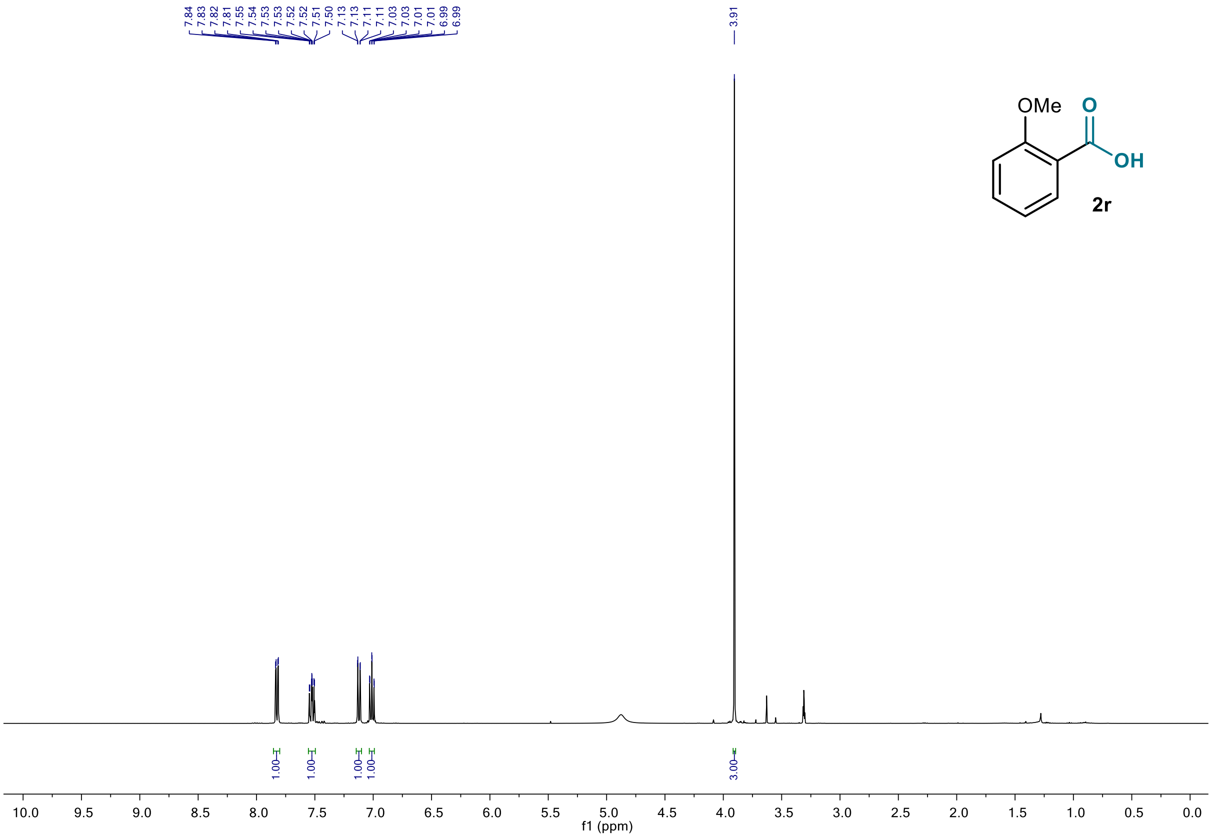

Figure S51: ^{1}H NMR (400 MHz, Methanol-d_4) spectrum of 2-methoxylbenzoic acid (**2r**).

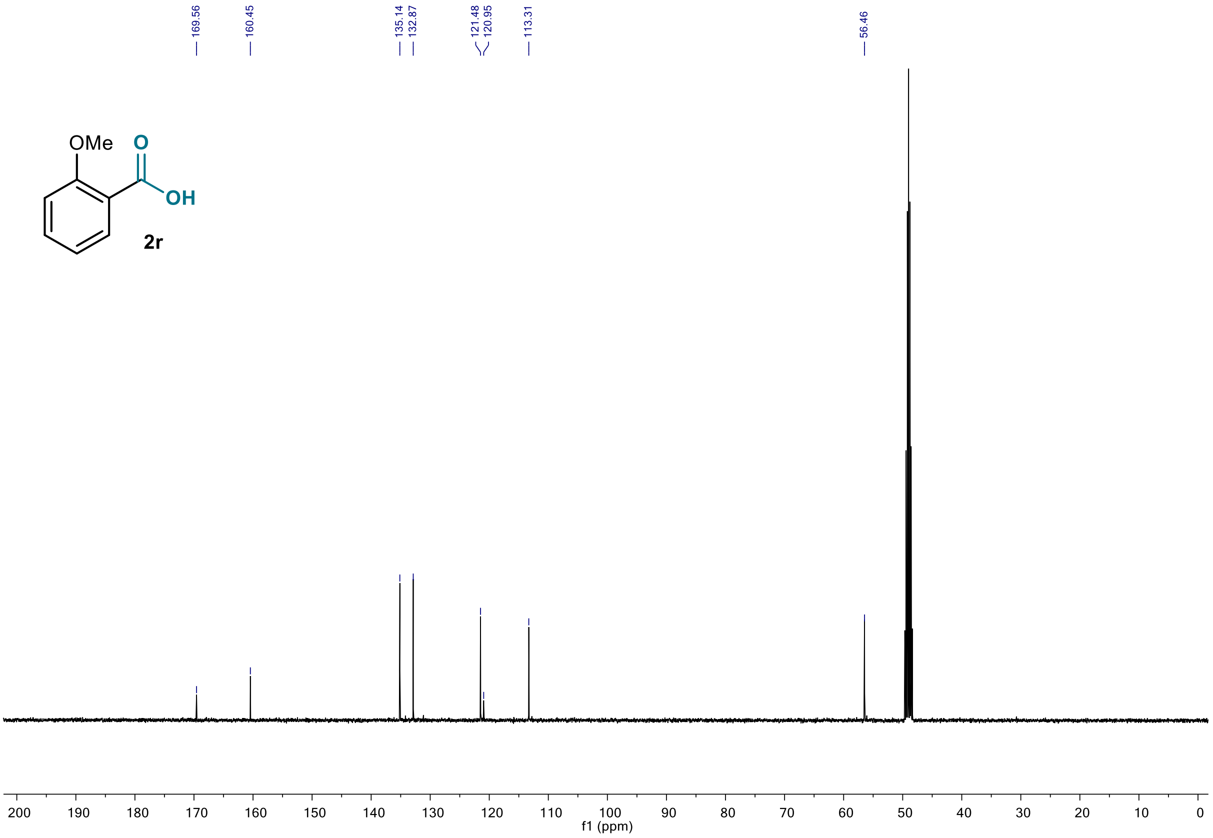

Figure S52: ^{13}C NMR (101 MHz, Methanol-d_4) spectrum of 2-methoxylbenzoic acid (**2r**).

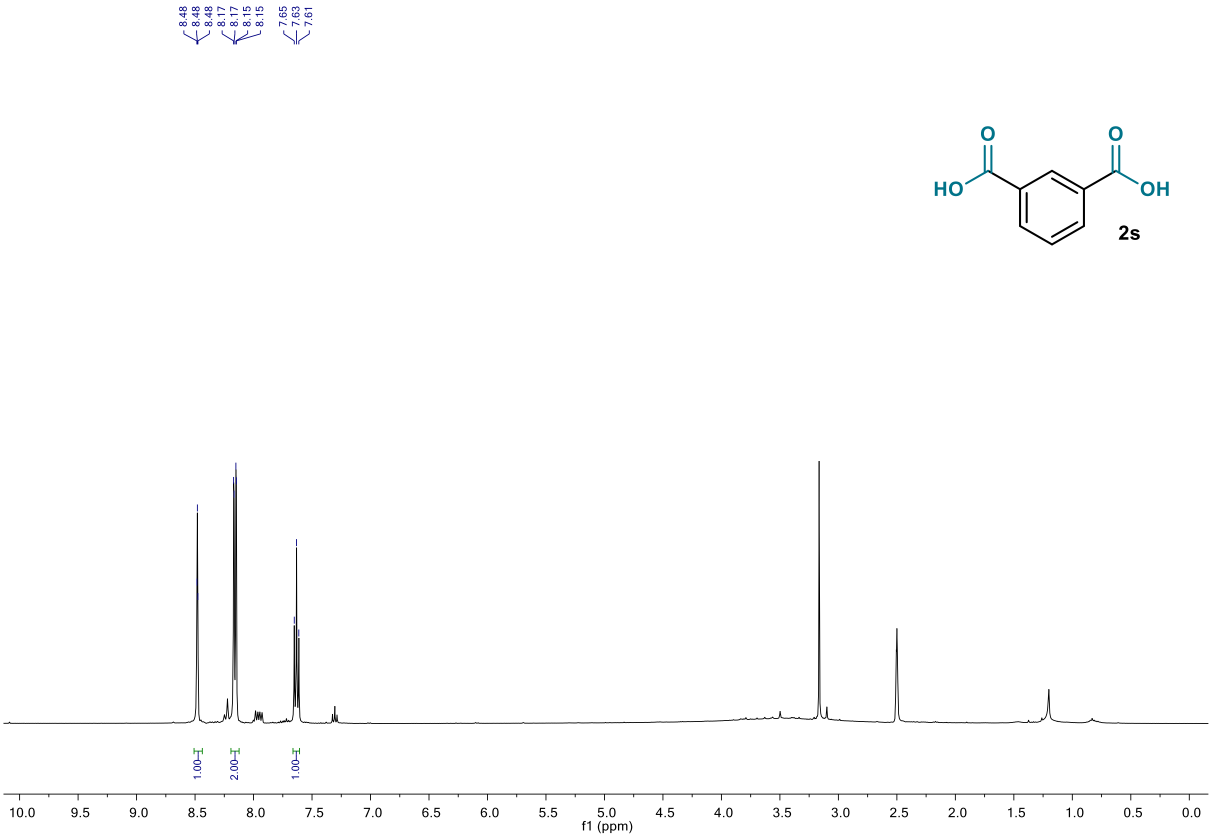

Figure S53: ^{1}H NMR (400 MHz, DMSO-d_6) spectrum of isophthalic acid (**2s**).

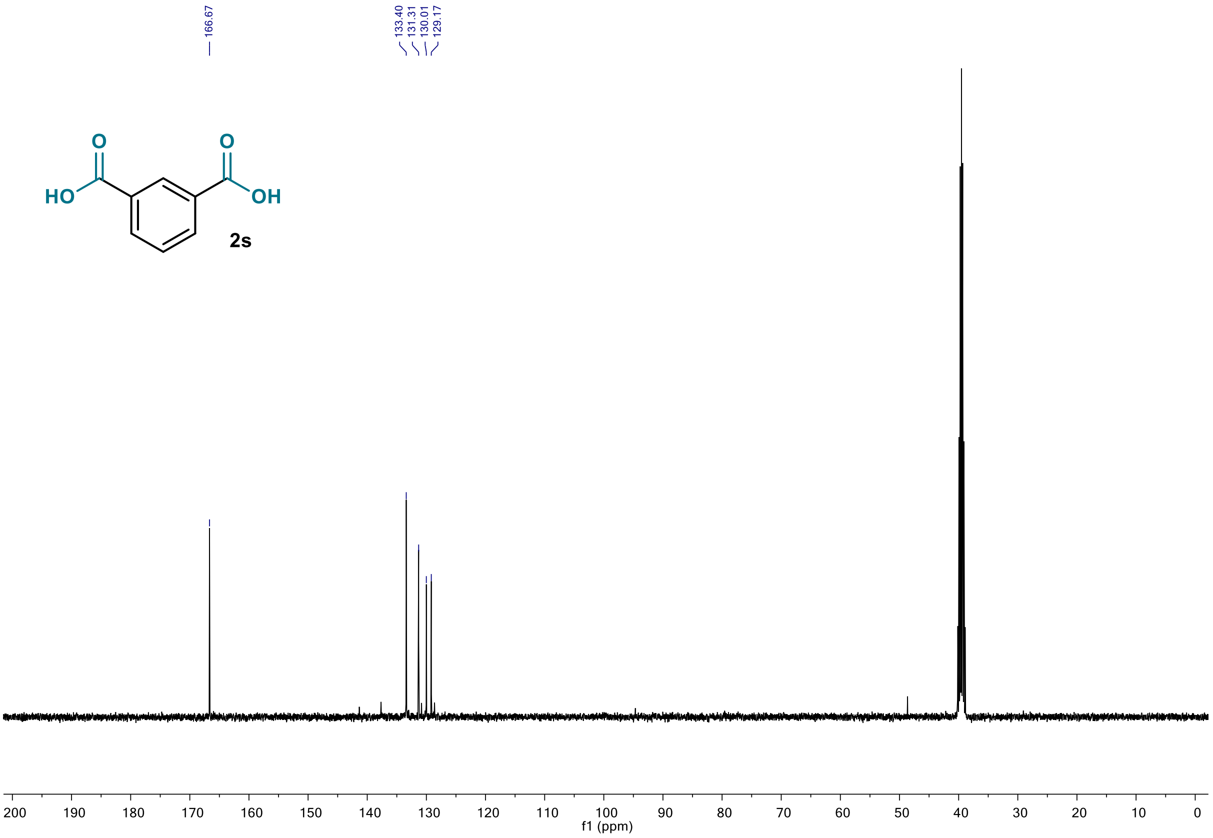

Figure S54: ^{13}C NMR (101 MHz, DMSO-d_6) spectrum of isophthalic acid (**2s**).

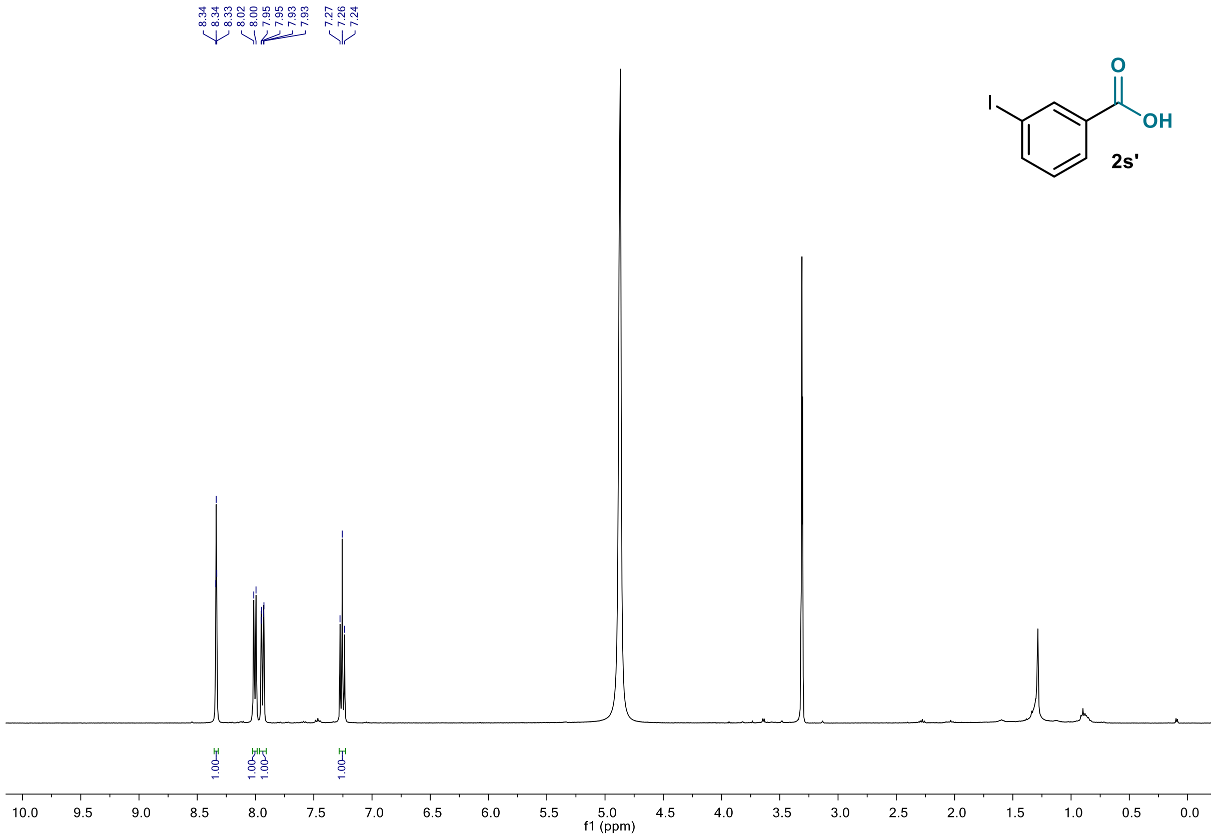

Figure S55: ^{1}H NMR (400 MHz, Methanol-d_4) spectrum of 3-iodobenzoic acid (**2s'**).

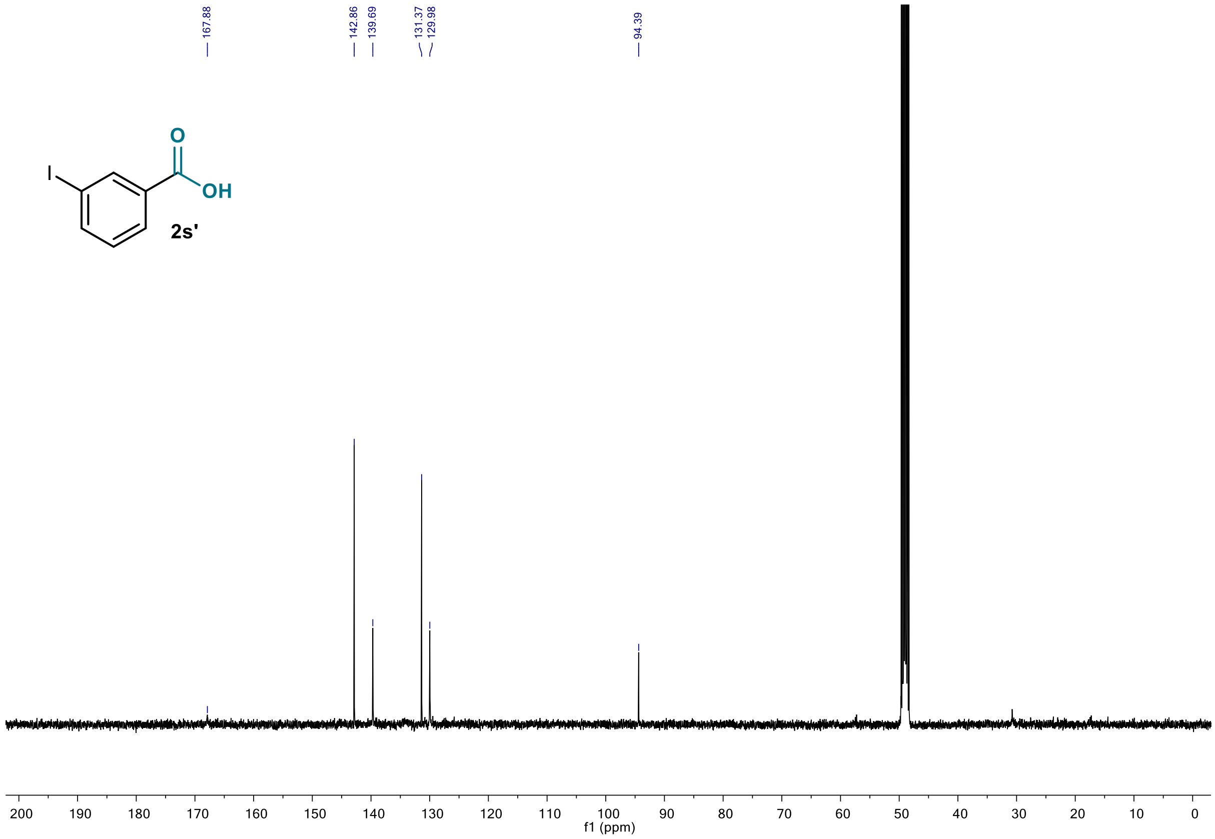

Figure S56: ^{13}C NMR (101 MHz, Methanol-d_4) spectrum of 3-iodobenzoic acid (**2s'**).

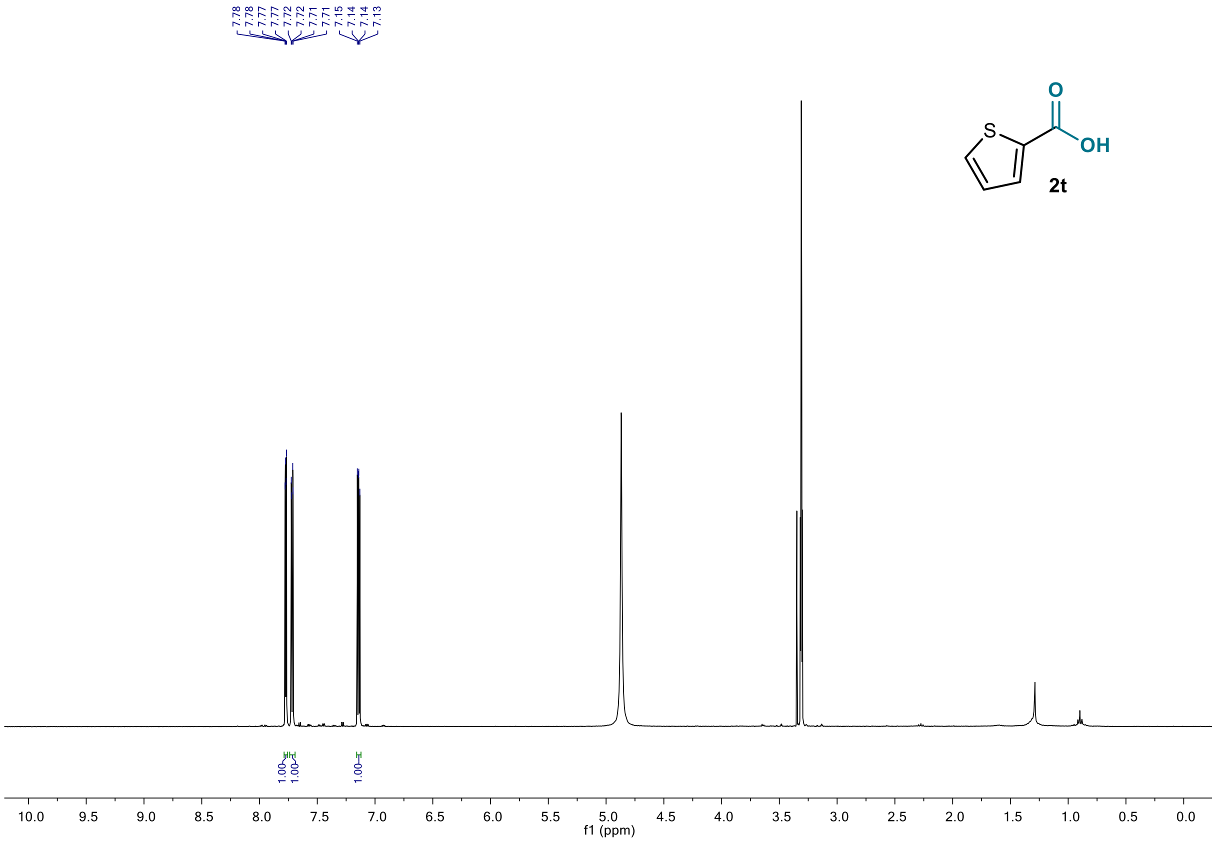

Figure S57: ^{1}H NMR (400 MHz, Methanol-d_4) spectrum of thiophene-2-carboxylic acid (**2t**).

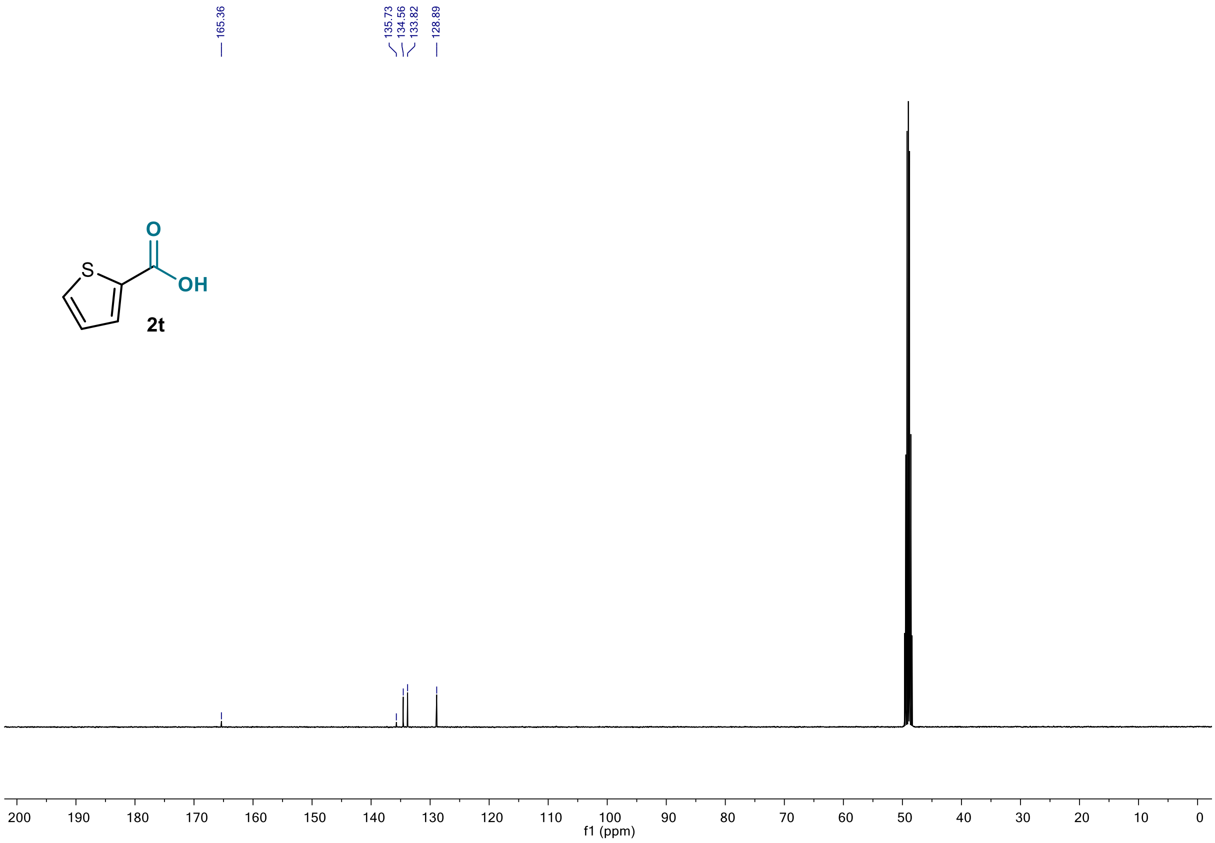

Figure S58: ^{13}C NMR (101 MHz, Methanol-d_4) spectrum of thiophene-2-carboxylic acid (**2t**).

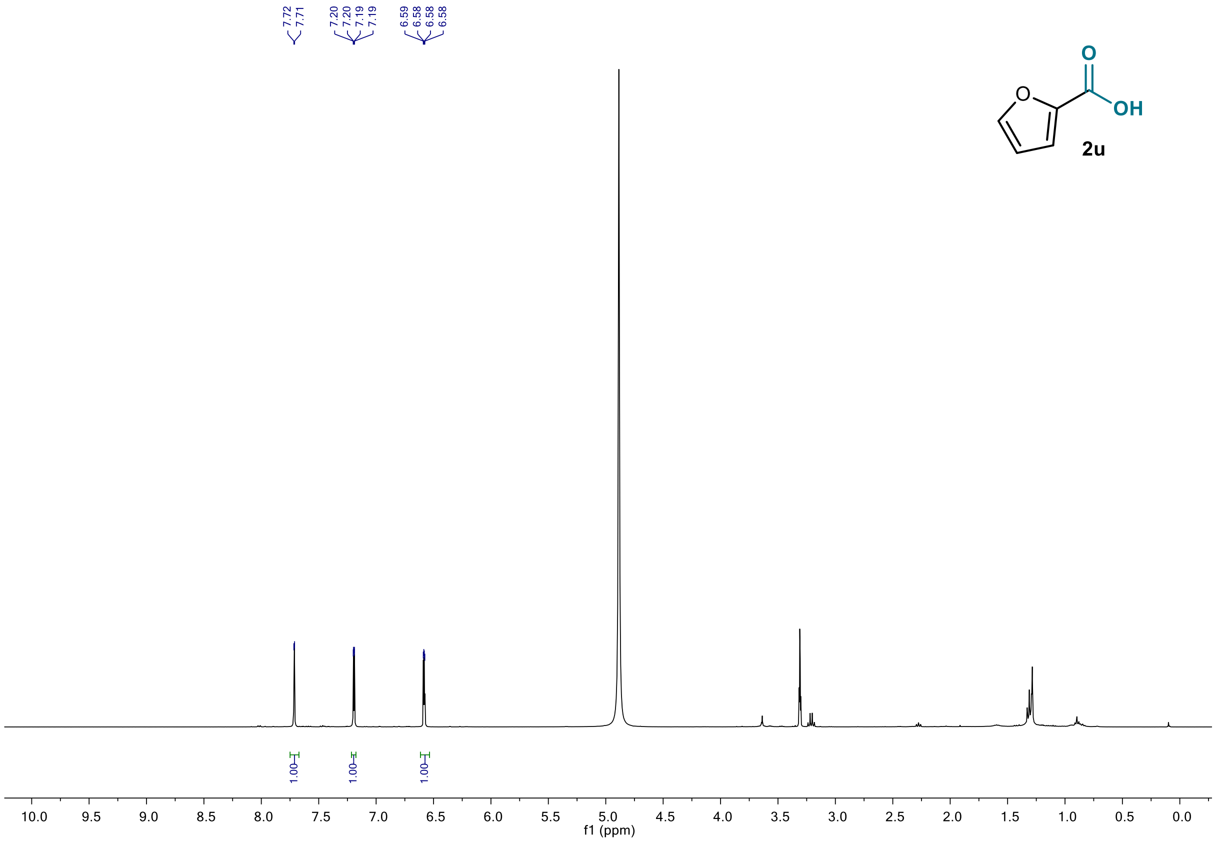

Figure S59: ^{1}H NMR (400 MHz, Methanol-d_4) spectrum of furan-2-carboxylic acid (**2u**).

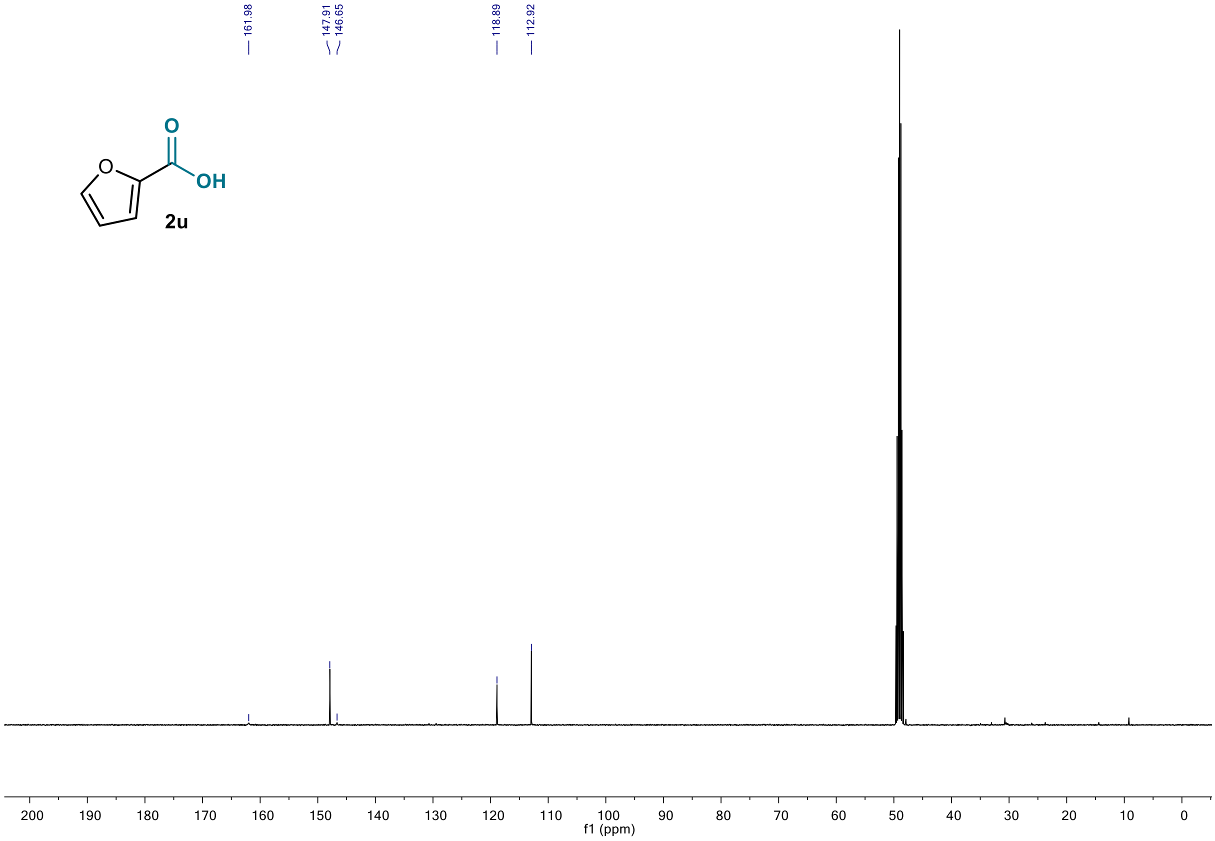

Figure S60: ^{13}C NMR (101 MHz, Methanol-d_4) spectrum of furan-2-carboxylic acid (**2u**).

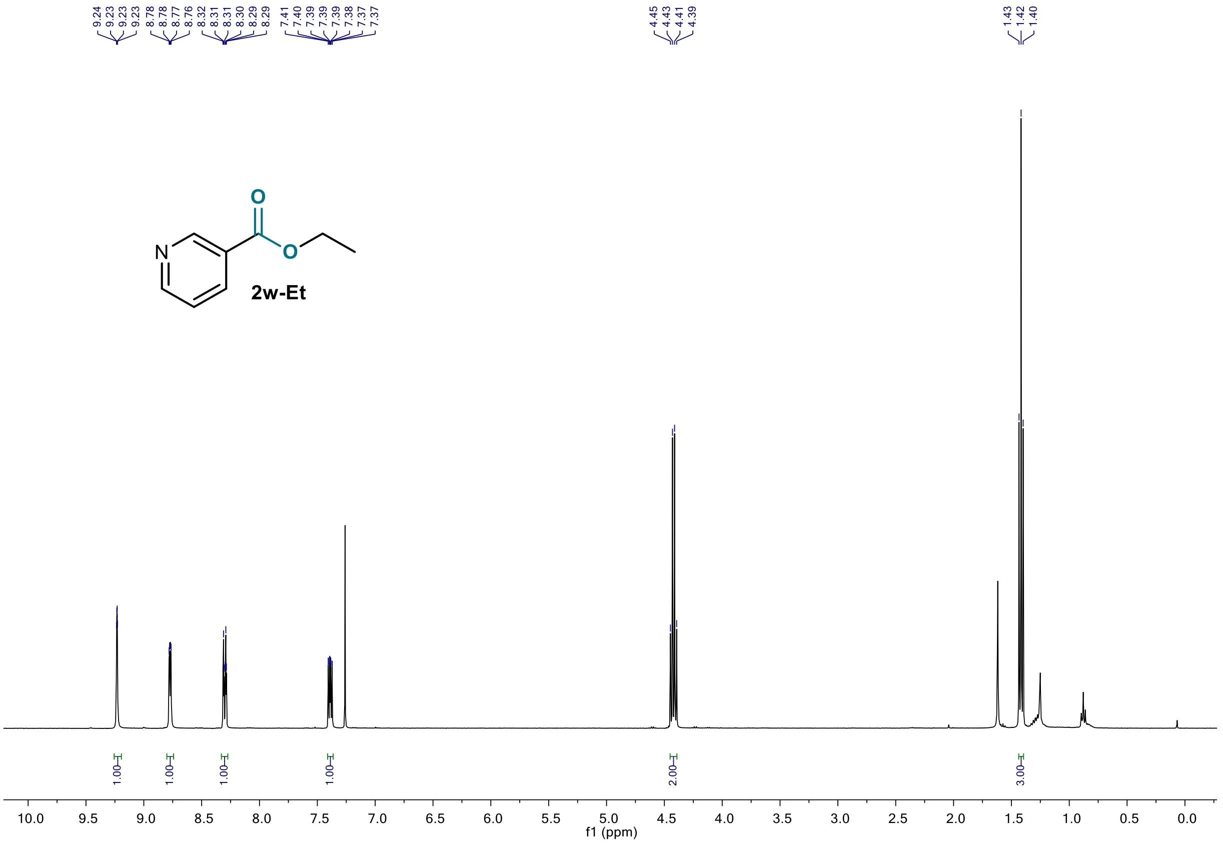

Figure S61: ^{1}H NMR (400 MHz, Methanol-d_4) spectrum of ethyl nicotinate (**2w-Et**).

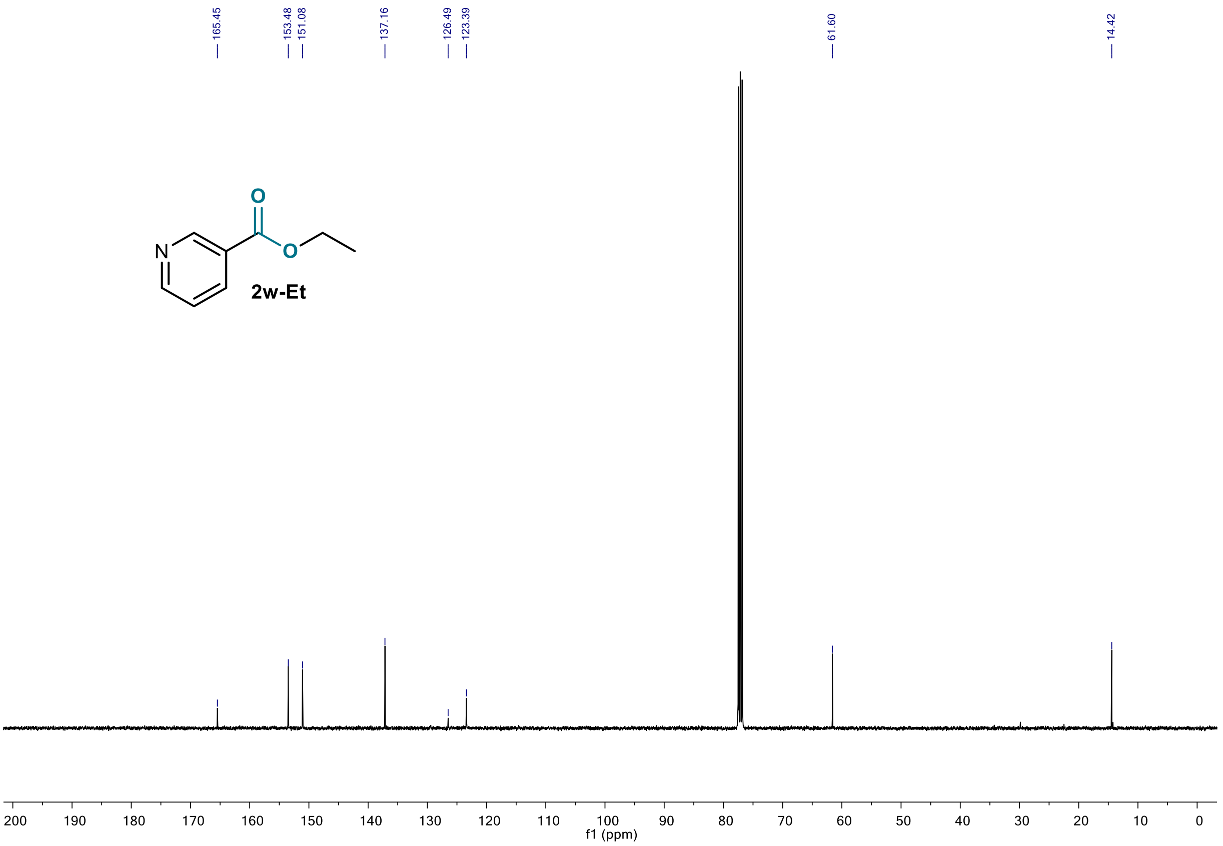

Figure S62: ^{13}C NMR (101 MHz, Methanol-d_4) spectrum of ethyl nicotinate (**2w-Et**).

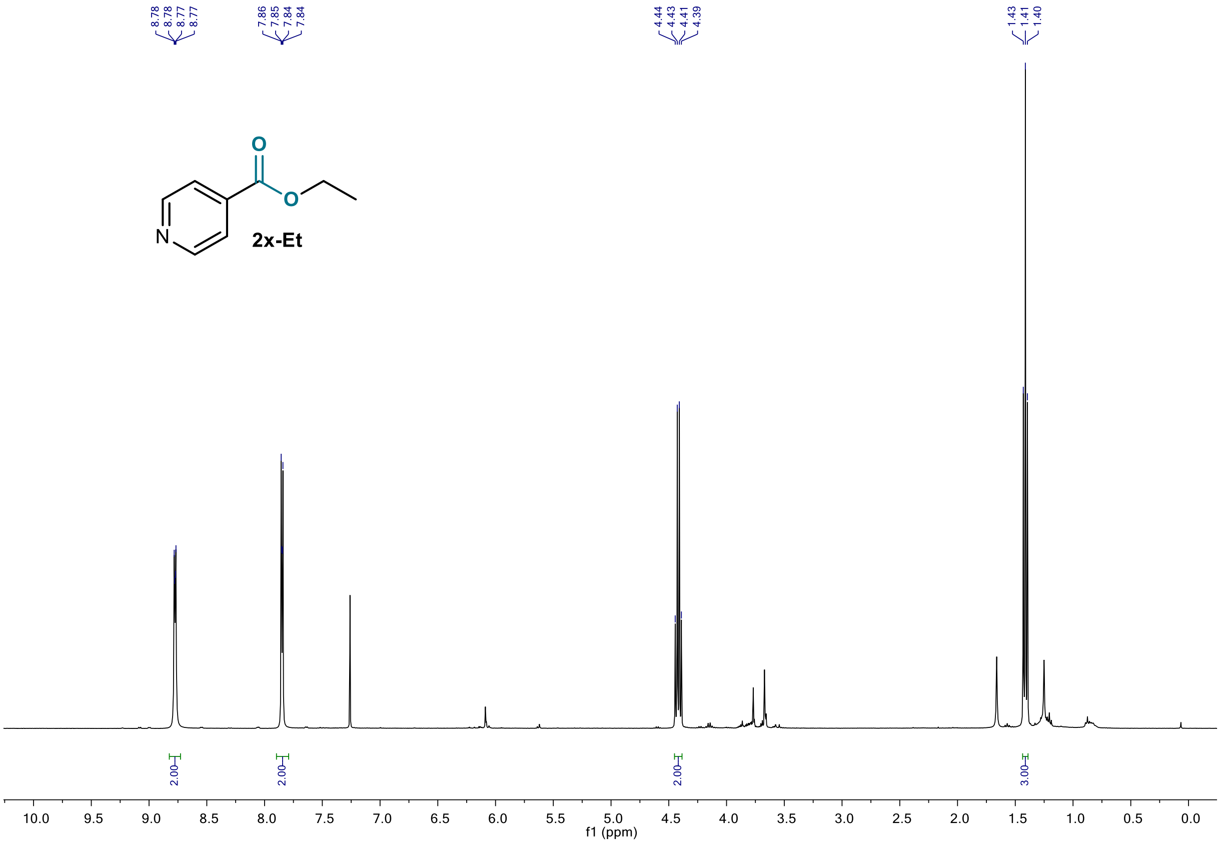

Figure S63: ^{1}H NMR (400 MHz, Methanol-*d*4) spectrum of ethyl isonicotinate (**2x-Et**).

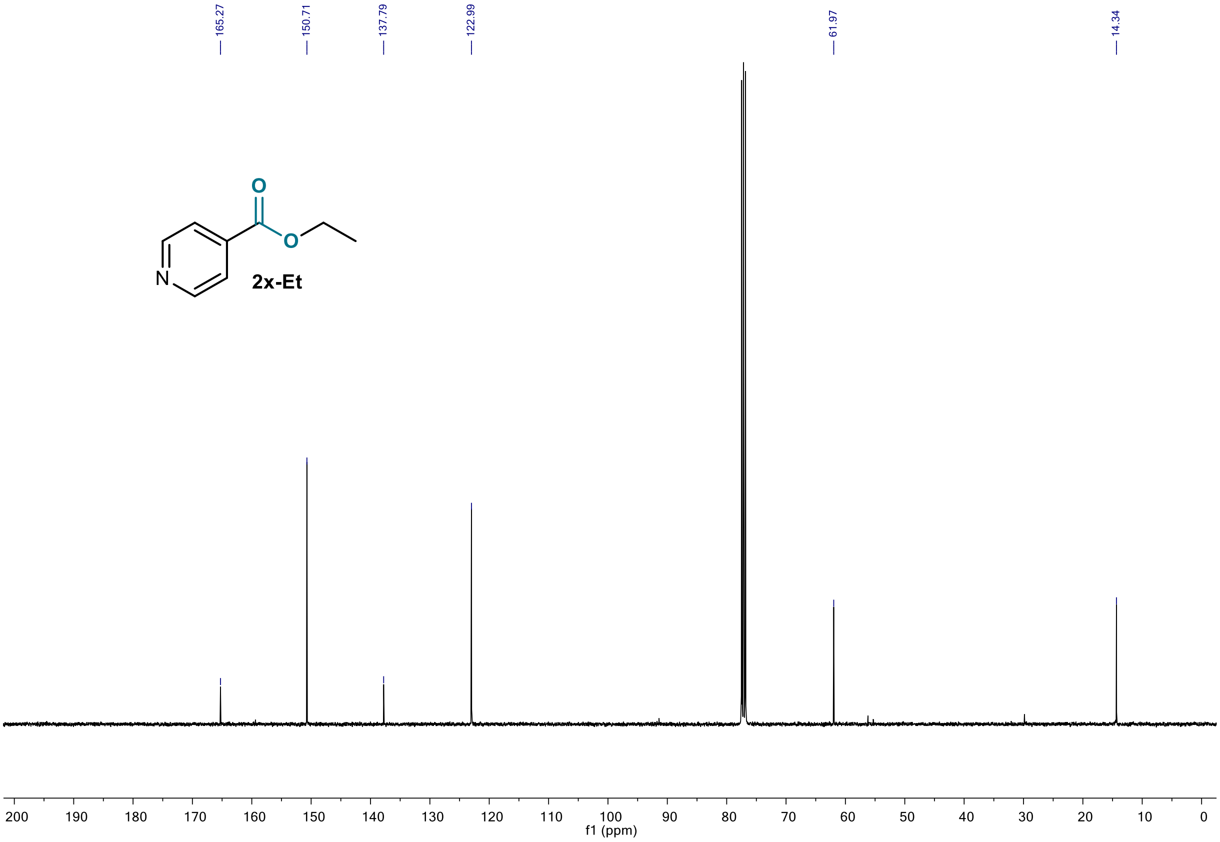

Figure S64: ^{13}C NMR (101 MHz, Methanol-*d*4) spectrum of ethyl isonicotinate (**2x-Et**).

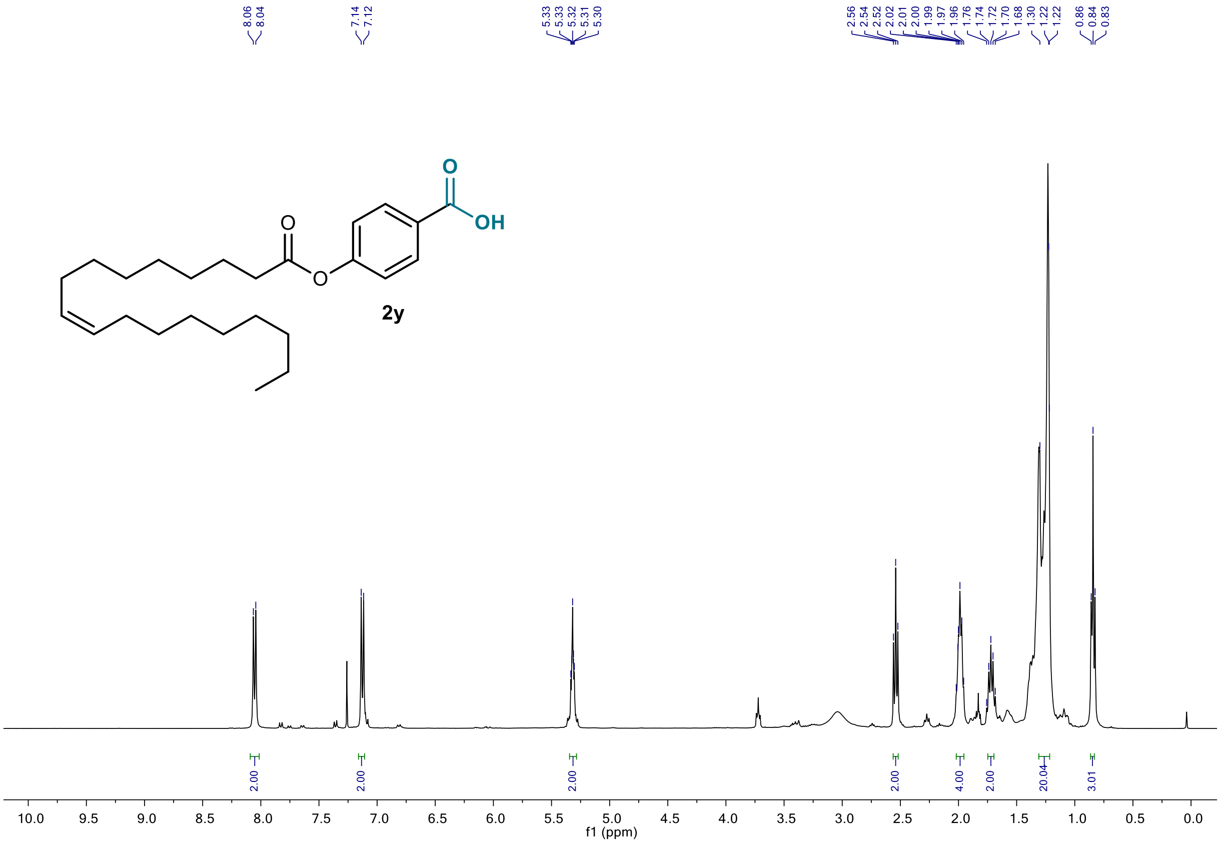

Figure S65: ^{1}H NMR (400 MHz, Methanol-d_4) spectrum of ethyl 4-(oleoyloxy)benzoic acid (**2y**).

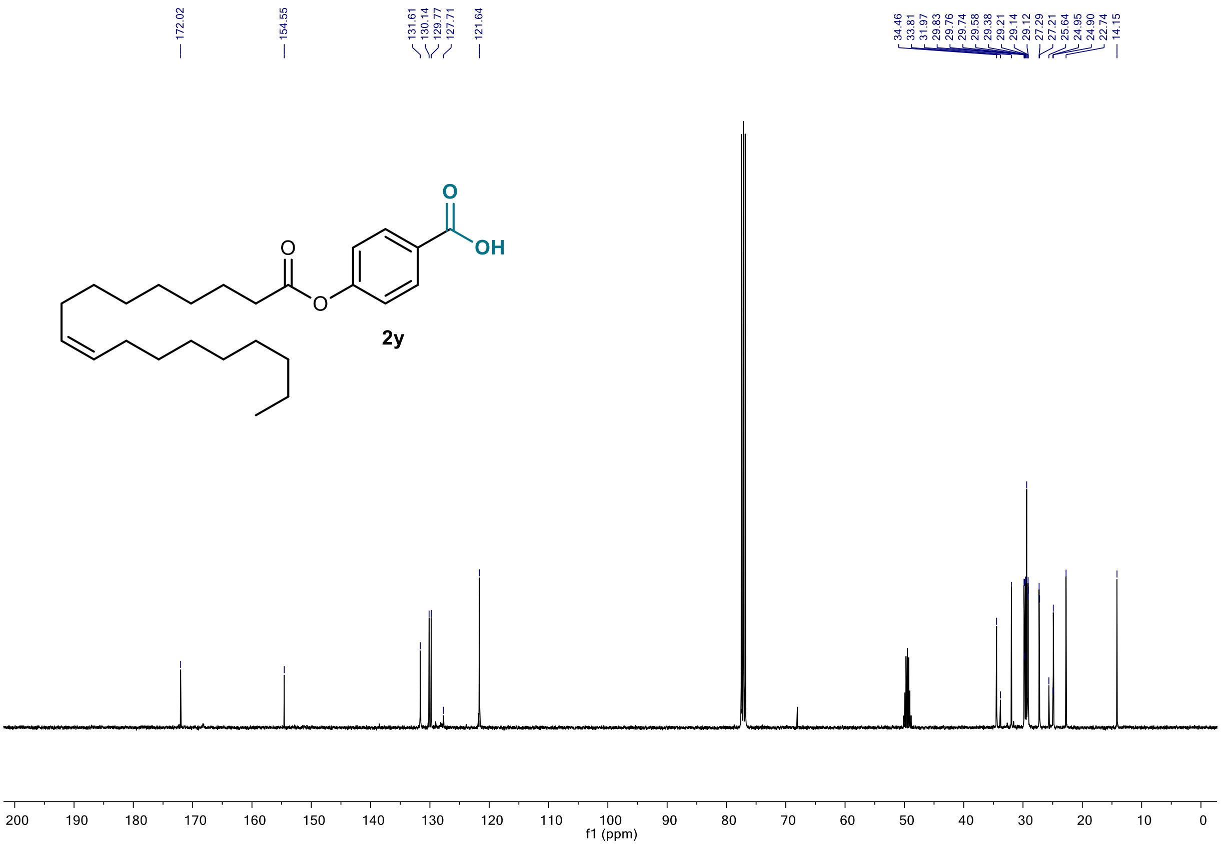

Figure S66: ^{13}C NMR (101 MHz, Methanol-d_4) spectrum of ethyl 4-(oleoyloxy)benzoic acid (**2y**).

9. References

[1] A. Thevenon, A. Rosas-Hernández, J. C. Peters, T. Agapie, *Angew. Chem. Int. Ed.* **2019**, *58*, 16952-16958.

[2] A. Carretero-Cerdán, S. Carrasco, A. Sanz-Marco, A. Jaworski, B. Martín-Matute, *Materials Today Chemistry* **2023**, *31*, 101618.

[3] T. Mita, K. Suga, K. Sato, Y. Sato, *Org. Lett.* **2015**, *17*, 5276-5279.

[4] G. T. Potter, G. C. Jayson, G. J. Miller, J. M. Gardiner, *Tetrahedron Lett.* **2015**, *56*, 5153-5156.

[5] A. K. Shil, S. Kumar, C. B. Reddy, S. Dadhwal, V. Thakur, P. Das, *Org. Lett.* **2015**, *17*, 5352-5355.

[6] L. Pehlivan, E. Métay, S. Laval, W. Dayoub, P. Demonchaux, G. Mignani, M. Lemaire, *Tetrahedron* **2011**, *67*, 1971-1976.

[7] K. Moriyama, M. Takemura, H. Togo, *Org. Lett.* **2012**, *14*, 2414-2417.

[8] J. Zhao, C. Mück-Lichtenfeld, A. Studer, *Adv. Synth. Catal.* **2013**, *355*, 1098-1106.

[9] H. P. Kalmode, K. S. Vadagaonkar, S. L. Shinde, A. C. Chaskar, *J. Org. Chem.* **2017**, *82*, 3781-3786.

[10] R. Zhou, M. Yang, Y. Li, D. Li, *ChemistrySelect* **2021**, *6*, 11951-11953.

[11] I. Chiarotto, M. Feroci, G. Sotgiu, A. Inesi, *Tetrahedron* **2013**, *69*, 8088-8095.
